# Simple and flexible sign and rank-based methods for testing for differential abundance in microbiome studies

**DOI:** 10.1371/journal.pone.0292055

**Published:** 2023-09-26

**Authors:** Leyla Kodalci, Olivier Thas

**Affiliations:** 1 Data Science Institute and I-BioStat, Hasselt University, Diepenbeek, Belgium; 2 Department of Applied Mathematics, Computer Science and Statistics, Ghent University, Gent, Belgium; 3 National Institute for Applied Statistics Research Australia (NIASRA), University of Wollongong, Wollongong, New South Wales, Australia; University of Arizona, UNITED STATES

## Abstract

Microbiome data obtained with amplicon sequencing are considered as compositional data. It has been argued that these data can be analysed after appropriate transformation to log-ratios, but ratios and logarithms cause problems with the many zeroes in typical microbiome experiments. We demonstrate that some well chosen sign and rank transformations also allow for valid inference with compositional data, and we show how logistic regression and probabilistic index models can be used for testing for differential abundance, while inheriting the flexibility of a statistical modelling framework. The results of a simulation study demonstrate that the new methods perform better than most other methods, and that it is comparable with ANCOM-BC. These methods are implemented in an R-package ‘signtrans’ and can be installed from Github (https://github.com/lucp9827/signtrans).

## Introduction

A microbiome contains the collective genome of microbial cells that interact with a particular host. The microbiome plays a key role in various host functions contributing to the overall health and fitness of the host, e.g. the plant microbiome can increase plant growth, stress tolerance, and disease resistance [1]. Developments in high-throughput sequencing and new bioinformatics tools have already provided a wealth of new knowledge and insights in the human microbiome. To date, accumulating evidence in human microbiome studies have demonstrated several associations between disruptions of the human microbiome composition and pathologic conditions [2].

A very common method to characterize microbial species in the human microbiome is sequencing the 16S rRNA marker gene. These 16S sequences are clustered into operational taxonomic units (OTUs) based on high sequence similarity and they are eventually mapped to reference genomes for the identification of the species. The outcome of the microbiome characterization is a count table with counts related to the abundances of the microbial species in the sample. Alternatively, an Amplicon Sequence Variants (ASV) analysis can be performed on the sequencing reads, resulting in an ASV count table. Also these counts are proxies of the relative abundances and can be analysed just like the OTU counts. In microbiome studies, the relative abundances of microbial species can be reported at any taxonomic rank. Instead of using specific terms like OTU, species or family, which refer to a specific taxonomic rank, we will use the generic term taxon.

Microbiome data analysis encounters many challenges and despite many recent developments there is still room for improvement. For example, as a result of high-throughput sequencing, the data are high-dimensional with up to thousands of taxa, exceeding the number of samples. Moreover, microbiome data have a compositional structure characterized by a sum-constraint (the sum of the counts equals a constant). This constraint is a result of sequencing methods having a fixed upper bound on the number of sequences processed (i.e. the library size); this library size is hard to control in the laboratory and varies between experiments. Hence, relevant information can only be obtained from the ratios, i.e. the relative abundances. We refer to [3–5] for detailed discussions on this issue. Furthermore, microbiome data are sparse (many zero abundances), overdispersed due to biological variability, and have varying library sizes between samples. These characteristics make it difficult to compare samples; see e.g. [6, 7].

Many statistical methods in microbiome data analysis aim to identify differentially abundant taxa, i.e. taxa that have on average different relative abundances between two or more conditions. These differentially abundant taxa are of specific interest as they can be used in intervention studies or as disease biomarkers. The statistical methods must be sufficiently powerful to be useful, and they should take the structure of the data into account so as to prevent too many false discoveries in this high-dimensional setting.

Nowadays, there are numerous statistical methods available for differential abundance testing. Many classical statistical tests such as the two-sample t-test or the non-parametric Wilcoxon-Mann-Whitney (WMW) test are still widely used for differential abundance testing. However, these classical tests do not take the structure of microbiome data into account. Other differential abundance methods rely on distributional assumptions, e.g. the negative binomial (NB) or the zero-inflated NB (ZINB); see e.g. [8] and the popular R packages EdgeR [9] and DESeq2 [10] which are also still frequently used for microbiome data analysis. However, a recent study by Hawinkel and colleagues [11] demonstrates that these assumptions are violated for the majority of taxa, which partly explains why (ZI)NB-based methods have problems controlling the false discovery rate (FDR). By removing or adjusting for technical between-sample variability (e.g. differences in sequencing depths), the sensitivity and FDR control can be improved. This may be accomplished by controlling for these effects in the statistical model or by preprocessing the data via a normalization factor. We refer to [12] for a detailed discussion on the ecological meaning of such normalisation factors.

Another class of methods makes use of compositional data analysis (CoDa) methods, which form a well-established mathematically supported class of methods for compositional data [13–15]. Nonetheless, many CoDa methods use log-transforms of (ratios of) counts, which causes problems due to the sparse nature of microbiome data. ALDEx2 is an example of such a method for microbiome data analysis [16]. A typical solution exists in adding an arbitrary constant to the count observations before computing the log-ratios, but this is an ad hoc method that is not supported by theory. The zero-issue and the ad-hoc addition of a constant also arises in the ANCOM-BC method [17]. This method accounts for the compositionality by introducing a sampling fraction, which, when the model assumptions hold, allows for inference at the level of the absolute abundances.

In this paper, we aim to demonstrate that some well chosen sign transformations allow for valid inference with compositional data. The term “sign transformation” is inspired by the sign test statistic, which makes use of the “sign” of an observation’s deviation from the median. We use sign-transformation in a slightly more general sense: it is a transformation of one or more observations into a 0/1 outcome. These sign-transformed outcomes are combined with logistic regression and probabilistic index models to provide estimates of informative parameters and to generate tests for differential abundance, resulting in robust distribution-free methods that can deal with an excess of zeros and overdispersion. The flexibility of the regression model frameworks allow for inclusion of other factors (e.g. study center) or covariates (e.g. age). The resulting estimates and test statistics are related to sign and rank statistics.

In the methods section, we will present two types of sign-transformations and we refer to S1 Text to demonstrate that they satisfy the three basic properties that are required for valid compositional data analysis. Based on these sign transformations, we build semiparametric statistical models for which we show how the parameters can be estimated and how inference can be performed (e.g. testing for differential abundance). These will turn out to be easy, because we can rely on existing implementations of logistic regression (LR) and probabilistic index models (PIM) [18]. In the following sections, the methods are evaluated in a simulation study and a case study is presented. All proposed methods are available in the R package signtrans, which can be installed from https://github.com/lucp9827/signtrans.

## Methods

### From log-ratios to S-sign and R-sign transformations

We represent the read count of taxon *t* (*t* = 1, …, *m*) in sample *i* (*i* = 1, …, *n*) as *N*_*it*_, and the sum $\sum_{t=1}^{m}N_{it}=L_{i}$ is referred to as the library size of sample *i*. Traditional compositional data analysis (CoDa) transforms the counts to log-ratios of the form ln(*N*_*it*_/*R*_*i*_), where *R*_*i*_ can be either the geometric mean of all counts in sample *i*, or the count of a single taxon (a reference taxon). The former transformation is known as the central log-ratio (CLR) and the latter as the additive log-ratio (ALR). Another choice for *R*_*i*_ can be the arithmetic mean or median of the counts of a set of reference taxa. Ma and colleagues [19], for example, proposed a network-based approach for selecting a group of relatively invariant microbial species across samples and conditions. In other words, they select a group of taxa whose relative abundance or relative change has low variation across all samples and conditions. These approximately invariant taxa can be used as reference taxa. To make this more explicit, let $T_{R}$ denote the index set referring to the reference taxa $t\in T_{R}$. We write

$$R_{i}=\text{median}\left\{N_{it}:t\in T_{R}\right\}.$$

The reference set of taxa is referred to as the *reference frame*, as in [20].

The many zero counts in microbiome data, however, jeopardise the log-ratio analyses. The classical solution exists in adding a constant pseudo-count to all counts, but the pseudo-count is an arbitrary constant which affects the outcome of the data analysis and hence it may introduce bias into the analysis. The advantage of the log-ratio transforms, however, is that they satisfy the three basic properties required for proper compositional data analysis [21]: (1) scale invariance; (2) subcompositional coherence; (3) permutation invariance. We refer to [22] for a detailed discussion on these properties.

We will discuss two types of data transformations. The first, which will be referred to as the S-sign, is of the form

$$\begin{array}{c} I_{it}^{S}=\text{I}\{N_{it}≼R_{i}\} \end{array}$$ (1)

and the second, which will be referred to as the R-sign, is of the form

$$\begin{array}{c} I_{ijt}^{R}=\text{I}\{N_{it}/R_{i}≼N_{jt}/R_{j}\}, \end{array}$$ (2)

where I {*a* ≼ *b*} is defined as I {*a* ≼ *b*} = I {*a* < *b*} + 0.5I {*a* = *b*}.

The terminology *S-sign* and *R-sign* inherit their names from the classical sign (S) and rank (R) statistics. In particular, a statistic of the form $\sum_{i=1}^{n}I_{it}^{S}$ resembles the sign test statistic (in which *R*_*i*_ is replaced by the median). The R-signs can be used for constructing rank statistics, because the rank of the relative abundance *N*_*jt*_/*R*_*j*_ can be written as $\sum_{i=1}^{n}I_{ijt}^{R}$.

In S1 Text, we demonstrate that the use of these sign transformations in combination with the models and estimators that we propose in the following sections, satisfy the three principles of compositional data analysis. However, here we will already argue that the sign-transforms of Eqs (1) and (2) can deal with zero counts. The S-sign does not involve a ratio or a logarithm, and the R-Sign can be rewritten as $I_{ijt}^{R}=\text{I}\{R_{j}N_{it}≼R_{i}N_{jt}\}$, and so again zeroes do not prohibit its calculation. Many zeroes, on the other hand, will result in many uninformative sign transforms (cfr. ties), but this does not invalidate the statistical inference.

### Null hypotheses

We will consider four null hypotheses that can be tested with the sign transforms. We will drop the taxon index *t* and sample index *i* for notational comfort. Hence, the notation (*N*, *R*, *A*, *L*) refers to the joint distribution of the counts *N* of a taxon, the median count *R* of the reference frame (RF), the 0/1 binary treatment assignment *A* and the library size *L*. For expressing the hypotheses related to the R-signs we will also need (N*, R*, A*, L*), which is independently distributed, but has the same joint distribution as (*N*, *R*, *A*, *L*).

In the following paragraphs we formulate several null hypotheses, which will all involve probabilities of the form P {*A* ≼ *B*} for a random vector (*A*, *B*). In analogy with the definitions of the signs, this probability is defined as P {*A* ≼ *B*} = P {*A* < *B*} + 0.5P {*A* = *B*}. This probability is known as a probabilistic index. In the following, we will often loosely translate this into the probability that *A* is smaller than *B*. The probabilities involved in the following null hypotheses also serve as effect sizes. See S2 Text, where we illustrate their interpretation by showing their relationship to the log fold change (LFC).

#### Marginal-S null hypothesis

$$\begin{array}{c} H_{0}:\text{P}\{N≼R|A=0\}=\text{P}\{N≼R|A=1\} \end{array}$$ (3)

This hypothesis expresses that the probability that the abundance of the taxon is smaller than the median abundance in the RF is the same in both treatment groups. The term “marginal” refers to the fact that the probabilities are not conditional on any sample-specific covariates.

#### Marginal-R null hypothesis

$$\begin{array}{c} H_{0}:\text{P}\{\frac{N}{R}≼\frac{N^{*}}{R^{*}}|A=0,A^{*}=1\}=\frac{1}{2} \end{array}$$ (4)

This hypothesis expresses that the probability that the relative abundance of the taxon in treatment group 0 is smaller than its relative abundance in treatment group 1, equals 50%.

#### Conditional-S null hypothesis

The conditional-S null hypothesis,

$$\begin{array}{c} H_{0}:\text{P}\{N≼R|A=0,L=l\}=\text{P}\{N≼R|A=1,L=l\} \end{array}$$ (5)

for all l, expresses that the probability that the abundance of the taxon is smaller than the median abundance in the RF is the same in both treatment groups, given that both library sizes are equal to *l*.

#### Conditional-R null hypothesis

$$\begin{array}{c} H_{0}:\text{P}\{\frac{N}{R}≼\frac{N^{*}}{R^{*}}|A=0,A^{*}=1,L=L^{*}\}=\frac{1}{2} \end{array}$$ (6)

This hypothesis expresses that the probability that the relative abundance of the taxon in treatment group 0 is smaller than its relative abundance in treatment group 1, equals 50%, given that the library sizes are equal.

### Semiparametric models

We now explain how the hypotheses formulated in the previous subsection can be tested with the S and R signs. This will involve established models and parameter estimation theory. In the context of our applications, these models can be considered as semiparametric because they do not impose strong distributional assumptions on (*N*, *R*, *A*, *L*) or on (*N*, *R*) ∣ (*A*, *L*). This will be explained in some more detail after the models have been introduced.

#### Logistic regression model for the marginal-S null hypothesis

The marginal-S null hypothesis (3) can be tested by fitting a logistic regression model with the S-sign transforms as outcome variables. In particular, logit (P {*N* ≼ *R* ∣ *A*}) = *β*_0_ + *β*_*A*_*A*. The original null hypothesis now becomes *H*_0_ : *β*_*A*_ = 0. Upon using the S-sign transforms $I_{i}^{S}$ as outcome data, and the corresponding treatment group indicators *A*_*i*_, all assumptions required for valid inference with the logistic regression model and maximum likelihood parameter estimation methods, are satisfied.

#### Logistic regression model for the conditional-S null hypothesis

The conditional-S null hypothesis (5) can be tested by fitting a logistic regression model with the S-sign transforms as outcome variables and *A* and *L* as regressors, logit (P {*N* ≼ *R* ∣ *A*, *L*}) = *β*_0_ + *β*_*A*_*A* + *β*_*L*_*L*. The original null hypothesis now becomes *H*_0_ : *β*_*A*_ = 0. When the linearity and additivity of the model are correct, all assumptions required for valid inference within the maximum likelihood framework, are satisfied. The model can be further extended by adding other covariates or factors (e.g. confounders, blocking or stratification factors). We can thus make use of the flexibility of the logistic regression framework.

#### Probabilistic index models for the marginal-R null hypothesis

The marginal-R null hypothesis (4) can be tested by fitting a probabilistic index model (PIM) [18] with *N*/*R* as outcome variables. In particular, $\text{logit}\;(\text{P}\{\frac{N}{R}≼\frac{N^{*}}{R^{*}}|A,A^{*}\})=\beta_{A}\left(A^{*}-A\right)$. The original null hypothesis now becomes *H*_0_ : *β*_*A*_ = 0. Note that considering *N*/*R* as outcome variable is equivalent to using the R-sign transforms as pseudo-observations in the estimating equations of the PIM (see Section 3.1 of [18]).

#### Probabilistic index models for the conditional-R null hypothesis

The conditional-R null hypothesis (6) can be tested by fitting a probabilistic index model of the form $\text{logit}\;(\text{P}\{\frac{N}{R}≼\frac{N^{*}}{R^{*}}|A,A^{*},L,L^{*}\})=\beta_{A}\left(A^{*}-A\right)+\beta_{L}\left(L^{*}-L\right)$. The original null hypothesis now becomes *H*_0_ : *β*_*A*_ = 0. The interpretation of the parameter *β*_*A*_ comes from restricting the model to *A* = 1, A* = 0 and *L* = L*, from which we find $\text{expit}\;(\beta_{A})=\text{P}\{\frac{N}{R}≼\frac{N^{*}}{R^{*}}|A=1,A^{*}=0,L=L^{*}\}$. The validity of the inference relies on the additivity and linearity of the model. Also here we can maximally make use of the flexibility of PIMs, and include extra covariates or factors.

#### Regression imputation estimators for the marginal-S null hypothesis

A disadvantage of the conditional approach may be that adding a regressor to a logistic regression model or PIM does not necessarily increase the power of the test for *H*_0_ : *β*_*A*_ = 0 [23]. Moreover, these models are generally not collapsible [24]. Hence, when further sample-specific covariates are added to the regression model, the interpretation of the parameter *β*_*A*_ will change accordingly. Moreover, the original research question will often be most naturally translated into the marginal null hypothesis (3) or (4), irrespective of the presence of covariates in the data set.

In the semiparametric literature estimators have been proposed that allow for the use of covariates, while still estimating a parameter that has the marginal effect interpretation [25, 26]. These estimators still refer to the marginal effect size, while having increased efficiency by using the covariate information. Such estimators have been proposed by [27, 28] for logistic regression models, but they were particularly developed in the context of randomised clinical trials and their validity depends on the randomisation assumption. With *A* and *L* the usual notation for treatment assignment and library size, respectively, and with *X* (vector of) other covariates, the randomisation assumption is equivalent to *A* ⫫ (*L*, *X*). Note, however, that in the context of microbiome studies and in the absence of *X*, this assumption becomes *A* ⫫ *L*, which is satisfied even when the treatment assignment is not random. The reason is that the library size *L* is a technical source of variability that does not depend on the treatment assignment.

This estimator is referred to as the regression imputation (RI) estimator. It uses predictions from conditional models so that covariate information is used, but the final estimator averages out the conditioning so that the marginal effect size is still targeted. In the literature, the RI estimator is known by several alternative names, among which the targeted maximum likelihood estimator [28], standardized estimator [29], and augmented estimator [30]. We implemented the RI estimator of Moore and van der Laan [28]:

1. Fit a logistic regression model with the S-Sign transform as outcome, and with an intercept and main effects for the treatment indicator *A* and the library size *L*. Let $\hat{p}\left(A,L\right)=\text{expit}\;({\hat{\beta }}_{0}+{\hat{\beta }}_{A}A+{\hat{\beta }}_{L}L)$ denote the estimate of the probability P {*N* ≼ *R* ∣ *A*, *L*}.
2. Let $\hat{\pi }\left(a\right)=\frac{1}{n}\sum_{i=1}^{n}\hat{p}\left(a,L_{i}\right)$, *a* = 0, 1.
3. The RI estimator of the odds ratio for the marginal effect of the treatment is given by $\text{exp}\left({\hat{\beta }}_{A}^{\text{RI}}\right)=[\hat{\pi }\left(1\right)/\left(1-\hat{\pi }\left(1\right)\right)]/[\hat{\pi }\left(0\right)/\left(1-\hat{\pi }\left(0\right)\right)]$.
4. The variance of ${\hat{\beta }}_{A}^{\text{RI}}$ is estimated as ${\hat{\sigma }}_{A}^{2}=\frac{1}{n^{2}}\sum_{i=1}^{n}{\text{IC}}_{i}^{2}$ with
  $$\begin{array}{ccc} {\text{IC}}_{i} & = & \frac{1}{\hat{\pi }\left(1\right)\left(1-\hat{\pi }\left(1\right)\right)}(\frac{A_{i}}{\delta }\left(I_{i}^{S}-\hat{p}\left(1,L_{i}\right)\right)+\hat{p}\left(1,L_{i}\right)-\hat{\pi }\left(1\right))\\ & & -\frac{1}{\hat{\pi }\left(0\right)\left(1-\hat{\pi }\left(0\right)\right)}(\frac{1-A_{i}}{1-\delta }\left(I_{i}^{S}-\hat{p}\left(0,L_{i}\right)\right)+\hat{p}\left(0,L_{i}\right)-\hat{\pi }\left(0\right)) \end{array}$$
  and $\delta =\sum_{i=1}^{n}A_{i}/n$.

Testing for no treatment effect, $H_{0}:\beta_{A}^{\text{RI}}=0$, makes use of the test statistic ${\hat{\beta }}_{A}^{\text{RI}}/{\hat{\sigma }}_{A}$, which is asymptotically standard normal under the null hypothesis. The variance estimator can be improved by applying a non-parametric bootstrap procedure to estimate the sampling distribution of ${\hat{\beta }}_{A}^{\text{RI}}$.

#### Regression imputation estimators for the marginal-R null hypothesis

For PIMs RI estimators of the marginal effect size have been proposed by Vermeulen, Thas, and Vansteelandt [31]. Their theory also only holds for random treatment assignment, but, as before, the condition *A* ⫫ *L* is sufficient when no other covariates are included and this condition always holds in microbiome studies. The estimator of the marginal effect size $\beta_{A}^{\text{RI}}=\text{P}\{\frac{N}{R}≼\frac{N^{*}}{R^{*}}|A=0,A^{*}=1\}$ and its variance estimator are computed as follows:

1. Fit a PIM with main effects for the treatment indicator *A* and library size *L*. Let $\hat{p}\left(L_{1},L_{2}\right)=\text{expit}\;({\hat{\beta }}_{A}+\beta_{L}\left(L_{2}-L_{1}\right))$ denote an estimate of the probability $\text{P}\{\frac{N}{R}≼\frac{N^{*}}{R^{*}}|A=0,A^{*}=1,L=L_{1},L^{*}=L_{2}\}$.
2. The marginal effect size is then estimated as
  $${\hat{\beta }}_{A}^{\text{RI}}=\frac{1}{n(n-1)}\sum_{i=1}^{n}\sum_{j\neq i}\hat{p}\left(L_{j},L_{i}\right).$$
3. The variance of ${\hat{\beta }}_{A}^{\text{RI}}$ is estimated as ${\hat{\sigma }}_{A}^{2}=\frac{1}{n^{2}}\sum_{i=1}^{n}{\text{IC}}_{i}$ with
  $$\begin{array}{ccc} {\text{IC}}_{i} & = & \frac{1-A_{i}}{1-\delta }(\frac{1}{n-1}\sum_{j\neq i}\frac{A_{j}.I_{ij}^{R}}{\delta })+\frac{A_{i}}{\delta }(\frac{1}{n-1}\sum_{j\neq i}\frac{\left(1-A_{j}\right).I_{ji}^{R}}{1-\delta })-2{\hat{\beta }}_{A}^{\text{RI}}\\ & & +\left(A_{i}-\delta \right)[\frac{1}{n-1}\sum_{j\neq i}(\frac{\hat{p}\left(L_{i},L_{j}\right)}{1-\delta }-\frac{\hat{p}\left(L_{j},L_{i}\right)}{\delta })] \end{array}$$
  where $\delta =\sum_{i=1}^{n}A_{i}/n$.

Testing for no treatment effect can be done with the test statistic ${\hat{\beta }}_{A}^{\text{RI}}/\hat{\sigma }$, which asymptotically has a standard normal distribution under the null hypothesis.

### Some notes on the practical implementation

Selecting an appropriate reference frame in microbiome data is challenging, particularly because of the typical characteristics of microbiome data (e.g. compositionality, sparsity, overdispersion). If prior information exists on taxa that are uniformly distributed across samples and conditions, and thus do not relate to the condition, these taxa can be used as reference taxa. However, this information is often not available. For the sign methods, we have chosen RioNorm2 for selecting the reference frame [19]. RioNorm2 selects a group of taxa for which the relative abundances have low variation across all samples and conditions, under the assumption that most taxa are not differentially abundant. It comes with the recommendation to only use taxa that are present in at least 80% of the samples with an average count larger than 5. Since this reference count *R* is by construction very often larger than the count *N* of an individual taxon (e.g. a rare taxon), the S-sign I {*N* ≼ *R*} is very often zero. We, therefore, suggest to normalise the RF count by multiplying *R* with the median of the read counts of the target taxon, divided by the median of the counts of the RF.

The S-sign methods are implemented using logistic regression models, which can cause problems when there is complete or quasi-complete separation. This separation is caused by the grouping variable *A* perfectly predicting the outcome variable. A solution to this problem was fitting logistic regression models using Firth’s bias reduction method [32].

## Simulation study

Simulations were used to evaluate the performance of the new methods in terms of the sensitivity, type I error rate and the control of the false discovery rate (FDR).

All analyses were conducted with R version 4.0.3 [33]. Table 1 in S1 Appendix specifies the details of the R packages used in this simulation study. Additionally, diagnostic plots of the SPsimSeq simulated data are provided (Figs 4–11 in S1 Appendix), comparing important characteristics of the simulated data with the source data. Overall, these plots show that the simulated data mimics the source data well. Small differences between the simulated and source data reflect the settings of the various scenarios, e.g. in setting B, the number of not-differentially abundant taxa was too small in the source data set. It was therefore required to sample these not-differentially abundant taxa with replacement, which affects the variance of the taxa abundance levels in the simulated data.

### Simulation study set up

As a first simulation framework, we use the SPSimSeq simulation method [34] for simulating realistic data. This method starts from a real dataset (source data). Our source data originates from a study on the effect of diet on the human gut microbiome [35], referred to as HGM data. Only the 1000 most abundant taxa were used to start with.

**Fig 1:**
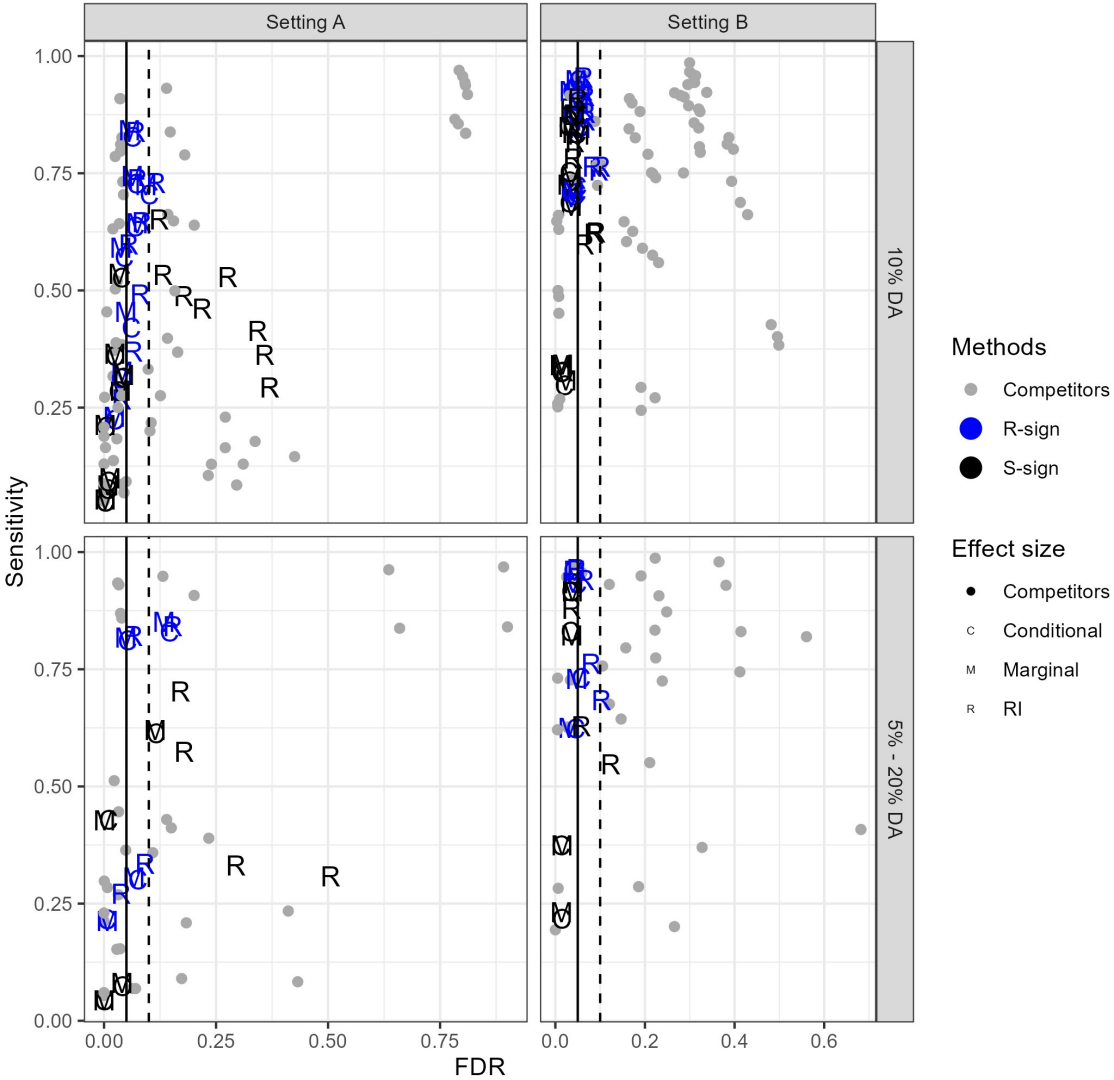
Empirical FDRs vs sensitivities of the sign methods and competitors for the various simulation scenarios with SPSimSeq. The top row contains the results of scenarios with 10% DA taxa. The bottom row contains the results of the most extreme scenarios with 5% and 20% taxa. Left: Setting A (high sparsity) and Right: Setting B (low sparsity).

The HGM data was further preprocessed by filtering out low abundant taxa. Two filtering strategies were implemented. In setting A, all taxa that were present in at most one sample were removed, which produced data with high sparsity (±77% of the counts are zero). In setting B, all taxa that were present in at most five samples were removed, resulting in data with low to moderate sparsity (±39% zeroes). The two preprocessed data sets were used as source data. SPsimSeq simulates differential abundance in the data by estimating the taxa abundance distributions in the two different groups in the source data. Subsequently, new data were sampled from each of these distributions. This was done for taxa with an observed log-fold change in the source data which was at least as large as a predefined threshold (here: 0.5, 1, and 1.5). Sample sizes of 25, 50, and 75 per group were simulated. For each simulation scenario (i.e. a unique combination of sample size and log-fold change threshold), 100 data sets were simulated. These data sets had a fixed number of taxa (250) of which 10% was differentially abundant, and for the most extreme scenarios, 5% and 20% differential abundance was also considered. See Fig 1 in S1 Appendix for a visualization of the main simulation design.

In a second set of simulations, microbiome data were generated with the parametric simulation framework of [6]. These parametric simulations were based on the NB distribution, for which the parameters were estimated by means of maximum likelihood from the source data for settings A and B. For each simulated data set, parameter values of the NB distribution were sampled from the pool of estimated parameters. In particular, the mean and overdispersion parameters were sampled from the same taxon to secure any mean-dispersion relationship, and the library sizes were sampled with replacement from the observed library sizes of the source data set. We considered a FC of 1.5 and 5 in scenarios with 10% and 70% differentially abundant taxa (see S1 Appendix section ‘Negative Binomial Simulation Scenarios’ for more details). The total number of taxa was fixed at 250. Sample sizes of 25 and 75 per group were considered. For each unique combination of sample size and fold change, 100 data sets were simulated. Note that introducing differential abundance in 70% of the taxa violates the assumption that the majority of taxa are not differentially abundant for RioNorm2. Nevertheless, we included these scenarios as not all microbiome data will comply with this assumption. This analysis thus illustrates the effect of violating the assumption on the performance of the sign methods.

**Fig 2:**
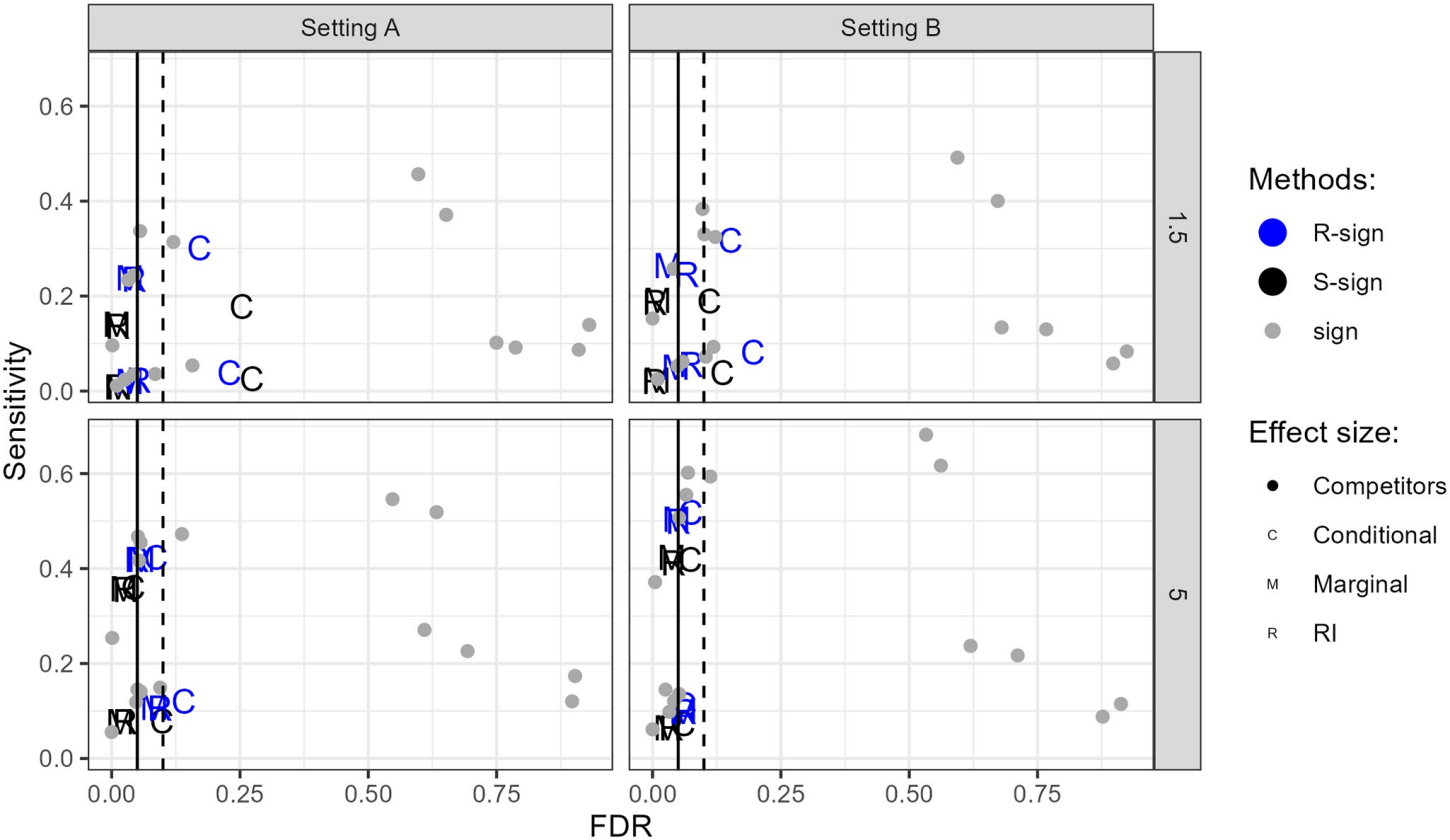
Empirical FDRs vs sensitivities of the sign methods and competitors for the various simulation scenarios with the negative Binomial distribution. The top row contains the results of scenarios with FC = 1.5, and the bottom row contains the results of scenarios with FC = 5. Left: Setting A (high sparsity) and Right: Setting B (low sparsity).

See Figs 2 and 3 in S1 Appendix for visualisations of the library size and sparsity distributions of the data for the different simulation frameworks and settings.

The RF for the sign methods were selected by RioNorm2 with default values in setting B, but in setting A we adjusted the default settings to only include taxa that were observed in at least 60% of the samples instead of 80%.

The new methods were compared to the following competitors: WMW test [36], edgeR [9], DESeq2 [10], metagenomeSeq [37], ALDEx2 [16], corncob [38], ZINQ [39] and ANCOM-BC [17]. These methods were used with their default settings.

For each simulated data set, taxa that were present in less than 5% of the samples were trimmed from the data before analysis [40]. All *p*-values were adjusted with the Benjamini and Hochberg procedure [41]. The nominal false discovery rate was set at 5% for all analyses.

### Results

Since it is not our intention to present an empirical benchmark study for comparing all methods for testing for DA, but rather to focus on how the new methods compare to the competitors, the results of the empirical FDRs and sensitivities for the various simulation scenarios and tests are summarised in only a few plots showing the sensitivity versus FDR. We prepared separate plots for settings A and B of the SPSimSeq (Fig 1) and NB (Fig 2) simulations. More detailed results are presented in S2 Appendix.

The type I error rate control was also assessed (see Figs 3, 6, 9, 12, 15, 18, 21 and 24 in S2 Appendix). We can see that the new tests control the type I error rate well at the 5% level of significance, except in the extreme scenarios of the NB simulations with 70% DA taxa and a strong compositional effect (FC = 5). In these extreme scenarios also the the competitor tests have no control over the type I error rate.

For the SPSimSeq simulations, Fig 1 shows that the new methods control the FDR quite well overall. However, the S-Sign RI method in setting A is a clear exception. Many competitors do not succeed in controlling the FDR; for example, MetagenomeSeq in Setting A has FDRs of about 75%. Some of the other competitors succeed well in the FDR control: e.g. ANCOM-BC and the WMW test. The conclusions for the competitors agree with the literature; see e.g. [6, 42, 43].

In terms of the sensitivity, the R sign methods generally perform better than the S-Sign methods. The R-sign methods show similar sensitivities as ANCOM-BC and the WMW test. Note that in discussing the sensitivity, for a fair comparison, we only considered tests with a reasonable FDR control. The performance of the sign methods also depends on the performance of RioNorm2 to select a RF without DA taxa. In Setting B, RioNorm2 had no problems selecting RFs without DA (Table 2 in S1 Table). In Setting A, RioNorm2 had more difficulties selecting RFs without DA taxa, which reflects on the reduced sensitivity of the sign methods in this setting. The impact of the misspecification of the RF was especially noticeable for the S-sign methods and less for the R-sign methods, as the R-sign methods still have competitive sensitivities while controlling the FDR.

For the parametric negative binomial simulations, Fig 2 shows that the new methods again control FDR quite well, with the exception of some of the conditional Sign methods in the small fold-chance scenarios (FC = 1.5). Among the competitors, metagenomeSeq, edgeR and ZINQ have very large FDRs; the others show rather good FDR control. However, under the extreme scenarios with 70% DA (Figs 15 and 18 in S2 Appendix), no competitor nor sign method controlled the FDR in both settings, which illustrates that all included methods have difficulties in FDR control when the majority of taxa are differentially abundant. Our results also show that in the 70% DA case, all selected reference frames contain differential abundant taxa (see Tables 3 and 4 in S1 Table). This explains the inflated FDR of the sign methods.

In terms of sensitivity, the new methods are comparable to the WMW test. The highest powers are seen for DESeq2, followed by ANCOM-BC. The differences in sensitivity become smaller as the sample size and the effect size increase. Note that DESeq2 shows here very good behavior, whereas this was not so under all the SPSimSeq simulations.

In order to ensure the generalizability of the sign methods across different microbiome studies with varying characteristics, we also applied the SPsimSeq simulation framework to an additional source dataset from a study on the coinfection of *Plasmodium vivax* and Soil-Transmitted Helminths [44]. This microbiome dataset comes from a different host (children) and was studied under different experimental conditions (co-infections versus no infection). Young children have lower gut microbiome diversity than adults. The results of this additional simulation study (see S3 Appendix) are very similar to those based on the HGM source data. However, in Setting A, ANCOM-BC is more sensitive in comparison to the results based on the HGM data. This difference in sensitivity can be explained by the difference in library size; the HGM dataset and the new source dataset have a median library size of 1977 and 57735.5, respectively.

Based on all simulations, we can conclude that overall the new methods control the FDR quite well, with a few exceptions. Among the competitors, only ANCOM-BC, ALDEx2 and the Wilcoxon rank sum test have overall good FDR control. Note that DESeq2 showed very good behaviour in the NB simulations, whereas this was not so under all the SPSimSeq simulations. The explanation is that DESeq2 makes use of the NB assumption; see also [6, 11, 45, 46]. The sensitivity of the R-Sign methods is generally higher than for the S-Sign methods, and it is comparable to the sensitivity of ANCOM-BC in realistic scenarios. However, when the data has high taxa abundances, ANCOM-BC outperforms the other methods. Among the R-sign methods, the RI method is often the best, but it may sometimes lose FDR control. We did not see important differences between the marginal and conditional R-Sign methods.

## Case study

In this case study, the application and the interpretation of the new methods are illustrated by reanalysing 16S rRNA count data of 292 fecal samples collected by [47]. The objective is to identify taxa that are differentially abundant between 120 colorectal cancer (CRC) patients and 172 healthy patients.

The analysis is focused on the genus level, and taxa that were present in less than 5% of the samples were trimmed from the data. We refer to S2 Table for some summary statistics on the data. All hypothesis tests were performed at the nominal 5% FDR level, and the original *p*-values were adjusted by the Benjamini and Hochberg procedure [41]. The selection of the RF was done by RioNorm2 [19]. The RF contains a group of 4 relatively invariant taxa, which share the same taxonomic family (*Lachnospiraceae*). This could imply that they have similar characteristics and can give a biological interpretation to the RF.

The taxon *Peptostreptococcus*, which was identified as a taxon that is strongly associated with CRC by [47], is used here to illustrate the interpretation of the effect size parameter *β*_*A*_. Additionally, we took sample specific variables into account to investigate the effect of the covariates on the effect sizes. We included the gender of the patients, and the results of a fecal immunochemical test (FIT) which is a noninvasive colorectal cancer screening test that measures hidden blood in fecal samples. We applied the S-sign and R-sign methods to test *H*_0_ : *β*_*A*_ = 0 versus *H*_1_ : *β*_*A*_ ≠ 0. Parameter estimates, standard errors (SE) and adjusted *p*-values are presented in Table 1.

**Table 1:** Estimates of the effect size parameters *β*_*A*_ for the S-sign and R-sign methods (SE and two-sided *p*-values are also reported). The S-sign estimates for *β*_*A*_ are a result from the ML parameter estimates of the logistic regression models taking the library size, gender and FIT into account. The estimates of the R-sign methods are a direct result of fitting PIMs, also taking the library size, gender and FIT into account.

Method		Estimate	S.E.	*p*-value
S-sign	Marginal	-3.135	0.671	<0.001
	Conditional	-2.841	0.698	<0.001
	RI	-2.797	0.528	<0.001
R-sign	Marginal	0.511	0.091	<0.001
	Conditional	0.433	0.113	<0.001
	RI	0.606	0.021	<0.001

First we discuss the results of the marginal S-sign method, which targets a marginal effect size which is also known as the marginal odds ratio (OR), quantified by $\text{exp}\left(\beta_{A}\right)=\frac{\text{P}\{N≼R|A=1\}/(1-\text{P}\{N≼R|A=1\})}{\text{P}\{N≼R|A=0\}/(1-\text{P}\{N≼R|A=0\})}$. The estimate of the marginal OR equals exp(−3.135) = 0.044 (*p* < 0.001), indicating a significant difference in the odds of *Peptostreptococcus* having a smaller abundance than the adjusted median of the RF, between CRC patients (A = 1) and healthy patients (A = 0). More specifically, the odds of *Peptostreptococcus* having a smaller abundance than the adjusted median of the RF for CRC patients is 96% lower than for healthy patients. Since the RF is approximately constant between groups, we could say that *Peptostreptococcus* is more abundant in CRC patients compared to healthy patients, relative to the RF. The conditional S-sign method was used to take the library size, gender and the results of FIT into account, which comes with the conditional OR $=\text{log}\;\left(\frac{\left(\frac{\text{P}\{N≼R|A=1,L=l,Gender=Gender,FIT=FIT\}}{\left(1-\text{P}\{N≼R|A=1,L=l,Gender=Gender,FIT=FIT\}\right)}\right)}{\left(\frac{\text{P}\{N≼R|A=0,L=l,Gender=Gender,FIT=FIT\}}{\left(1-\text{P}\{N≼R|A=0,L=l,Gender=Gender,FIT=FIT\}\right)}\right)}\right)$, estimated as 0.058 (*p* < 0.001). This estimated conditional OR can be interpreted as follows: *Peptostreptococcus* is more abundant in CRC patients than in healthy patients, relative to the RF, given that the library sizes, gender and FIT are constant. The RI S-sign method considers the library size, gender and FIT as auxiliary information, while still providing an estimator of the marginal effect size, but with potentially increased efficiency (lower variance) as compared to the marginal S-Sign method. The conclusion from this analysis is very similar to the conclusion from the marginal S-sign analysis.

Next we discuss the results from the R-sign methods. The effect size measure for the marginal R-sign method, also known as the marginal probabilistic index (MPI), is defined as *P*{*N*/*R* ≼ N*/R*|*A* = 0, A* = 1} = expit (*β*_*A*_). With $\text{expit}\;\left({\hat{\beta }}_{A}\right)=\frac{exp({\hat{\beta }}_{A})}{1+exp({\hat{\beta }}_{A})}=0.625$, we can conclude that an CRC patient has an estimated probability of 62.5% to have a higher abundance of *Peptostreptococcus* than a healthy patient, relative to RF. The MPI is extended to a conditional probabilistic index (CPI) which accounts for the library size, gender and FIT results of the patients, with expit (*β*_*A*_) = *P*{*N*/*R* ≼ N*/R*|*A* = 0, A* = 1, *L* = L*, *Gender* = Gender*, *FIT* = FIT*}. The CPI is estimated as 0.61, which is interpreted as follows: with an estimated probability of 61% a CRC patient has a higher abundance of *Peptostreptococcus* than a healthy patient, relative to the RF and given that the library sizes, gender and FIT coincide. The RI R-sign estimator makes use of the data of the covariates, while still targeting the MPI, but potentially having an increased efficiency. The results in Table 1 show that the RI R-sign estimator has indeed a smaller standard error than the marginal R-sign method, and using the data of the covariates has no substantial effect on the effect size estimate ${\hat{\beta }}_{A}$.

Finally, we compared the pairwise concordance of the best-behaving competitors from the simulation study (ANCOM-BC and the WMW test) with the sign methods and some competitors. The pairwise concordance was based on the number of shared discoveries from a set of top-ranked taxa specified by both methods. The results are shown in S1 and S2 Figs.

## Discussion

We have introduced two types of sign transformations for microbiome data. Both transformations make use of a data-driven set of reference taxa, but can also make use of a user-defined set of reference taxa. We have chosen for RioNorm2 as a method for for selecting the reference frame (RF), but our methods are compatible with any RF selection method. The first type of sign transformation (S-sign) basically is a 0/1 indicator for a taxon count being smaller or larger than the adjusted median count of the RF. The second type (R-sign) is also a 0/1 indicator, but it compares taxon counts between samples; these R-signs are related to rank methods. These sign transforms are naturally related to logistic regression models (S-sign) and probabilistic index models (R-sign). For both types of signs we have shown that a clever use of these models allows for (1) valid inference, (2) informative interpretation of parameters, and (3) our methods inherit the flexibility of these two classes of models (e.g. including covariates, confounders, blocking factors, …). Moreover, we have proven that these methods possess the three basic properties (scale invariance, permutation invariance, and sub-compositional coherence) required for proper compositional data analysis.

The simulation study has shown that the choice of the RF may affect the performance of the sign methods. In particular, for settings with low sparsity and dispersion (evaluated using the coefficient of variation), RioNorm2 performance was the best. As a result, the sign methods perform well, and particularly the R-sign methods have similar power compared to the best competitor. However, RioNorm2 has more difficulty in selecting RFs with no differentially abundant taxa in settings with high sparsity and dispersion, which is reflected in some power loss. Nevertheless, the R-sign methods still behave similarly to the best competitor, and the S-sign methods generally still control the FDR well. It is important to note that when the data has large taxa abundances and thus a large median library size, ANCOM-BC will outperform all other methods.

In scenarios where the majority of taxa are differentially abundant and there is a strong compositional compensation effect, RioNorm2 has problems selecting reference frames with no differential abundant taxa, resulting in an inflated FDR. From our simulation results we also conclude that none of the competitors are valid in this scenario. Recall that the sign methods can be used with any external method for reference frame identification, including prior knowledge on stable taxa. This may perhaps leave an opportunity for valid inference.

A limitation of the simulation study is our choice of only using the default settings of all methods. Optimising or varying the tuning parameters of all methods would bring us too far. Moreover, default settings are also often used in other benchmarking studies, and they are often used in practice by typical users of these methods.

An important strength of our approach is that the methods do not require strong distributional assumptions and they inherit the flexibility of regression models. In the simulation study we have empirically demonstrated that our methods succeed in controlling the nominal FDR level, while having competitive sensitivity. Although the methods have been introduced here for microbiome data analysis, they may also serve other applications that are not limited to compositional data. For example, the data and problems in differential gene expression studies (RNASeq) are very similar to those in microbiome studies, but the data are traditionally not considered compositional.

## Supporting information

S1 Fig Concordance plot of the WMW test and the sign methods. (TIF)

S2 Fig Concordance plot of the ANCOM-BC and the sign methods. (TIF)

S1 Table Reference frame description for all simulation scenarios of setting A and B with SPSimSeq and the Negative Binomial distribution. (PDF)

S2 Table Summary statistics of the data for the case study (per group). (PDF)

S1 Text Basic properties of sign-transforms. (PDF)

S2 Text Relation to the log-fold change. (PDF)

S1 Appendix Simulation study set up. This appendix provides more information on the setup of the simulations by visualizing and summarising the parameters that were taken into account. Additionally, more information on the packages(and versions) used is provided.
(PDF)

S2 Appendix Detailed results of the simulation study with HGM as source dataset. (PDF)

S3 Appendix Detailed results of simulation study with the study on the coinfection of *Plasmodium vivax* and Soil-Transmitted Helminths as source dataset. (PDF)
