# Supplementary figures and images for: Simple and flexible sign and rank-based methods for testing for differential abundance in microbiome studies
Source: PLoS One. 2023 Sep 26;18(9):e0292055. doi: 10.1371/journal.pone.0292055 (PMC10522045; doi:10.1371/journal.pone.0292055)

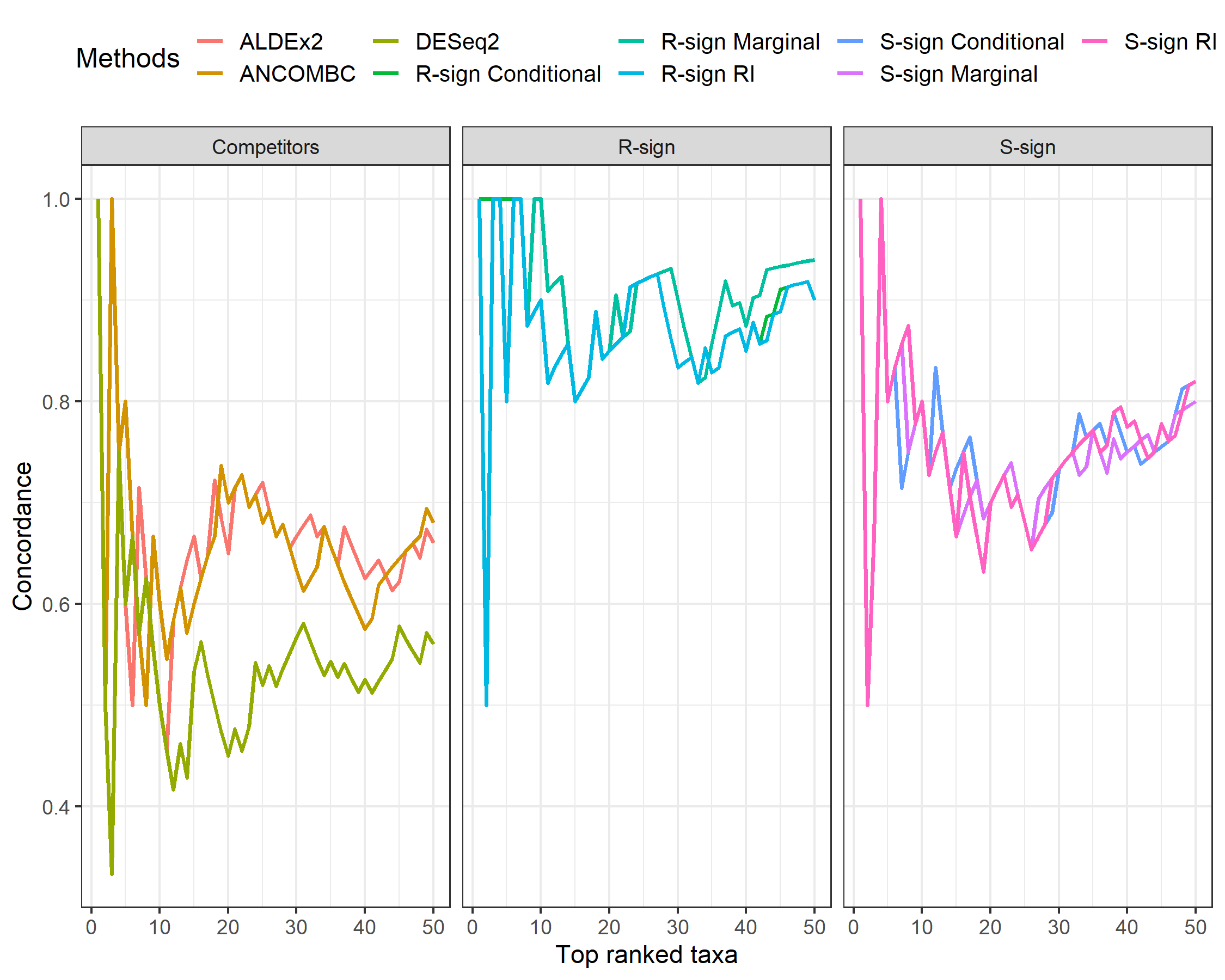

Supplement: S1 Fig — (TIF) [file pone.0292055.s001.tif]

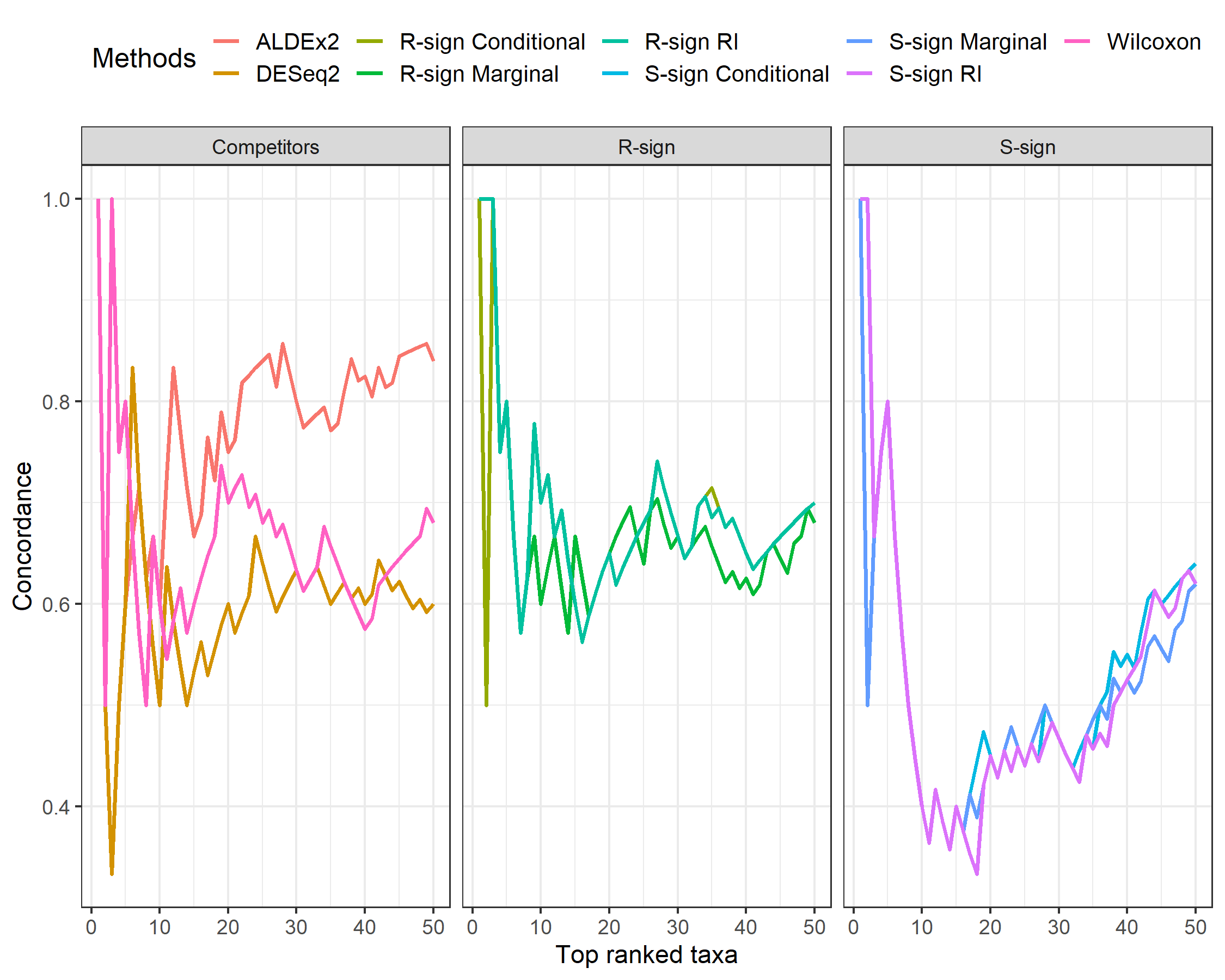

Supplement: S2 Fig — (TIF) [file pone.0292055.s002.tif]
