# Supplementary material for: Simple and flexible sign and rank-based methods for testing for differential abundance in microbiome studies
Source: PLoS One. 2023 Sep 26;18(9):e0292055. doi: 10.1371/journal.pone.0292055 (PMC10522045; doi:10.1371/journal.pone.0292055)
Supplement: S1 Table — (PDF) [file pone.0292055.s003.pdf]

S1 Tables

Table 1: Reference frame (RF) description for all simulation scenarios of setting A with SPsimseq. The first column depicts the number of simulated datasets where no RF was found. The second column shows the average length of the RF and the third column shows the number of RFs with at least one DA taxa. The last column shows the number of RFs which contains only DA taxa

Scenario	No RF	Average RF length	RF with DA taxa	Complete
1.1	3	3.217	22	0
1.2	2	3.217	19	0
1.3	4	3.094	21	0
2.1	3	3.247	15	0
2.2	5	3.221	7	0
2.3	2	3.153	12	0
3.1	5	3.158	5	0
3.2	6	3.255	17	1
3.3	7	3.194	10	2
5.1.1	2	3.276	12	0
5.3.3	6	3.128	6	0
20.1.1	7	3.097	34	2
20.3.3	4	3.063	27	8

Table 2: Reference frame (RF) description for all simulation scenarios of setting B with SPsimseq. The first column depicts the number of simulated datasets where no RF was found. The second column shows the average length of the RF and the third column shows the number of RFs with at least one DA taxa. The last column shows the number of RFs which contains only DA taxa

Scenario	No RF	Average RF length	RF with DA taxa	Complete
1.1	0	6.390	0	0
1.2	0	6.690	0	0
1.3	0	6.340	0	0
2.1	0	6.560	0	0
2.2	0	5.890	0	0
2.3	0	6.630	0	0
3.1	0	6.170	0	0
3.2	0	6.390	0	0
3.3	1	6.182	0	0
5.1.1	0	6.100	0	0
5.3.3	0	6.530	0	0
20.1.1	2	6.082	0	0
20.3.3	1	6.263	0	0

Table 3: Reference frame (RF) description for all simulation scenarios of setting A with Negative Binomial Distribution. The first column depicts the number of simulated datasets where no RF was found. The second column shows the average length of the RF and the third column shows the number of RFs with at least one DA taxa. The last column shows the number of RFs which contains only DA taxa

Scenario	No RF	Average RF length	RF with DA taxa	Complete
1.1	0	7.16	56	0
1.2	0	5.64	20	0
2.1	0	5.95	43	0
2.2	0	6.32	30	0
70.1.1	0	7.59	100	8
70.1.2	0	6.36	96	93
70.2.1	0	7.36	100	4
70.2.2	0	6.52	93	89

Table 4: Reference frame (RF) description for all simulation scenarios of setting B with Negative Binomial Distribution. The first column depicts the number of simulated datasets where no RF was found. The second column shows the average length of the RF and the third column shows the number of RFs with at least one DA taxa. The last column shows the number of RFs which contain only DA taxa

Scenario	No RF	Average RF length	RF with DA taxa	Complete
1.1	0	5.73	42	0
1.2	0	5.86	21	0
2.1	0	6.03	37	0
2.2	0	5.81	16	0
70.1.1	0	6.00	100	24
70.1.2	0	5.15	95	94
70.2.1	0	5.60	98	24
70.2.2	0	5.08	90	87
