# Supplementary material for: Simple and flexible sign and rank-based methods for testing for differential abundance in microbiome studies
Source: PLoS One. 2023 Sep 26;18(9):e0292055. doi: 10.1371/journal.pone.0292055 (PMC10522045; doi:10.1371/journal.pone.0292055)
Supplement: S2 Table — (PDF) [file pone.0292055.s004.pdf]

# S2 Table

Table 1: Summary statistics of the read counts per group (healthy patients vs. CRC patients) and of the sample variables considered in the case study. (FIT: results of a Fecal Immunochemical Test)

| | Healthy patients | CRC Patients |
|---|---|---|
| Sample size | 172 | 120 |
| Median nr. reads | 8999 | 13284 |
| Total number of reads | 2699742 | 2136259 |
| Min. nr of reads | 764 | 749 |
| Max nr of reads | 100014 | 134068 |
| Sparsity | 60% | 56% |
| Gender | 61 males<br>111 females | 68 males<br>52 females |
| FIT | mean = 8.92<br>sd = 44.70056 | mean = 789.05<br>sd = 813.8102 |
