# Supplementary material for: Simple and flexible sign and rank-based methods for testing for differential abundance in microbiome studies
Source: PLoS One. 2023 Sep 26;18(9):e0292055. doi: 10.1371/journal.pone.0292055 (PMC10522045; doi:10.1371/journal.pone.0292055)
Supplement: S1 Text — (PDF) [file pone.0292055.s005.pdf]

S1 Text - Basic properties of Sign-transforms

Aitchison (1986) proposed three principles that should hold for correct compositional data analysis: (1) scale invariance; (2) subcompositional coherence; (3) permutation invariance. In this section we will prove that our methods satisfy these properties under mild conditions.

Scale invariance:

A CoDa method is scale invariant if the data analysis results do not depend on scaling of the counts $N_{i1}, \dots, N_{im}$ within any sample i. Since the logistic regression models and PIMs for the marginal null hypothesis depend on the counts only via the S-sign or R-sign, it is sufficient to prove that these sign transforms are scale invariant.

For all $\gamma \in \mathbb{R} \setminus \{0\}$, the scaled counts γN_{it} result in a reference count that is scaled with the same factor, i.e. the reference count becomes γR_i. Hence, $\mathrm{I}\{\gamma N_{it} \preccurlyeq \gamma R_i\} = \mathrm{I}\{N_{it} \preccurlyeq R_i\}$. The same argument holds for the R-sign.

For the RI estimators and the model-based approached for the conditional null hypotheses, also the library sizes L_i are used. After scaling these become γL_i. This only affects the parameter estimates of the effect of the library size, which now become $\hat{\beta}_L/\gamma$, so that $(\hat{\beta}_L/\gamma)(\gamma L_i) = \hat{\beta}_L L_i$ and hence the final conclusions from the data analyses remain unaffected by scaling.

Permutation invariance:

Permutation invariance implies that the result of the data analysis does not depend on the order of the counts within a sample (i.e. the order of the taxa). Since our data analysis methods are performed taxon-by-taxon, and since the false discovery rate (FDR) correction procedures do not depend on the order of the p-values, the data analysis results are permutation invariant.

Subcompositional coherence:

A CoDa method is subcompositional coherent if the results of the data analysis remain the same when only a subset of the counts is used and their counts are closed in the subcomposition. This statement needs some explanation. For proving this property we make the assumption that the reference taxa are within the subcomposition.

Let $(N_{i1}, \ldots, N_{im_s})$ denote the counts in the subcomposition. Note that we selected the first m_s taxa, but because of the permutation invariance, this may represent any m_s taxa out of the m. The original definition of subcompositional coherence refers to "closing the subcomposition", which is dividing the counts of the subcomposition within a sample by its sum, $L_{is} = \sum_{t=1}^{m_s} N_{it}$. Since we did not even close the original counts (this would be an example of scaling), we ignore this part of the definition and rephrase subcompositional coherence as the property that the data analysis results are not altered by only considering a subcomposition. Our methods are applied taxon-by-taxon and the counts are not closed prior to the data analysis, but they are divided by the median-adjusted count in the RF. The latter is assumed to be part of the subcomposition and hence this operation is not affected. On the other hand, we need to think about what to do with the library sizes L_i. In the analysis of the complete count vector, they are the sums of the counts of all taxa. When considering only a subcomposition, we may either still use the L_i, or decide to use the L_{is} which only make use of the counts in the subcomposition.

If we continue to use L_i in the data analysis, the taxon-wise results remain unaltered in the subcompositional data analysis. Such analysis makes sense, as L_i keeps its original interpretation as a technical artefact that comes from the sequencing technology. Otherwise, if we use L_{is} instead, the results of the logistic regression models and PIMs will be different and depend on the subcomposition selected. We therefore suggest to always use L_i in the statistical models, even for subcompositions.

The final step of the data analysis is the adjustment of the p-values for controlling the nominal FDR level. Such procedures, however, generally depend on the number of p-values and on the empirical distribution of the observed p-values. Thus, when only a subcomposition of m_s taxa is consdered, only m_s p-values enter the FDR correction procedure, eventually resulting in different

results as compared to the data analyses based on all taxa.

Under the assumption that the reference taxa are included in the subcomposition, we conclude that our methods are subcompositional coherent in terms of effect size estimates and raw p-values. The FDR correction step will be affected, but this does not necessarily result in contradictory conclusions. Also note that working an a smaller number of taxa (subcomposition) may increase the sensitivity of the testing procedure.

References

Aitchison, J. (1986). *The Statistical Analysis of Compositional Data.* Chapman and Hall.
