# Supplementary material for: Simple and flexible sign and rank-based methods for testing for differential abundance in microbiome studies
Source: PLoS One. 2023 Sep 26;18(9):e0292055. doi: 10.1371/journal.pone.0292055 (PMC10522045; doi:10.1371/journal.pone.0292055)
Supplement: S2 Text — (PDF) [file pone.0292055.s006.pdf]

S2 Text - Relation to the Log Fold Change

The probabilities involved in the null hypotheses also serve as effect sizes. In this section we illustrate their interpretation by showing their relationship to the log fold change (LFC), which is a more conventional effect size. Since the relationship depends on the distribution of counts we will have to make a distributional assumption. Although we have argued that the NB distribution is often not appropriate, we will rely on this assumption for it still is a popular distribution for microbiome data. We limit our attention to the marginal probabilities.

Without loss of generality we will consider the counts of the reference frame as constants; this may be approximately true if the number of reference taxa (m_R) is large. The relationships described in this section only serve as an illustration to gain a better understanding of the probabilistic effect sizes.

The probabilities involved in the marginal-S null hypothesis may be combined into the odds ratio (OR)

$$OR = \frac{\mathrm{P}\left\{N \preccurlyeq R \mid A=1\right\} / \left(1 - \mathrm{P}\left\{N \preccurlyeq R \mid A=1\right\}\right)}{\mathrm{P}\left\{N \preccurlyeq R \mid A=0\right\} / \left(1 - \mathrm{P}\left\{N \preccurlyeq R \mid A=0\right\}\right)}.$$

Under the null hypothesis, we get OR=1. The probabilities $\mathrm{P}\left\{N \preccurlyeq R \mid A=a\right\}$ can be computed from the NB distributions of N in the $a=0$ and $a=1$ groups. With $F_{\mathrm{NB}}(x; m(a), l)$ the distribution of a NB distribution with mean $m(a)$ and overdispersion parameter l, and with $f_{\mathrm{NB}}(x; m(a), l)$ the corresponding density function, we find $\mathrm{P}\left\{N \preccurlyeq R \mid A=a\right\} = F_{\mathrm{NB}}(R-1; m(a), l) + 0.5 f_{\mathrm{NB}}(R; m(a), l)$. Here we set $m(0)=1$ and $m(1)=\exp(\gamma)$, so that γ is the LFC. Figure 1 shows the relationship between the LFC and the OR for $R=1$ and $R=10$ and for $l=2,5,10$.

In a similar fashion the probabilities involved in the marginal-R null hypothesis can be computed. Since we considered the reference counts to be constant, we have

$$\mathrm{P}\left\{\frac{N}{R} \preccurlyeq \frac{N^*}{R^*} \mid A=0, A^*=1\right\} = \mathrm{P}\left\{N \preccurlyeq N^* \mid A=0, A^*=1\right\}$$
$$= \sum_{x=0}^{+\infty} F_{\mathrm{NB}}(x; m(0), l) f_{\mathrm{NB}}(x; m(1), l) - \frac{1}{2}\sum_{x=0}^{+\infty} f_{\mathrm{NB}}(x; m(0), l) f_{\mathrm{NB}}(x; m(1), l).$$

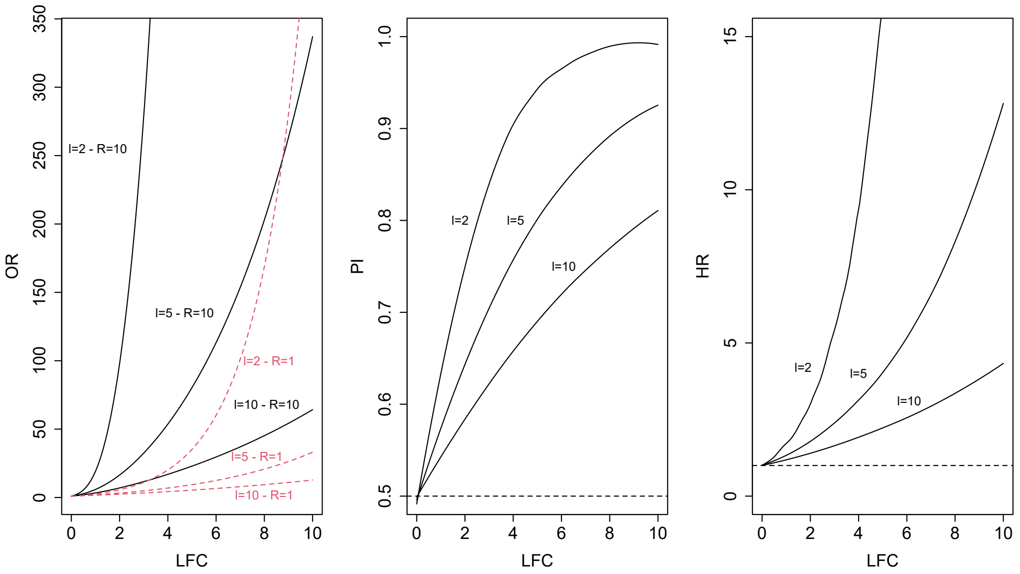

Figure 1: The relationship between the log fold change (LFC) and the odds ratio transform of the S-sign effect size (left), the probabilistic index (PI) for the R-sign effect size (middle) and the corresponding hazard ratio (right). The lines corresponds to values of overdispersion parameter l and the count of the reference frame (only for the OR).

Effect sizes of this form are known as probabilistic indices (PI) (Thas et al., 2012). De Neve and Gerds (2020), among others, have shown that this expression is related to the hazard ratio (HR) in Cox proportional hazard models. In particular, if N and N^* were time-to-events, the HR is given by $\mathrm{HR} = (\mathrm{P}\left\{N \preccurlyeq N^* \mid A = 0, A^* = 1\right\}) \,/\, (1 - \mathrm{P}\left\{N \preccurlyeq N^* \mid A = 0, A^* = 1\right\})$. Figure 1 also shows the relationships between the LFC and the PI and the HR.

References

De Neve, J. and Gerds, T. A. (2020). On the interpretation of the hazard ratio in cox regression. *Biometrical Journal* **62**, 742–750.

Thas, O., De Neve, J., Clement, L., and Ottoy, J. (2012). Probabilistic index models. *Journal of the Royal Statistical Society, Series B* **74**, *1–29*
