# Supplementary material for: Simple and flexible sign and rank-based methods for testing for differential abundance in microbiome studies
Source: PLoS One. 2023 Sep 26;18(9):e0292055. doi: 10.1371/journal.pone.0292055 (PMC10522045; doi:10.1371/journal.pone.0292055)
Supplement: S1 Appendix — This appendix provides more information on the setup of the simulations by visualizing and summarising the parameters that were taken into account. Additionally, more information on the packages(and versions) used is provided. (PDF) [file pone.0292055.s007.pdf]

S1 Appendix - Simulation study set up

Table 1: R-packages and version numbers used in the simulation study.

R-package	Version number
boot	1.3-28
ggplot2	3.3.5
logistf	1.24.1
micorbiome	1.12.0
phyloseq	1.34.0
pim	2.0.2
RioNorm2	0.1
SPsimSeq	1.0.0
SingleCellExperiment	1.12.0

Table 2: Competitors included in the simulation study.

Competitor	Distribution	Normalization	CoDa	Package(version)
ALDEx2	Dirichlet-multinomial	CLR	Yes	ALDEx2(1.22.0)
ANCOM-BC	Non-parametric	ALR	Yes	ANCOMBC(1.0.5)
corncob	Beta Binomial	None	No	corncob(0.2.0)
DESeq2	Negative Binomial	Relative log expression	No	DESeq2(1.30.1)
edgeR	Negative Binomial	Trimmed mean of m-values	No	edgeR(3.32.1)
metagenomeSeq	Zero-inflated (log-)Normal	Cumulative sum scaling	No	metagenomeSeq(1.32.0)
Wilcoxon	Non-parametric	None	No	stats(3.6.2)
ZINQ	Non-parametric	rarefaction	No	ZINQ(1.0)

SPsimSeq simulation scenarios

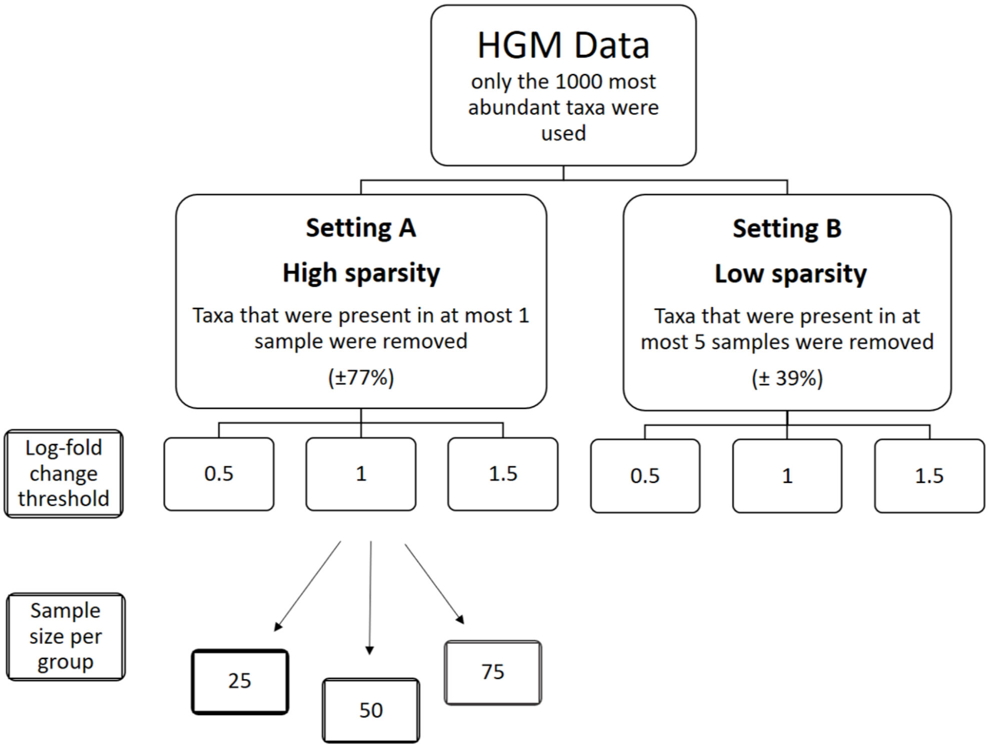

Figure 1: Main simulation design. Each unique combination of log-fold change threshold and sample size is considered as simulation scenario for SPSimSeq.

Table 3: Simulation scenarios SPsimseq. One hundred data sets per scenario were simulated with the following characteristics. The number of taxa in total was fixed on 250.

Scenario	Sample size per group	Log-fold change threshold	DA
1.1	25	0.5	10%
1.2	50	0.5	10%
1.3	75	0.5	10%
2.1	25	1	10%
2.2	50	1	10%
2.3	75	1	10%
3.1	25	1.5	10%
3.2	50	1.5	10%
3.3	75	1.5	10%
5.1.1	25	0.5	5%
5.3.3	75	1.5	5%
20.1.1	25	0.5	20%
20.3.3	75	1.5	20%
70.1.1	25	0.5	70%
70.3.3	75	1.5	70%

Negative Binomial simulation scenarios

Table 4: Simulation scenarios NB-framework. One hundred data sets per scenario were simulated with the following characteristics.

Scenario	Sample size per group	Fold change	DA
1.1	25	1.5	10%
1.2	75	1.5	10%
2.1	25	5	10%
2.2	75	5	10%
70.1.1	25	1.5	70%
70.1.2	75	1.5	70%
70.2.1	25	5	70%
70.2.2	75	5	70%

This simulation framework was based on work from Hawinkel and colleagues (2019). In the scenarios with 10% differential abundance, the fold change was introduced by using a fraction ($\frac{1}{FC+1}$) of the 10% differentially abundant taxa, of which the relative abundances sum to a. The parameters for mean relative abundances of this fraction were multiplied by the fold change factor (1.5 and 3). The remaining fraction ($\frac{FC}{FC+1}$) of the 10% differentially abundant taxa, of which the relative abundances sum to b, were multiplied by $\frac{a}{b}(1-FC)+1$. This made sure that the sum of all mean relative abundances was equal to one again, and the mean relative abundances of the remaining taxa were not affected. Alternatively, for the scenarios with 70% differential abundance, the parameter for the mean relative abundances of 70% differentially abundant taxa (that were randomly selected), was multiplied by a fold change in one group. Subsequently, the parameters of all taxa were changed to make the sum of the mean relative abundances of all taxa equal to 1. This introduces DA in the remaining 30% of the taxa.

* Hawinkel, S., Mattiello, F., Bijnens, L., Thas, O. (2019). A broken promise: microbiome differential abundance methods do not control the false discovery rate. *Briefings in bioinformatics*, 20(1), 210-221

Library sizes of source data and simulated data

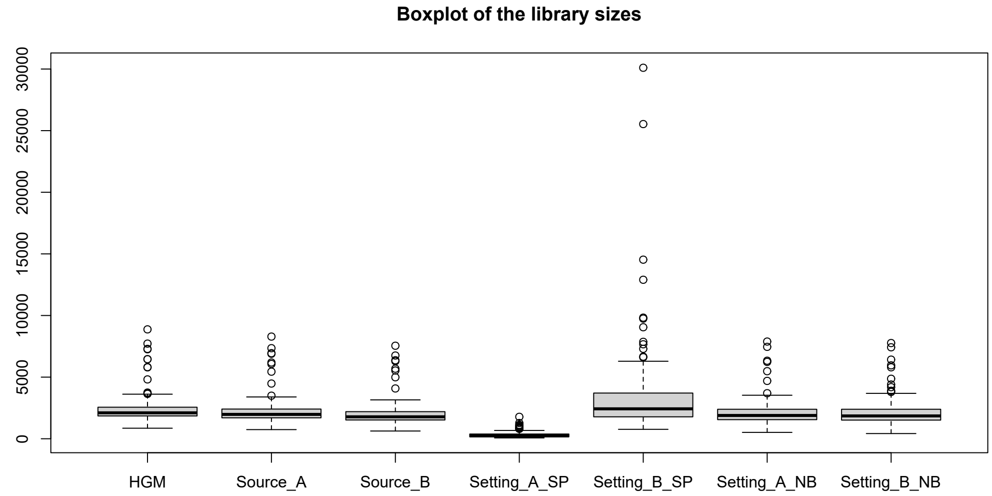

Figure 2: The distribution of the library sizes of the source data (HGM), the pre-processed data (Source_A and Source_B) and for simulated data of SPsimSeq (setting_A_SP, setting_B_SP) and the negative binomial distribution (setting_A_NB, setting_B_NB). The distribution of the library sizes for simulated data is based on a random selected dataset.

Fraction of zero counts per sample of source data and simulated data

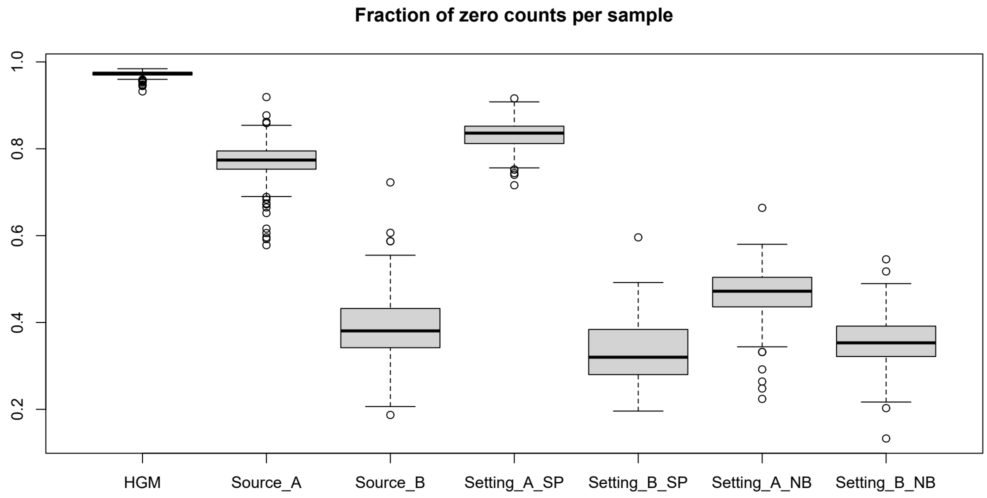

Figure 3: Fraction of zero counts per sample of the source data (HGM), the pre-processed data (Source_A and Source_B) and for simulated data of SP-simSeq (setting_A_SP, setting_B_SP) and the negative binomial distribution (setting_A_NB, setting_B_NB). The fraction of zero counts per sample for simulated data is based on a random selected dataset.

Diagnostic plots

To compare the simulated SPsimSeq data with the source data the following comparison metrics were used:

- the distribution of mean, variance and coefficients of variation (CV) of taxa abundance levels,
- the relationship between the mean and variance and the mean and CV of taxa abundance levels,
- the distribution of the fraction of zero counts per taxon and its relationship with the mean abundance level,
- the distribution of the pairwise correlation coefficients between the taxa and samples.

Setting A

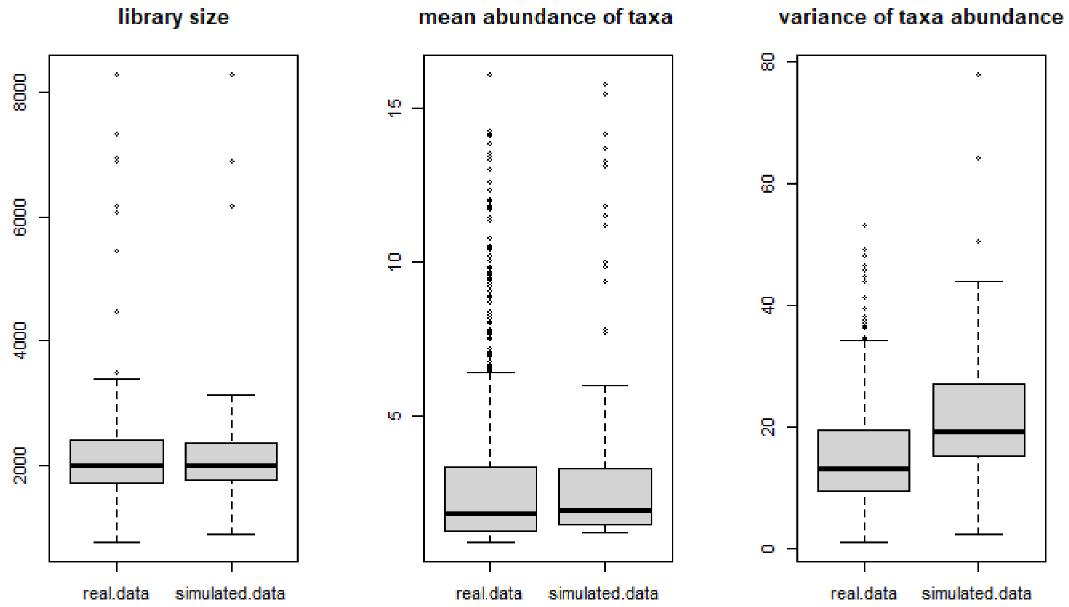

Figure 4: The distributions of the library size, mean and variance of taxa abundance from the pre-processed data (Source_A) and for simulated data of SPsimSeq (scenario 2.2).

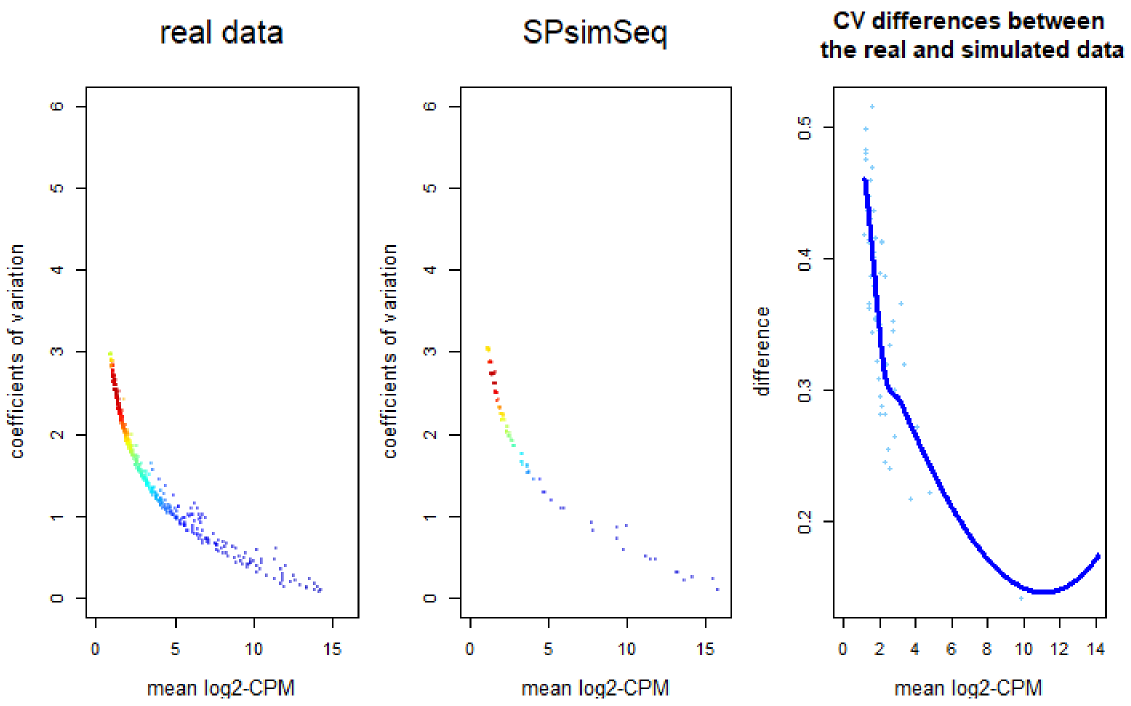

Figure 5: The relationship between the mean and coefficient of variation (CV) of taxa abundance levels (log-CPM) from the pre-processed data (Source_A) and for simulated data of SPsimSeq (scenario 2.2).

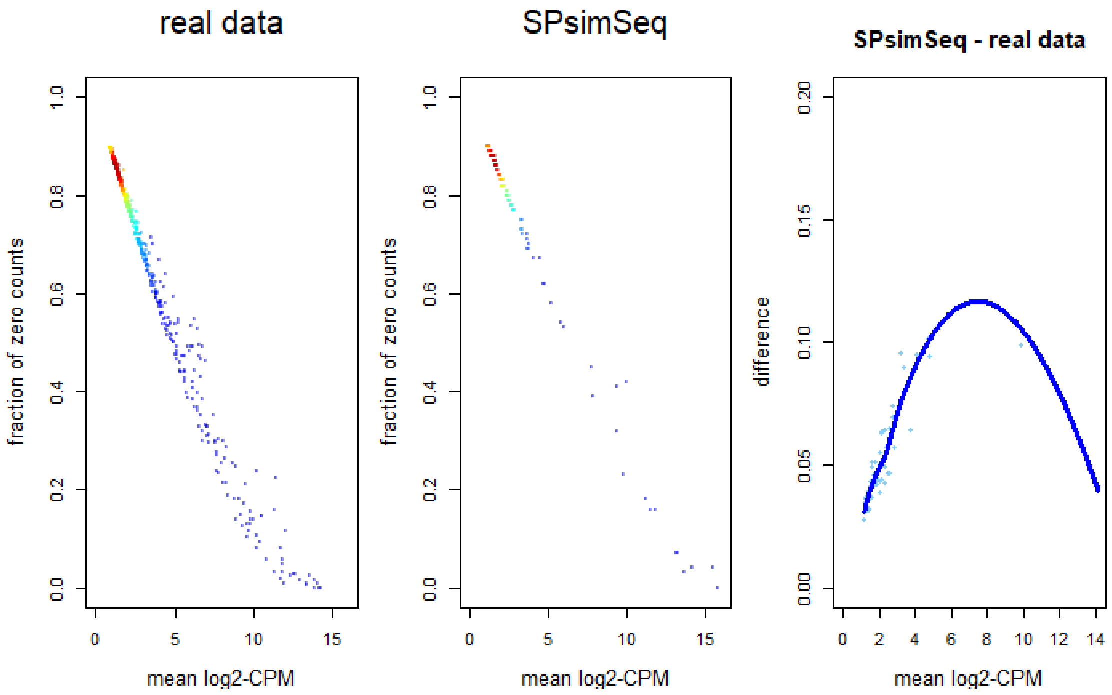

Figure 6: The fraction of zero counts per gene as a function of the mean taxa abundance levels (log-CPM) from the pre-processed data (Source_A) and for simulated data of SPsimSeq (scenario 2.2).

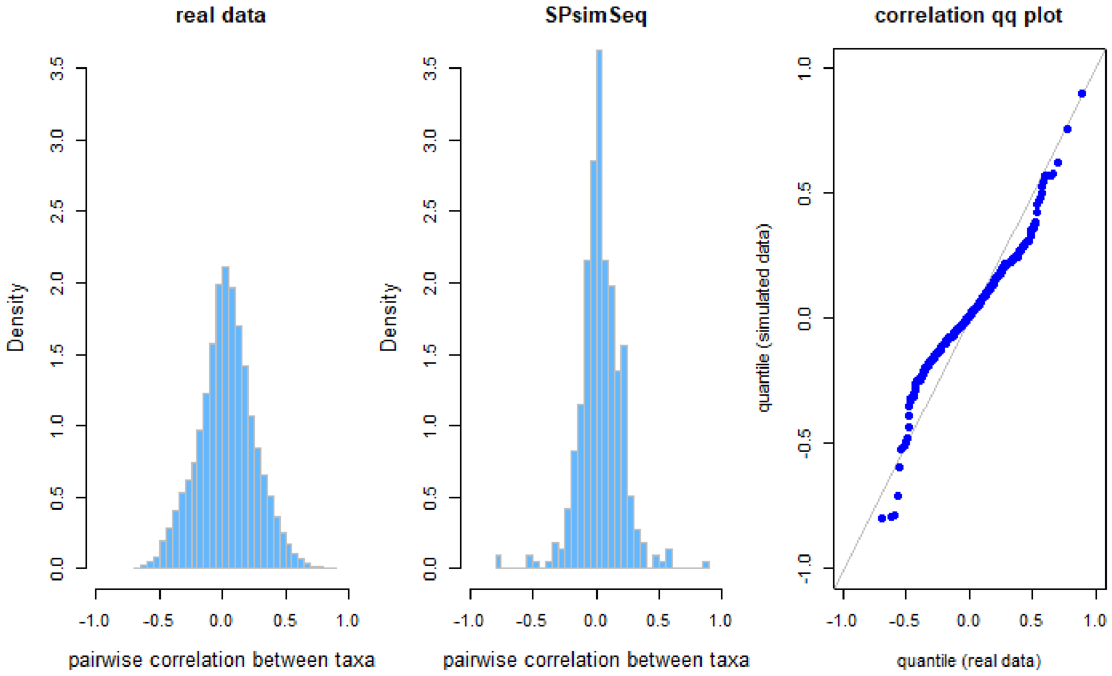

Figure 7: The distributions of the pairwise Pearson correlation-coefficients between taxa from the pre-processed data (Source_A) and for simulated data of SPsimSeq (scenario 2.2).

Setting B

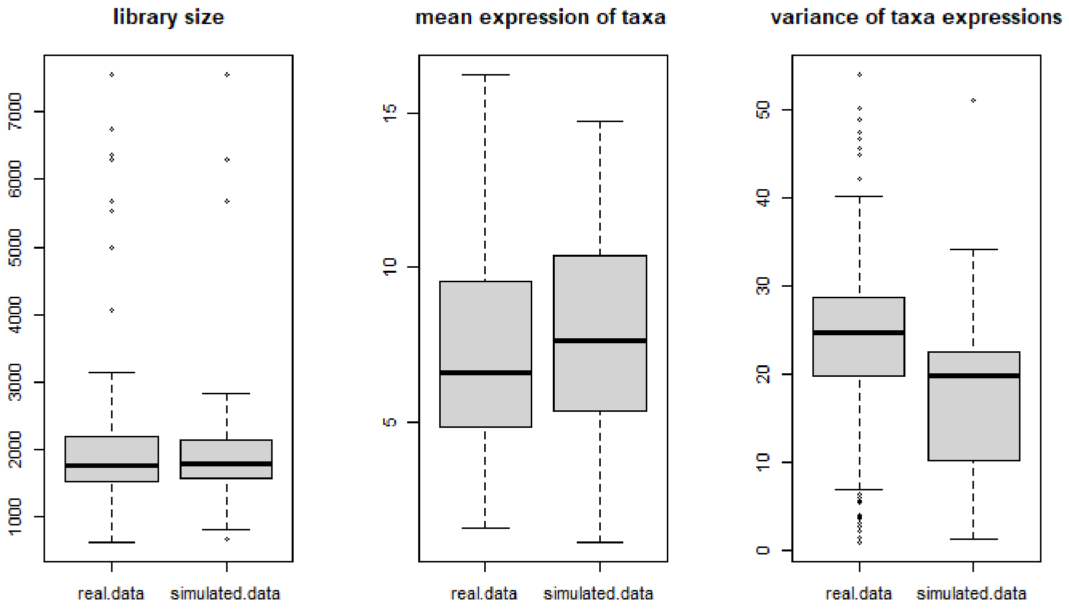

Figure 8: The distributions of the library size, mean and variance of taxa abundance from the pre-processed data (Source_B) and for simulated data of SPsimSeq (scenario 2.2).

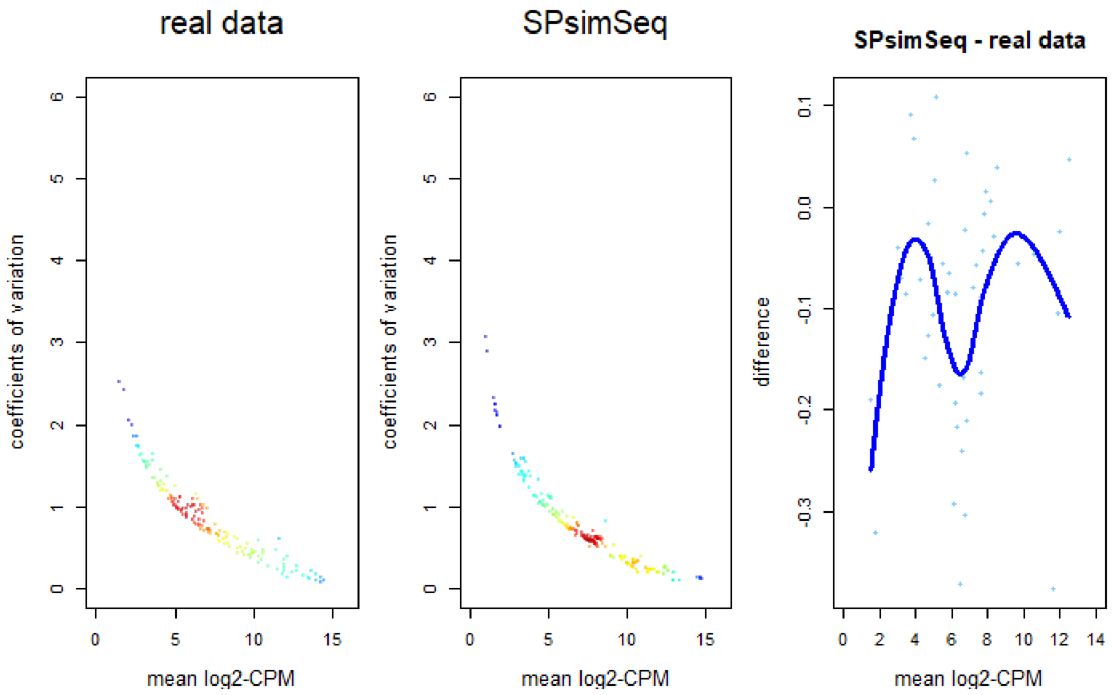

Figure 9: The relationship between the mean and coefficient of variation (CV) of taxa abundance levels (log-CPM) from the pre-processed data (Source_B) and for simulated data of SPsimSeq (scenario 2.2).

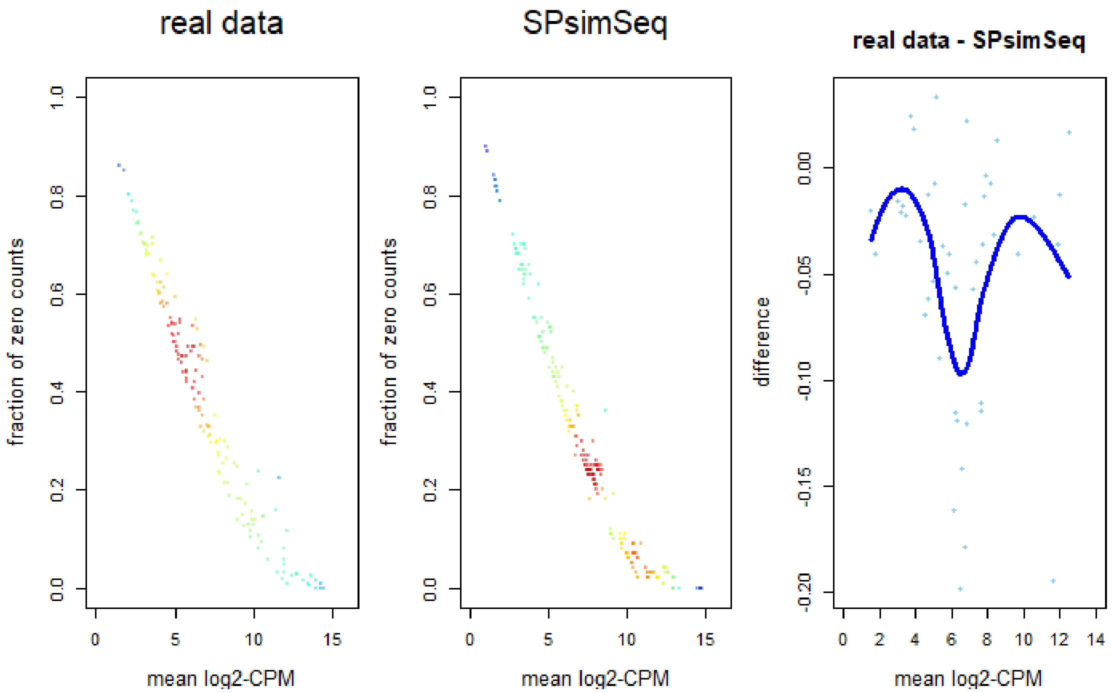

Figure 10: The fraction of zero counts per gene as a function of the mean taxa abundance levels (log-CPM) from the pre-processed data (Source_B) and for simulated data of SPsimSeq (scenario 2.2).

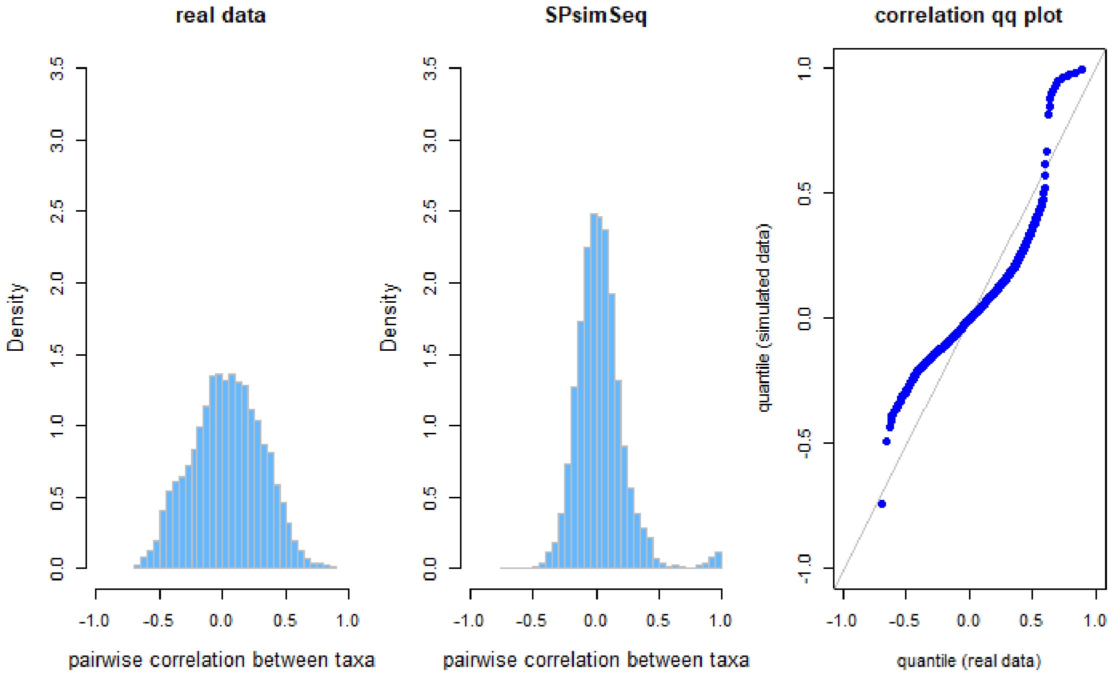

Figure 11: The distributions of the pairwise Pearson correlation-coefficients between taxa from the pre-processed data (Source_B) and for simulated data of SPsimSeq (scenario 2.2).
