# Supplementary material for: Simple and flexible sign and rank-based methods for testing for differential abundance in microbiome studies
Source: PLoS One. 2023 Sep 26;18(9):e0292055. doi: 10.1371/journal.pone.0292055 (PMC10522045; doi:10.1371/journal.pone.0292055)
Supplement: S2 Appendix — (PDF) [file pone.0292055.s008.pdf]

S2 Appendix - Detailed results of simulation study

List of Figures

1 Empirical sensitivities for the SPSimSeq simulations for setting A (high sparsity) for the new methods (left) and the competitors (right) with increasing sample size (25,50,75) and increasing log-fold change (0.5,1,1.5) and 10% DA. 5

2 Empirical FDRs for the SPSimSeq simulations for setting A (high sparsity) for the new methods (left) and the competitors (right) with increasing sample size (25,50,75) and increasing log-fold change (0.5,1,1.5) and 10% DA. The nominal FDR was set on 0.05 (solid line). 6

3 Empirical Type 1 error rate for the SPSimSeq simulations for setting A (high sparsity) for the new methods (right) and competitors (left) with increasing sample size (25,50,75),increasing log-fold change (0.5,1,1.5) and 10% DA. 7

4 Empirical sensitivities for the SPSimSeq simulations for setting B (low sparsity) for the new methods (left) and the competitors (right) with increasing sample size (25,50,75) and increasing log-fold change (0.5,1,1.5) and 10% DA. 8

5 Empirical FDRs for the SPSimSeq simulations for setting B (low sparsity) for the new methods (left) and the competitors (right) with increasing sample size (25,50,75) and increasing log-fold change (0.5,1,1.5) and 10% DA. The nominal FDR was set on 0.05 (solid line). 9

6 Empirical Type 1 error rate for the SPSimSeq simulations for setting B (low sparsity) for the new methods (right) and competitors (left) with increasing sample size (25,50,75), increasing log-fold change (0.5,1,1.5) and 10% DA. 10

7 Empirical sensitivities for the NB simulations for setting A (high sparsity) for the new methods (right) and competitors (left) with increasing sample size (25,75), increasing fold change (1.5, 5)and 10% DA. 11

8 Empirical FDR for the NB simulations for setting A (high sparsity) for the new methods (right) and competitors (left) with increasing sample size (25,75), increasing fold change (1.5, 5) and 10% DA. The nominal FDR was set on 0.05 (solid line). 12

9 Empirical Typer I error rate for the NB simulations for setting A (high sparsity) for the new methods (right) and competitors (left) with increasing sample size (25,75), increasing fold change (1.5, 5) and 10% DA. 13

10 Empirical sensitivities for the NB simulations for setting B (low sparsity) for the new methods (right) and competitors (left) with increasing sample size (25,75), increasing fold change (1.5, 5) and 10% DA. 14

11 Empirical FDR for the NB simulations for setting B (low sparsity) for the new methods (right) and competitors (left) with increasing sample size (25,75), increasing fold change (1.5, 5) and 10% DA. The nominal FDR was set on 0.05 (solid line). . 15

12 Empirical Type I error rate for the NB simulations for setting B (low sparsity) for the new methods (right) and competitors (left) with increasing sample size (25,75), increasing fold change (1.5, 5) and 10% DA. 16

13 Empirical sensitivities for the NB simulations for setting A (high sparsity) for the new methods (right) and competitors (left) with increasing sample size (25,75), increasing fold change (1.5, 5) and 70% DA. 17

14 Empirical FDR for the NB simulations for setting A (high sparsity) for the new methods (right) and competitors (left) with increasing sample size (25,75), increasing fold change (1.5, 5) and 70% DA. The nominal FDR was set at 0.05 (solid line). 18

15 Empirical Type I error rate for the NB simulations for setting A (high sparsity) for the new methods (right) and competitors (left) with increasing sample size (25,75), increasing fold change (1.5, 5) and 70% DA. 19

16 Empirical sensitivities for the NB simulations for setting B (low sparsity) for the new methods (right) and competitors (left) with increasing sample size (25,75), increasing fold change (1.5, 5) and 70% DA. 20

17 Empirical FDR for the NB simulations for setting B (low sparsity) for the new methods (right) and competitors (left) with increasing sample size (25,75), increasing fold change (1.5, 5) and 70% DA. The nominal FDR was set at 0.05 (solid line). . 21

18 Empirical Type I error rate for the NB simulations for setting B (low sparsity) for the new methods (right) and competitors (left) with increasing sample size (25,75), increasing fold change (1.5, 5) and 70% DA. 22

19 Empirical sensitivities for the SPSimSeq simulations for setting A (high sparsity) scenario 1.1 and 3.3 for the new methods (right) and competitors (left) with increasing differential abundance rate (5%, 10% and 20%). 23

20 Empirical FDRs for the SPSimSeq simulations for setting A (high sparsity) scenario 1.1 and 3.3 for the new methods (right) and competitors (left) with increasing differential abundance rate (5%, 10% and 20%). 24

21 Empirical Type I error rate for the SPSimSeq simulations for setting A (high sparsity) scenario 1.1 and 3.3 for the new methods (right) and competitors (left) with increasing differential abundance rate (5%, 10% and 20%). 25

22 Empirical sensitivities for the SPSimSeq simulations for setting B (low sparsity) scenario 1.1 and 3.3 for the new methods (right) and competitors (left) with increasing differential abundance rate (5%, 10% and 20%). 26

23 Empirical FDRs for the SPSimSeq simulations for setting B (low sparsity) scenario 1.1 and 3.3 for the new methods (right) and competitors (left) with increasing differential abundance rate (5%, 10% and 20%). 27

24 Empirical Type I error rate for the SPSimSeq simulations for setting B (low sparsity) scenario 1.1 and 3.3 for the new methods (right) and competitors (left) with increasing differential abundance rate (5%, 10% and 20%). 28

List of Tables

1 Empirical FDR for the SPSimSeq simulations in setting A and B, for the three thresholds of the log-fold change (0.5, 1 and 1.5), with a sample size of 25 per group and 10% DA. The nominal FDR was set to 5%. Empirical FDRs larger than 10% are printed in bold face. 29

2 Empirical FDR for the SPSimSeq simulations in setting A and B, for the three thresholds of the log-fold change (0.5, 1 and 1.5), with a sample size of 50 per group and 10% DA. The nominal FDR was set to 5%. Empirical FDRs larger than 10% are printed in bold face. 30

3 Empirical FDR for the SPSimSeq simulations in setting A and B, for the three thresholds of the log-fold change (0.5, 1 and 1.5), with a sample size of 75 per group and 10% DA. The nominal FDR was set to 5%. Empirical FDRs larger than 10% are printed in bold face. 31

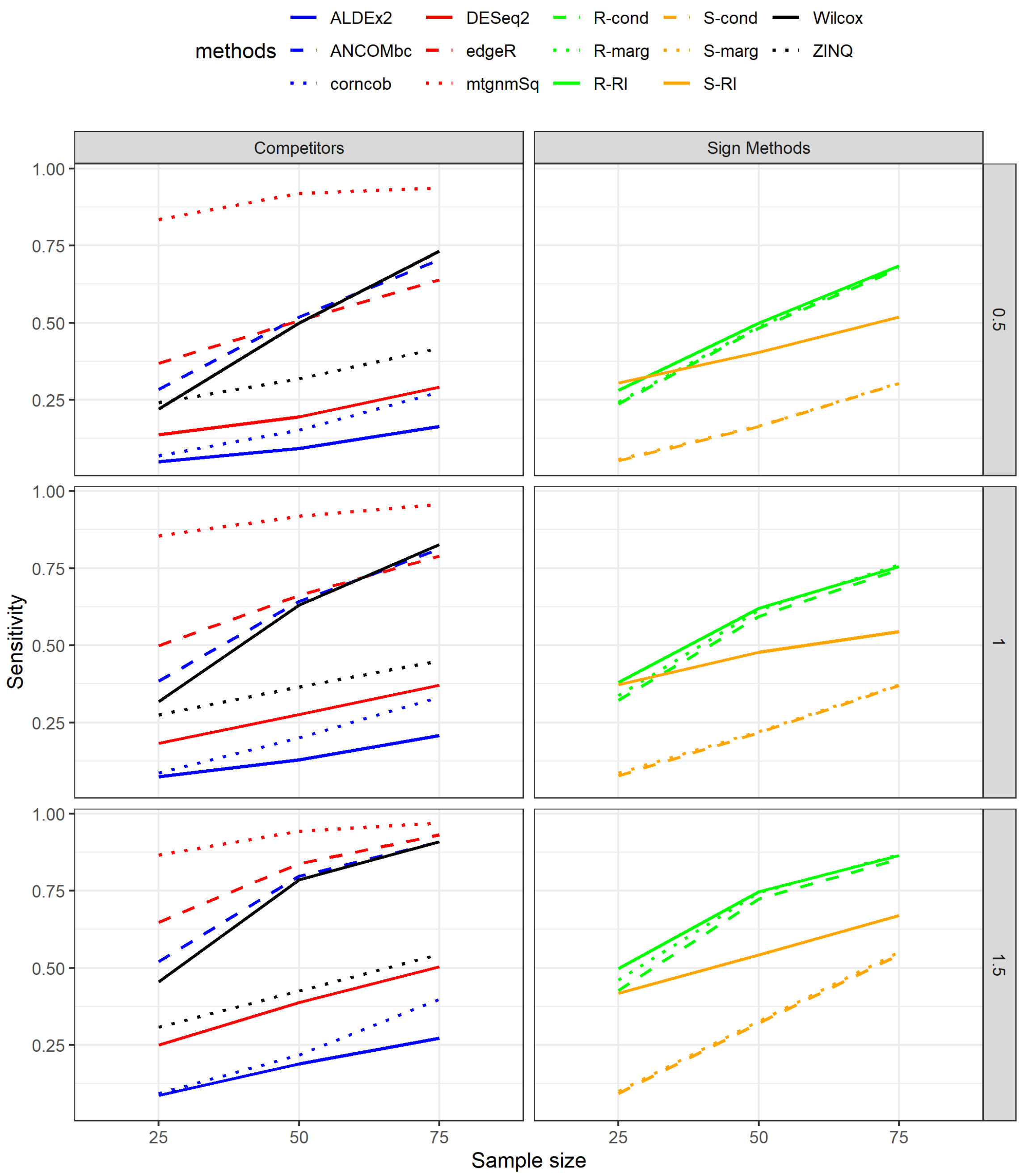

Figure 1: Empirical sensitivities for the SPSimSeq simulations for setting A (high sparsity) for the new methods (left) and the competitors (right) with increasing sample size (25,50,75) and increasing log-fold change (0.5,1,1.5) and 10% DA.

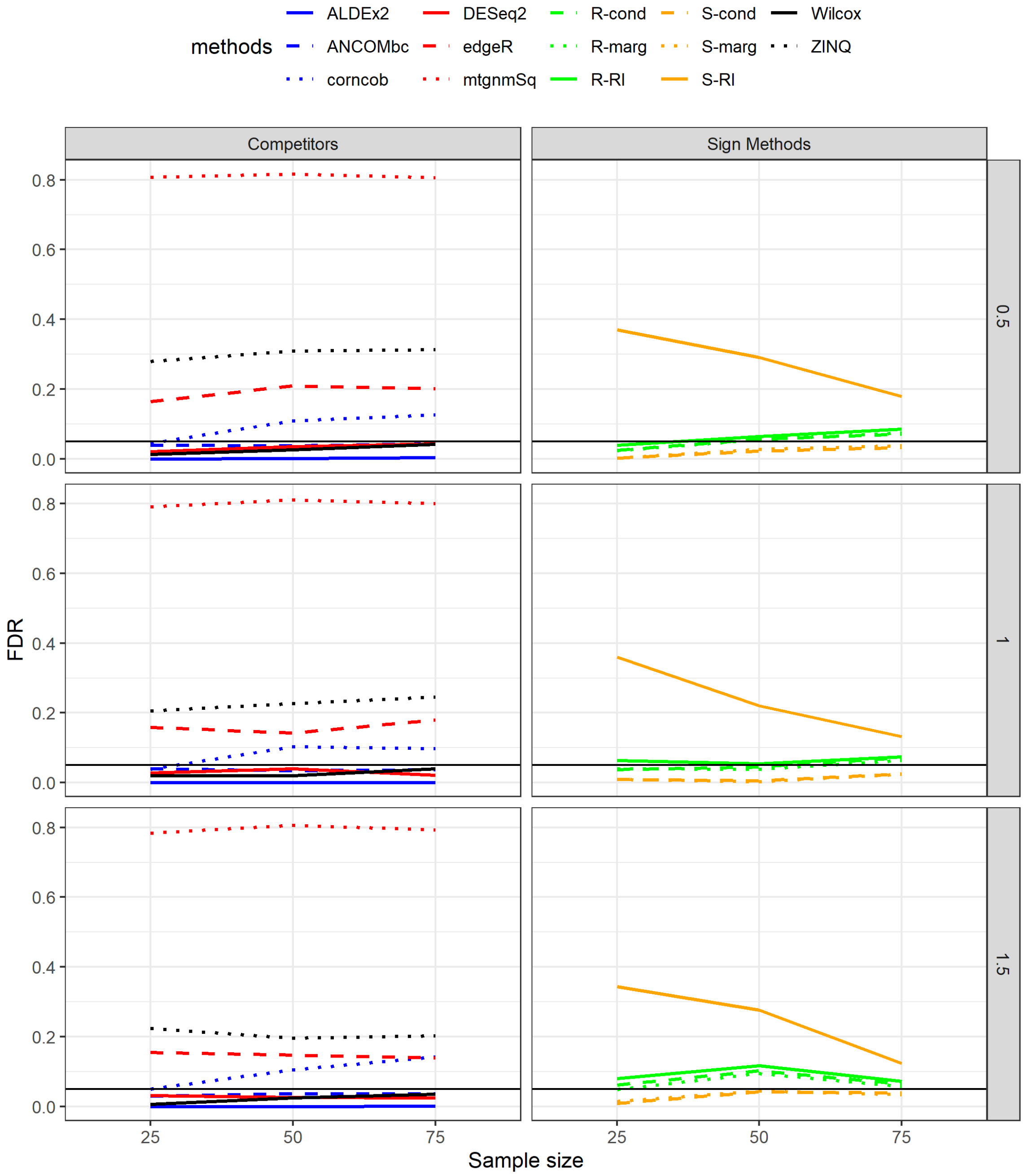

Figure 2: Empirical FDRs for the SPSimSeq simulations for setting A (high sparsity) for the new methods (left) and the competitors (right) with increasing sample size (25,50,75) and increasing log-fold change (0.5,1,1.5) and 10% DA. The nominal FDR was set on 0.05 (solid line).

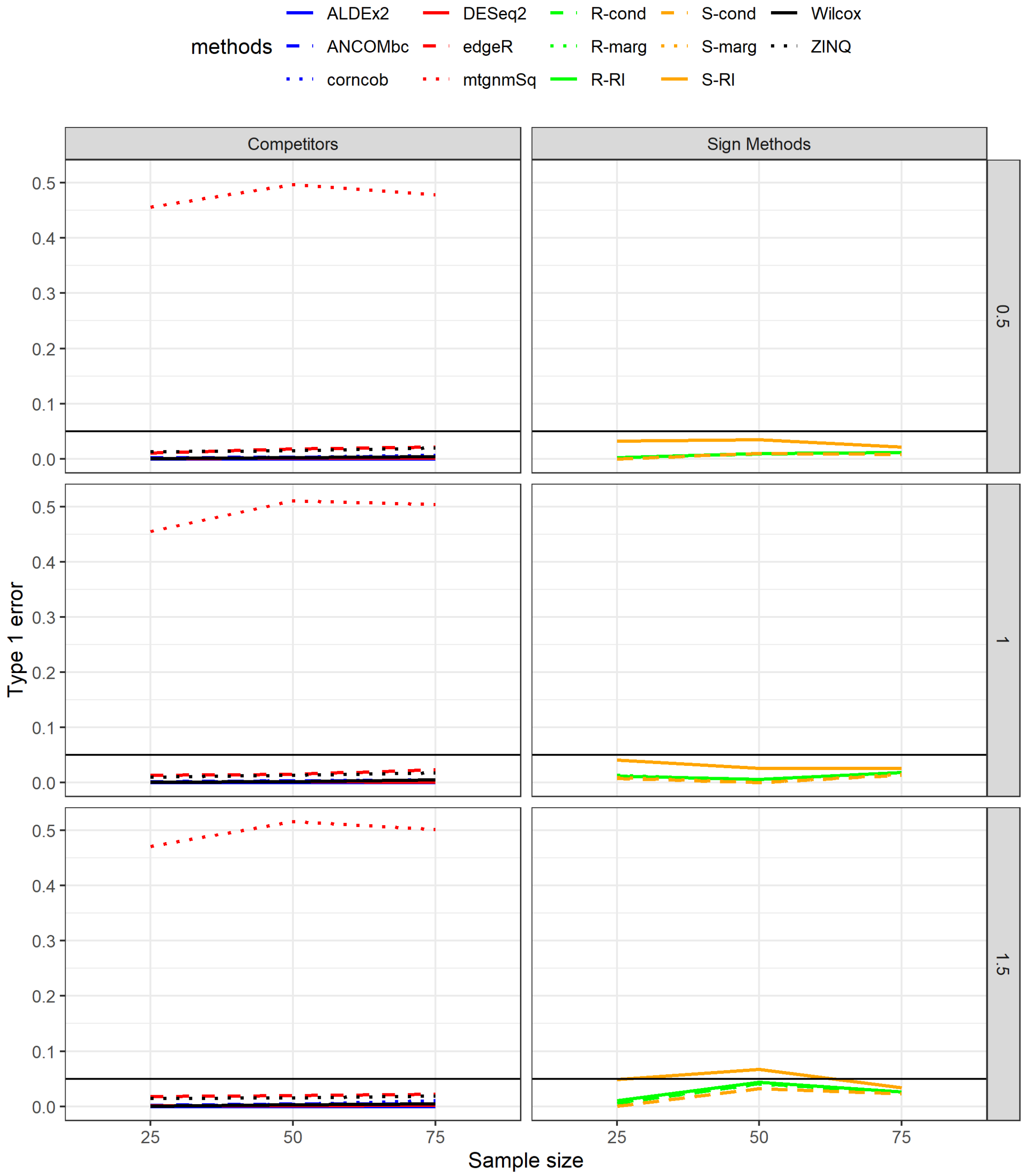

Figure 3: Empirical Type 1 error rate for the SPSimSeq simulations for setting A (high sparsity) for the new methods (right) and competitors (left) with increasing sample size (25,50,75),increasing log-fold change (0.5,1,1.5) and 10% DA.

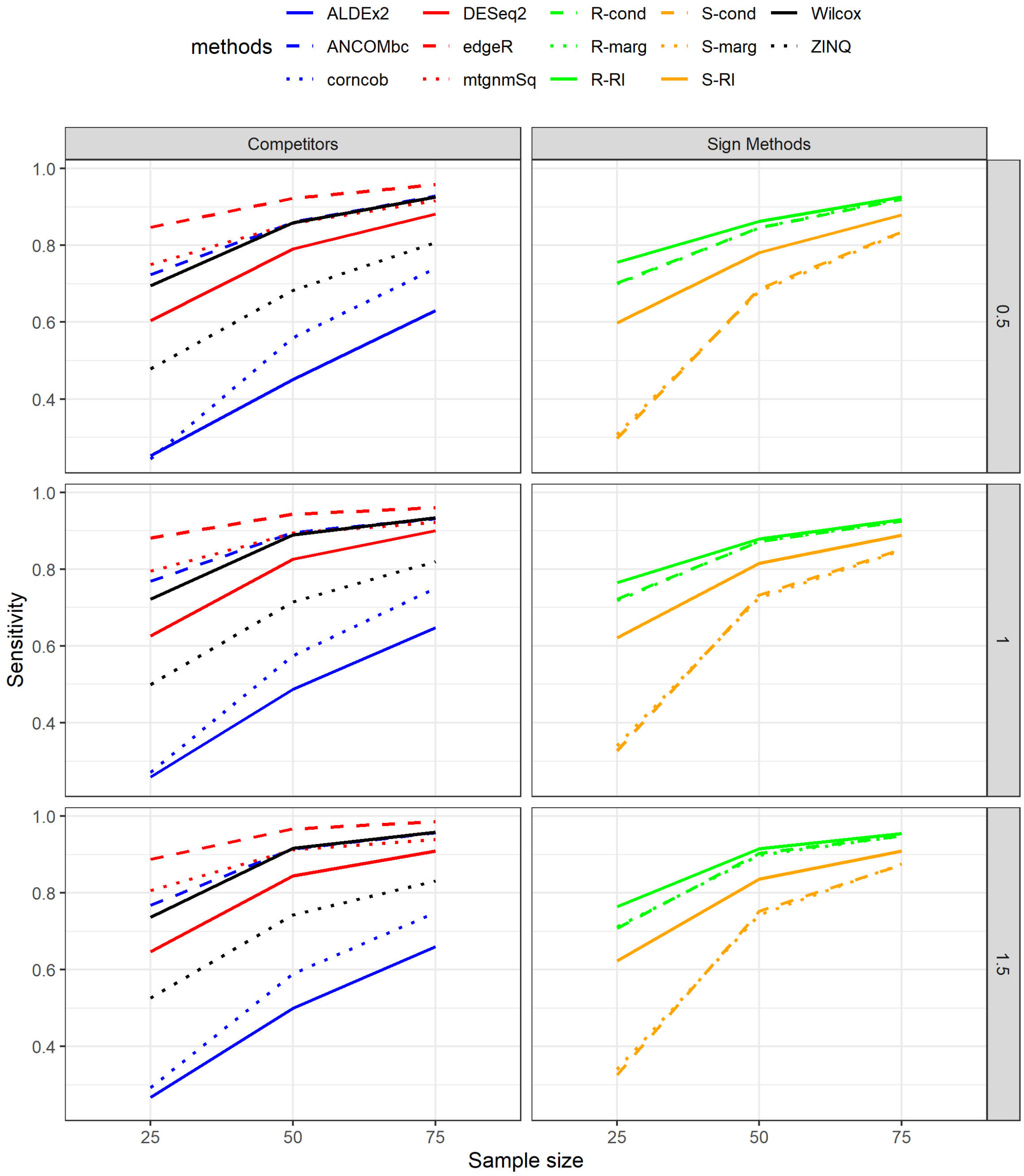

Figure 4: Empirical sensitivities for the SPSimSeq simulations for setting B (low sparsity) for the new methods (left) and the competitors (right) with increasing sample size (25,50,75) and increasing log-fold change (0.5,1,1.5) and 10% DA.

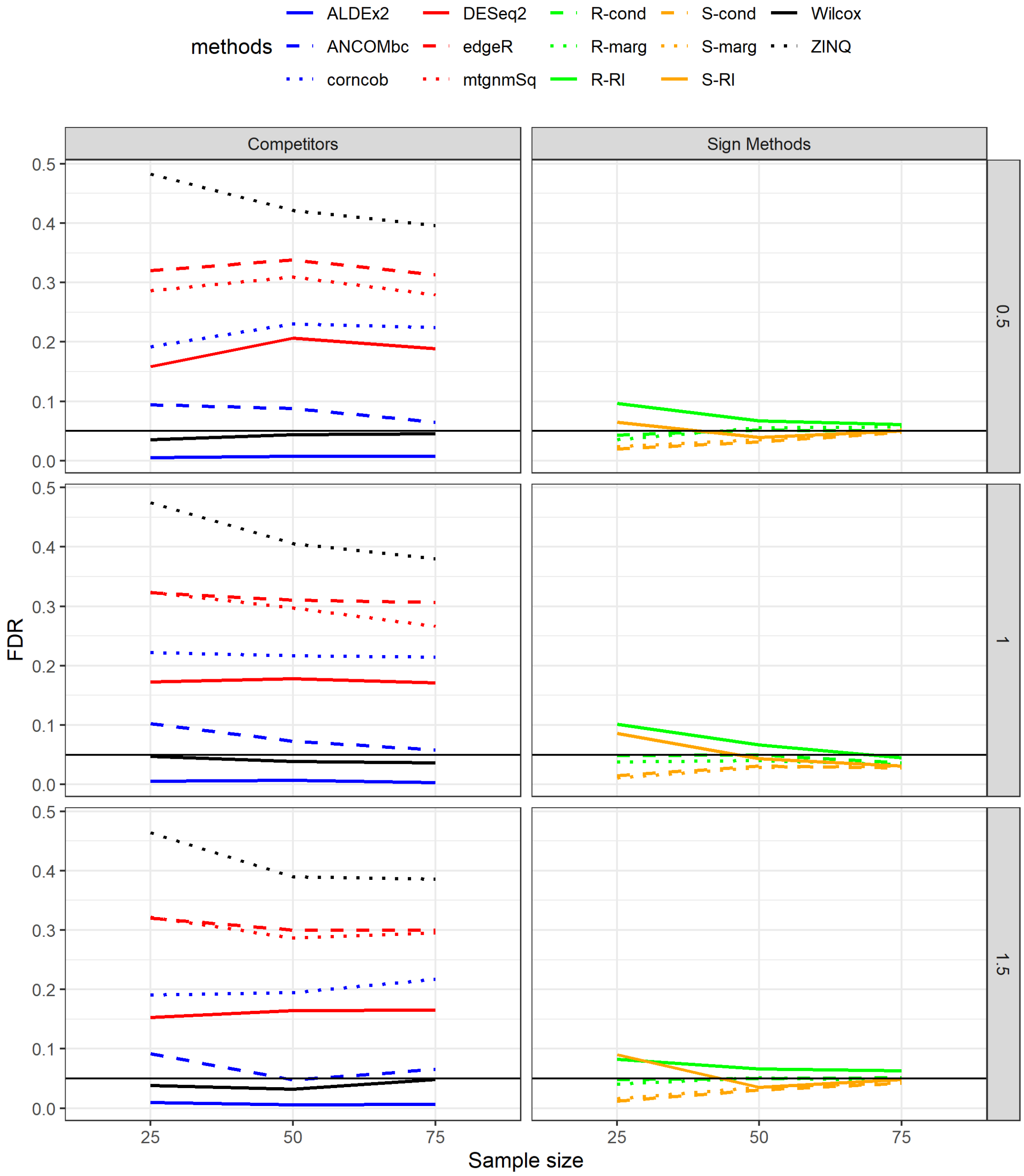

Figure 5: Empirical FDRs for the SPSimSeq simulations for setting B (low sparsity) for the new methods (left) and the competitors (right) with increasing sample size (25,50,75) and increasing log-fold change (0.5,1,1.5) and 10% DA. The nominal FDR was set on 0.05 (solid line).

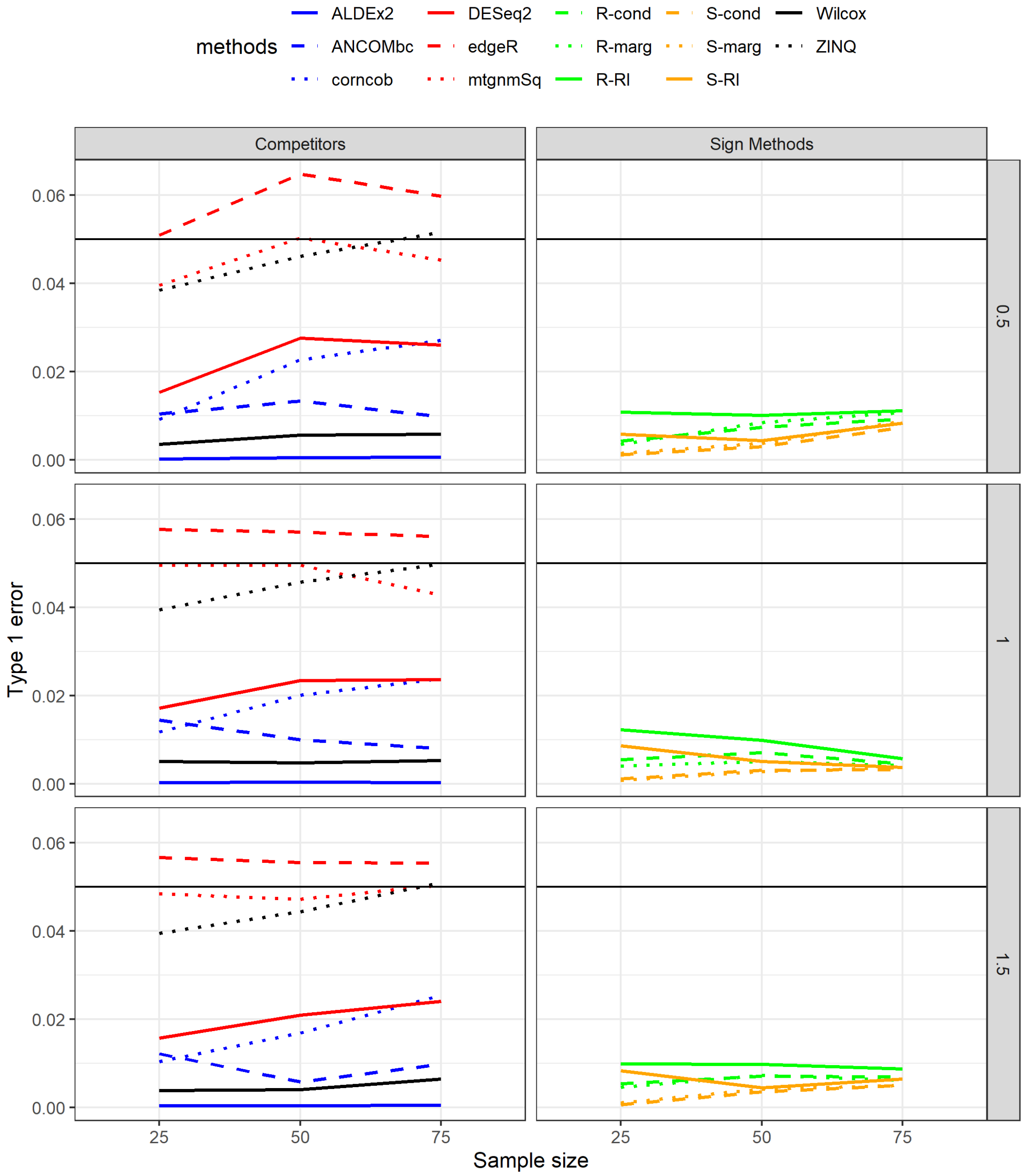

Figure 6: Empirical Type 1 error rate for the SPSimSeq simulations for setting B (low sparsity) for the new methods (right) and competitors (left) with increasing sample size (25,50,75), increasing log-fold change (0.5,1,1.5) and 10% DA.

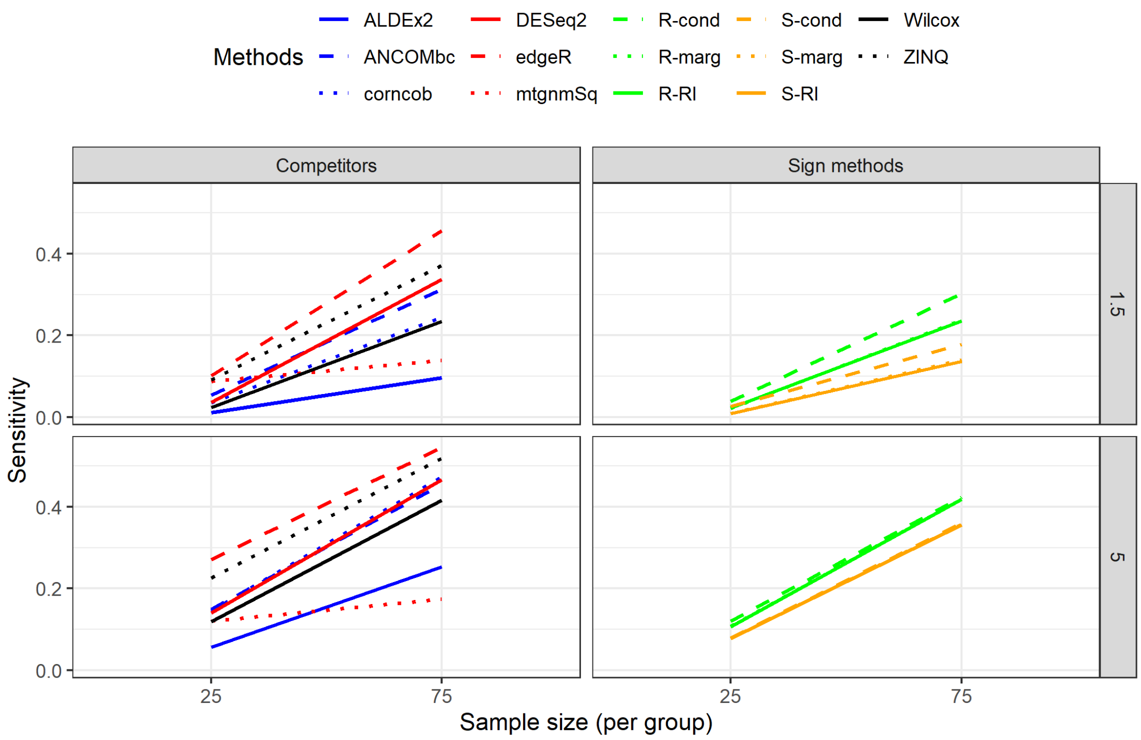

Figure 7: Empirical sensitivities for the NB simulations for setting A (high sparsity) for the new methods (right) and competitors (left) with increasing sample size (25,75), increasing fold change (1.5, 5)and 10% DA.

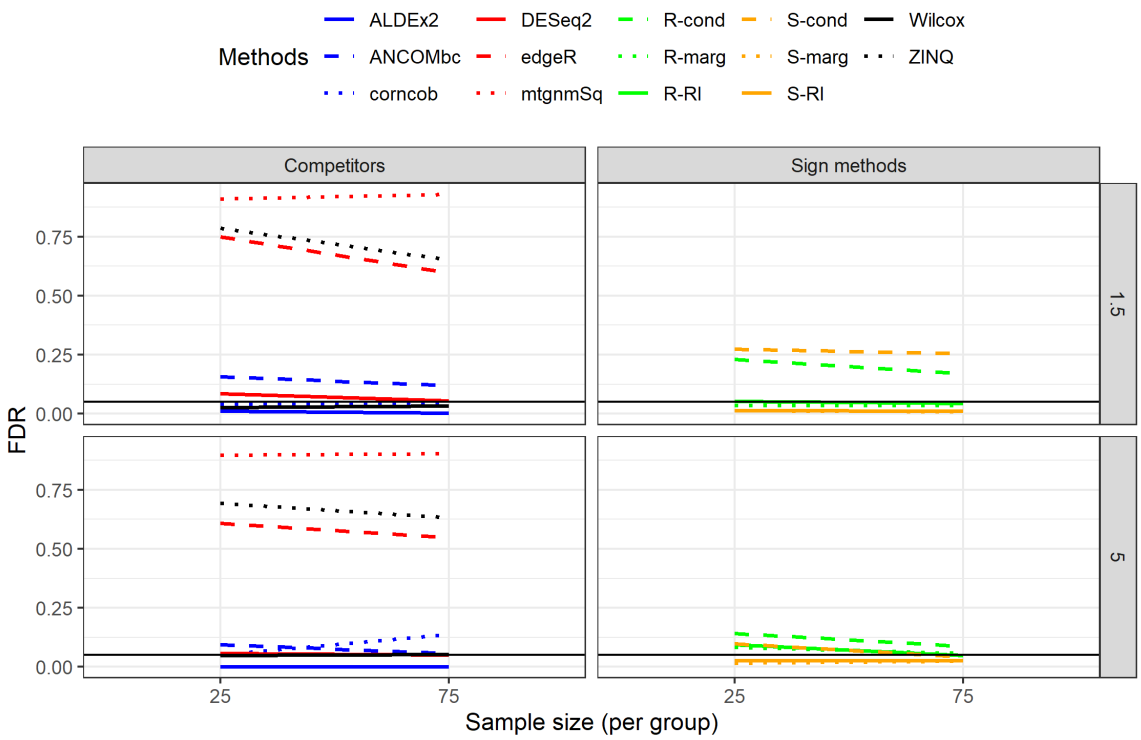

Figure 8: Empirical FDR for the NB simulations for setting A (high sparsity) for the new methods (right) and competitors (left) with increasing sample size (25,75), increasing fold change (1.5, 5) and 10% DA. The nominal FDR was set on 0.05 (solid line).

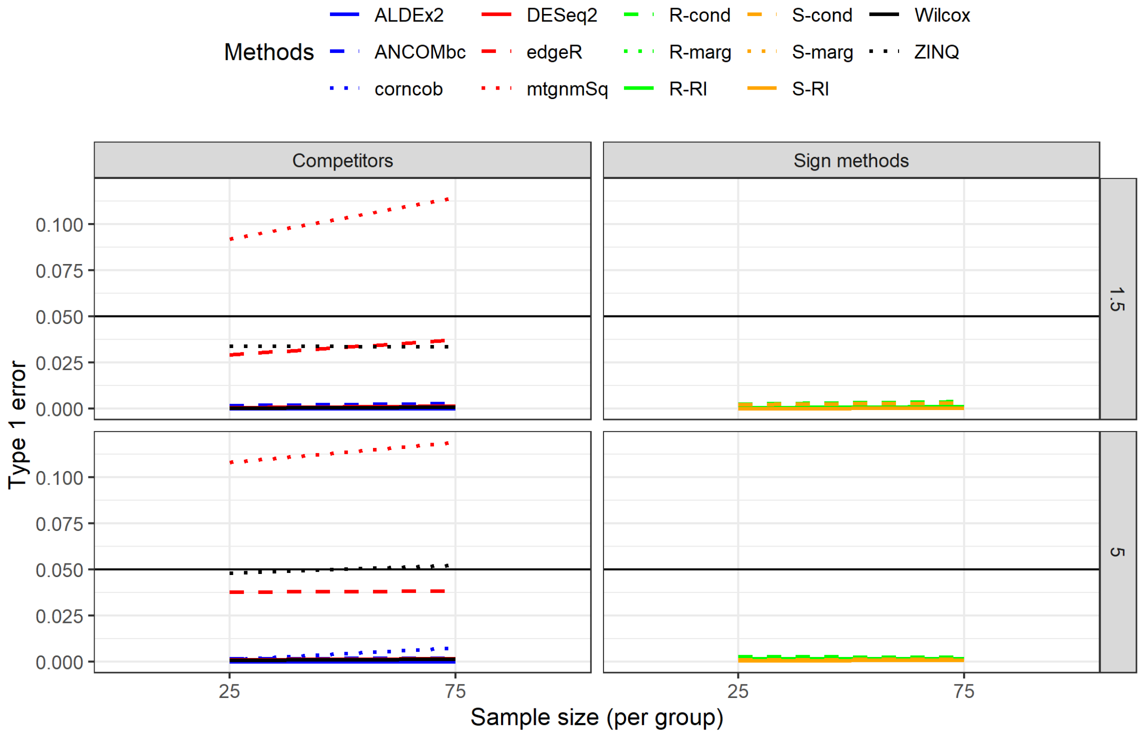

Figure 9: Empirical Typer I error rate for the NB simulations for setting A (high sparsity) for the new methods (right) and competitors (left) with increasing sample size (25,75), increasing fold change (1.5, 5) and 10% DA.

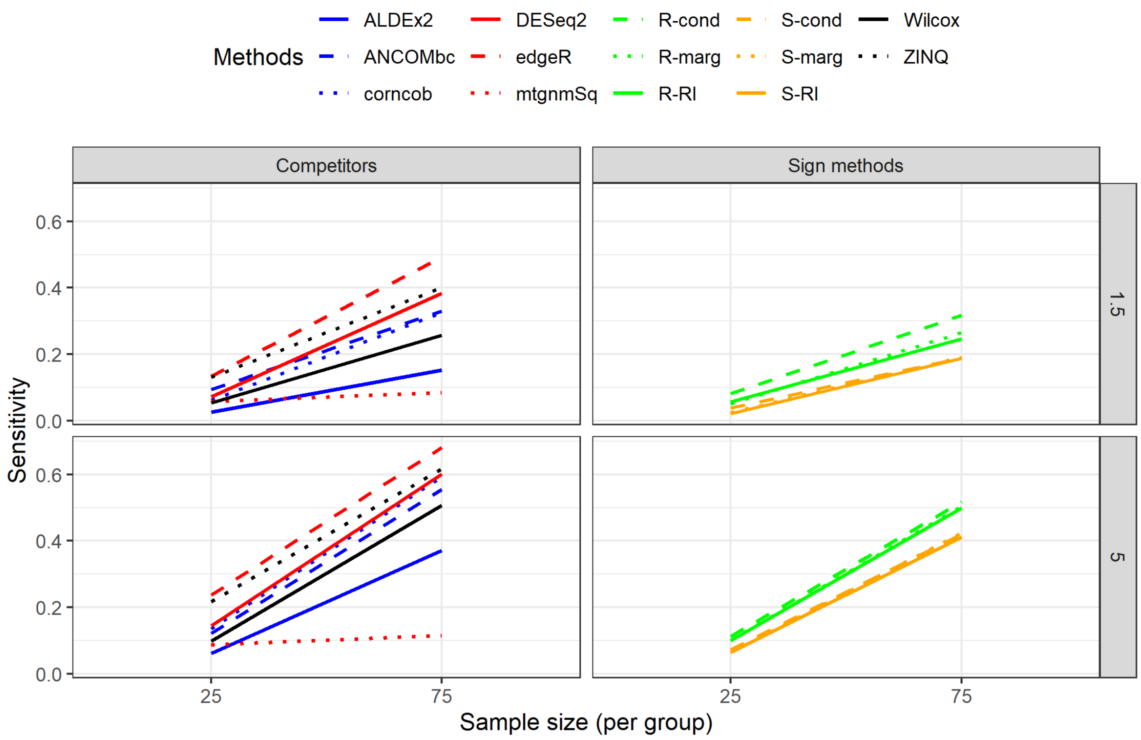

Figure 10: Empirical sensitivities for the NB simulations for setting B (low sparsity) for the new methods (right) and competitors (left) with increasing sample size (25,75), increasing fold change (1.5, 5) and 10% DA.

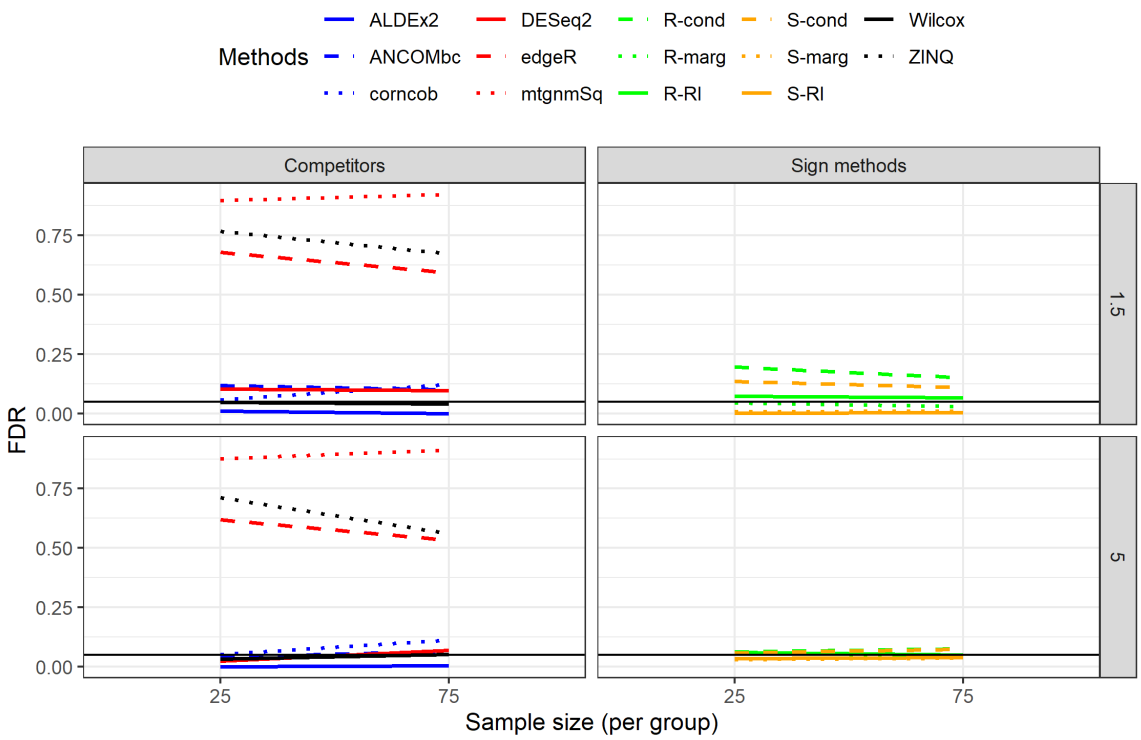

Figure 11: Empirical FDR for the NB simulations for setting B (low sparsity) for the new methods (right) and competitors (left) with increasing sample size (25,75), increasing fold change (1.5, 5) and 10% DA. The nominal FDR was set on 0.05 (solid line).

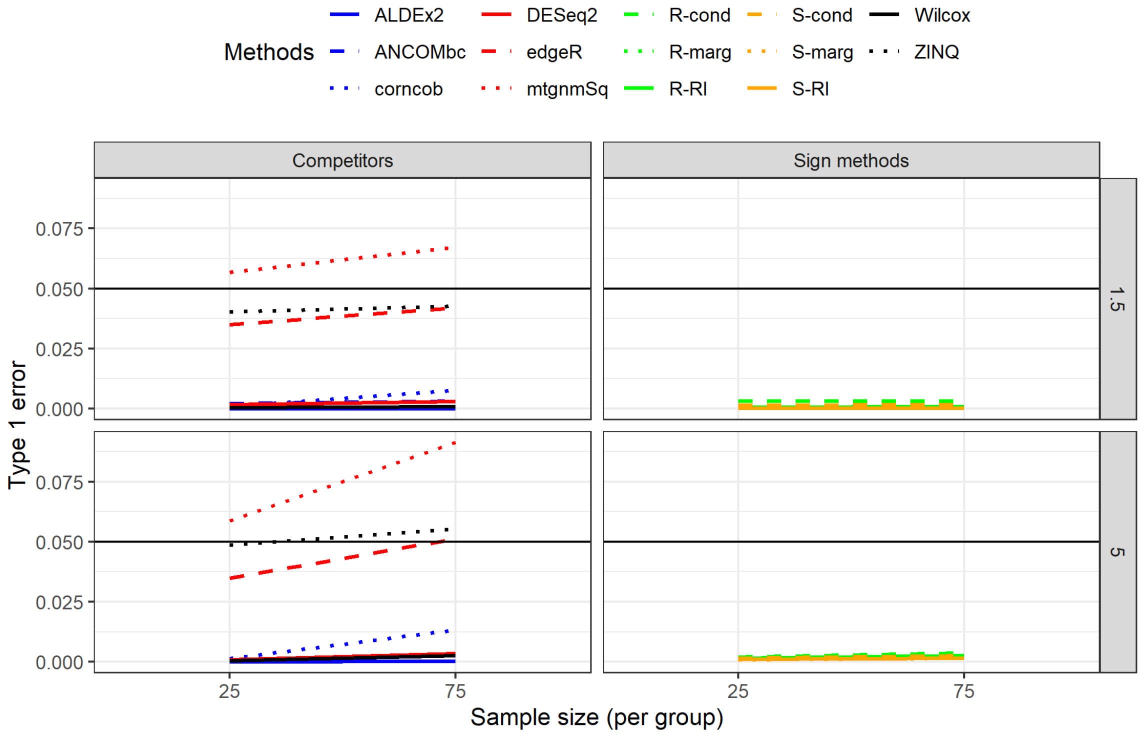

Figure 12: Empirical Type I error rate for the NB simulations for setting B (low sparsity) for the new methods (right) and competitors (left) with increasing sample size (25,75), increasing fold change (1.5, 5) and 10% DA.

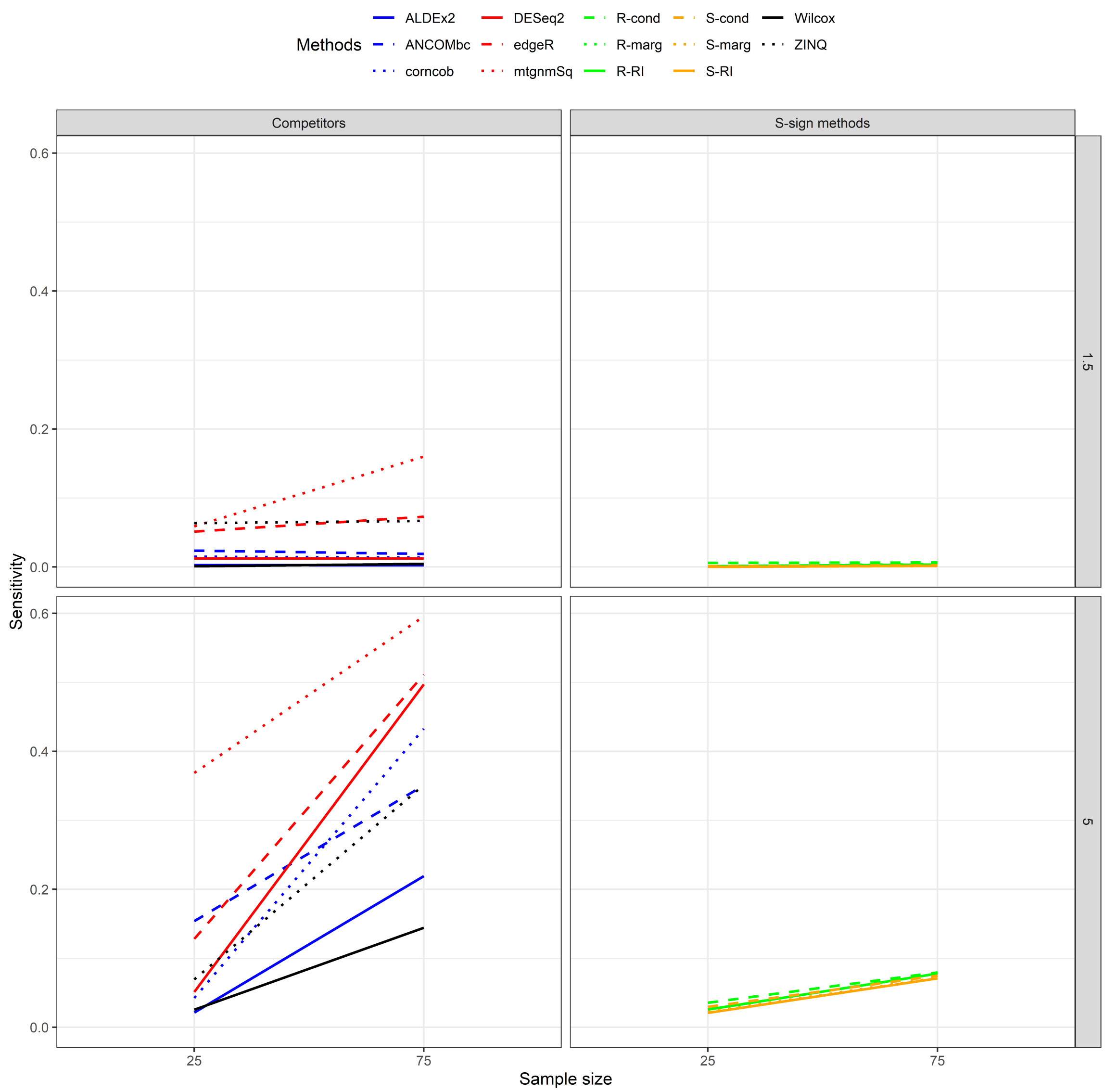

Figure 13: Empirical sensitivities for the NB simulations for setting A (high sparsity) for the new methods (right) and competitors (left) with increasing sample size (25,75), increasing fold change (1.5, 5) and 70% DA.

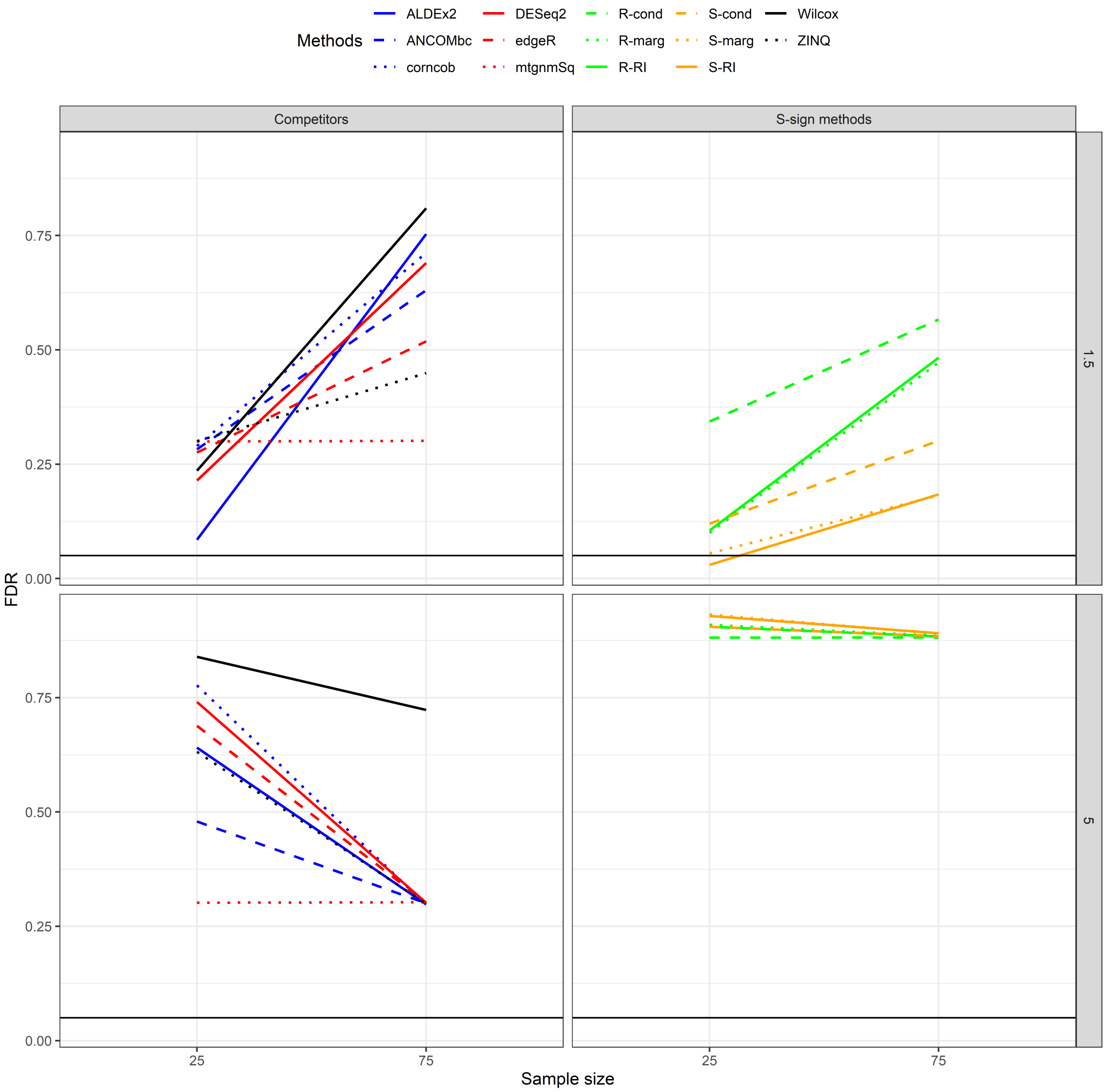

Figure 14: Empirical FDR for the NB simulations for setting A (high sparsity) for the new methods (right) and competitors (left) with increasing sample size (25,75), increasing fold change (1.5, 5) and 70% DA. The nominal FDR was set at 0.05 (solid line).

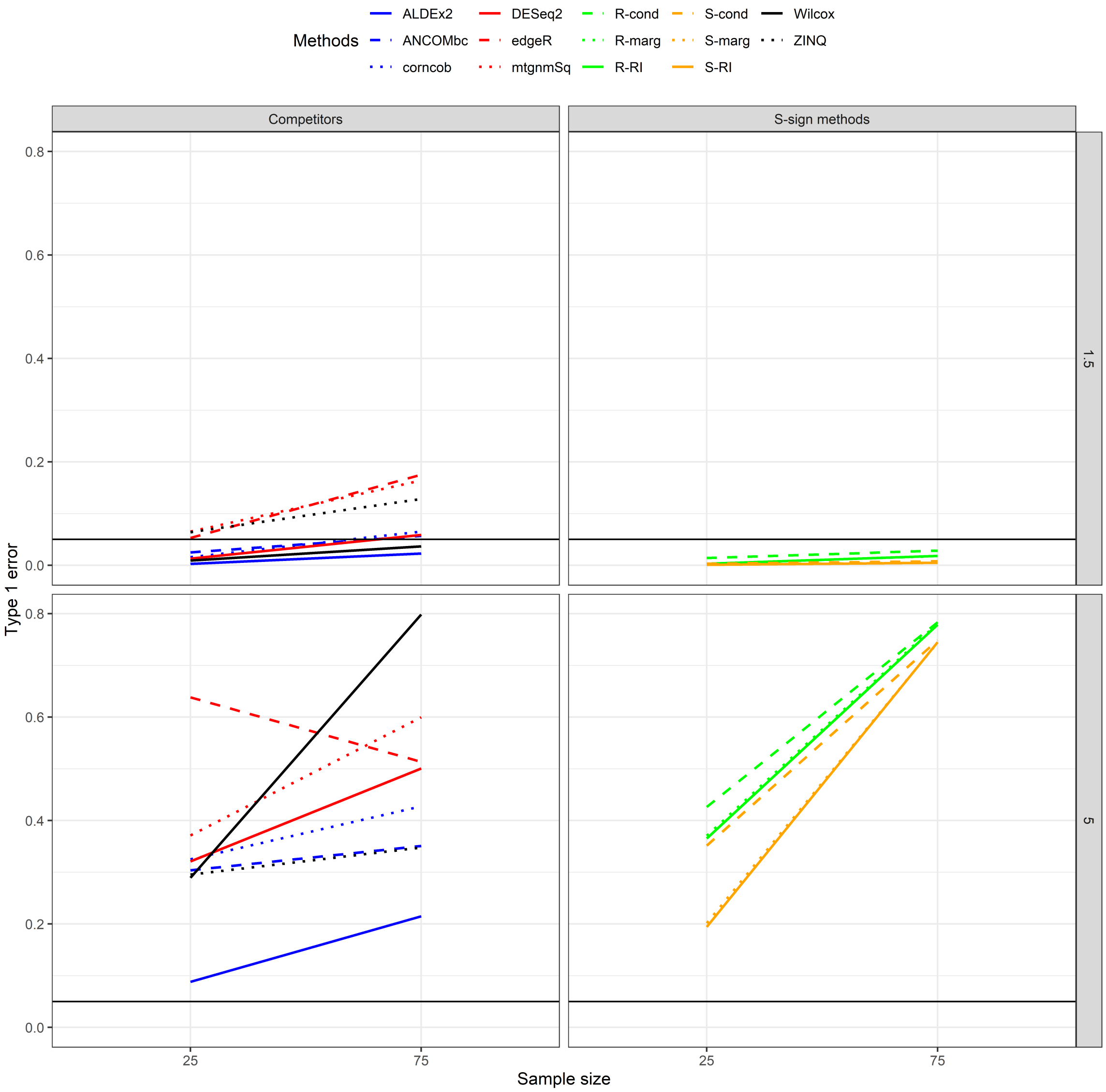

Figure 15: Empirical Type I error rate for the NB simulations for setting A (high sparsity) for the new methods (right) and competitors (left) with increasing sample size (25,75), increasing fold change (1.5, 5) and 70% DA.

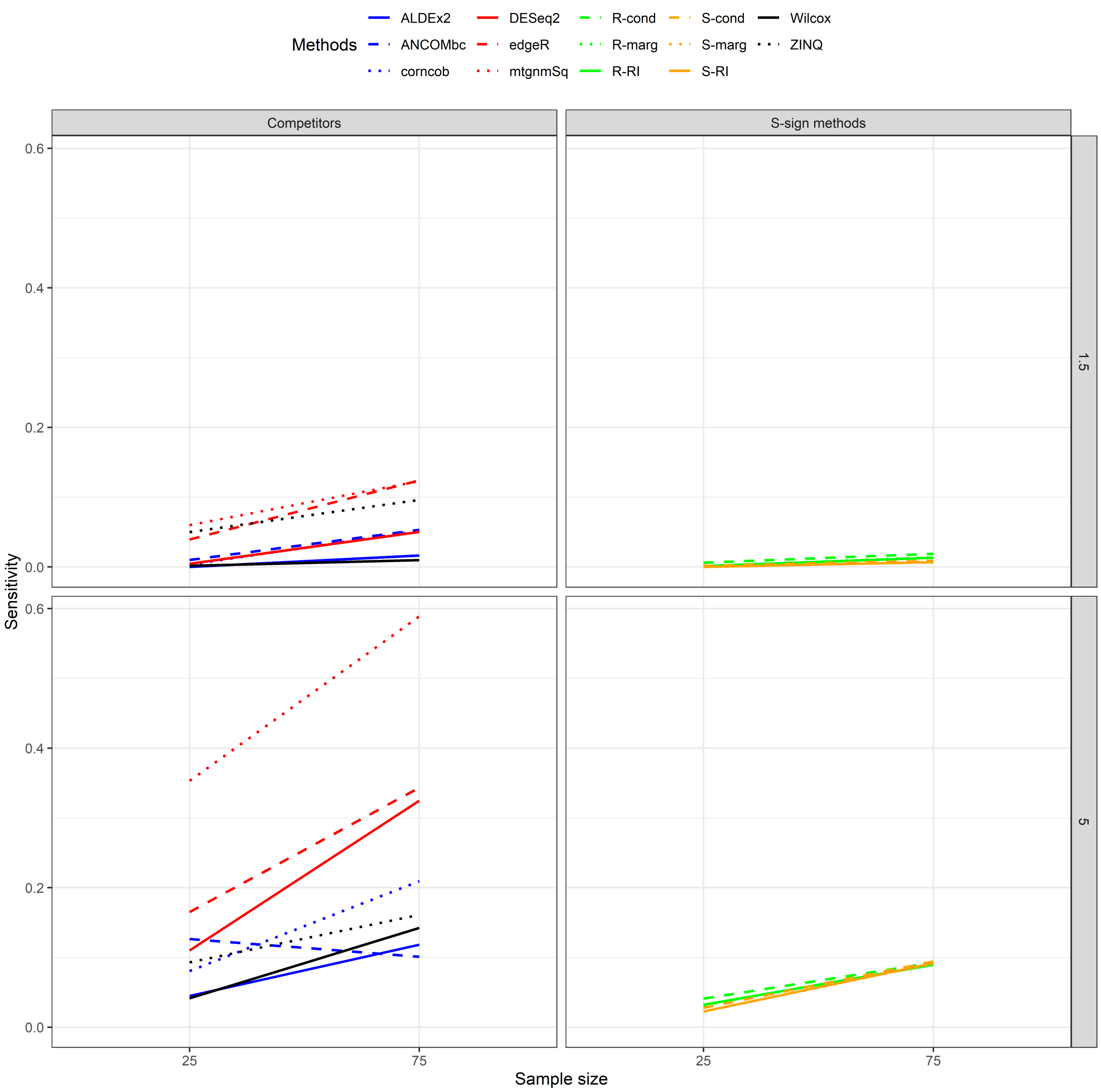

Figure 16: Empirical sensitivities for the NB simulations for setting B (low sparsity) for the new methods (right) and competitors (left) with increasing sample size (25,75), increasing fold change (1.5, 5) and 70% DA.

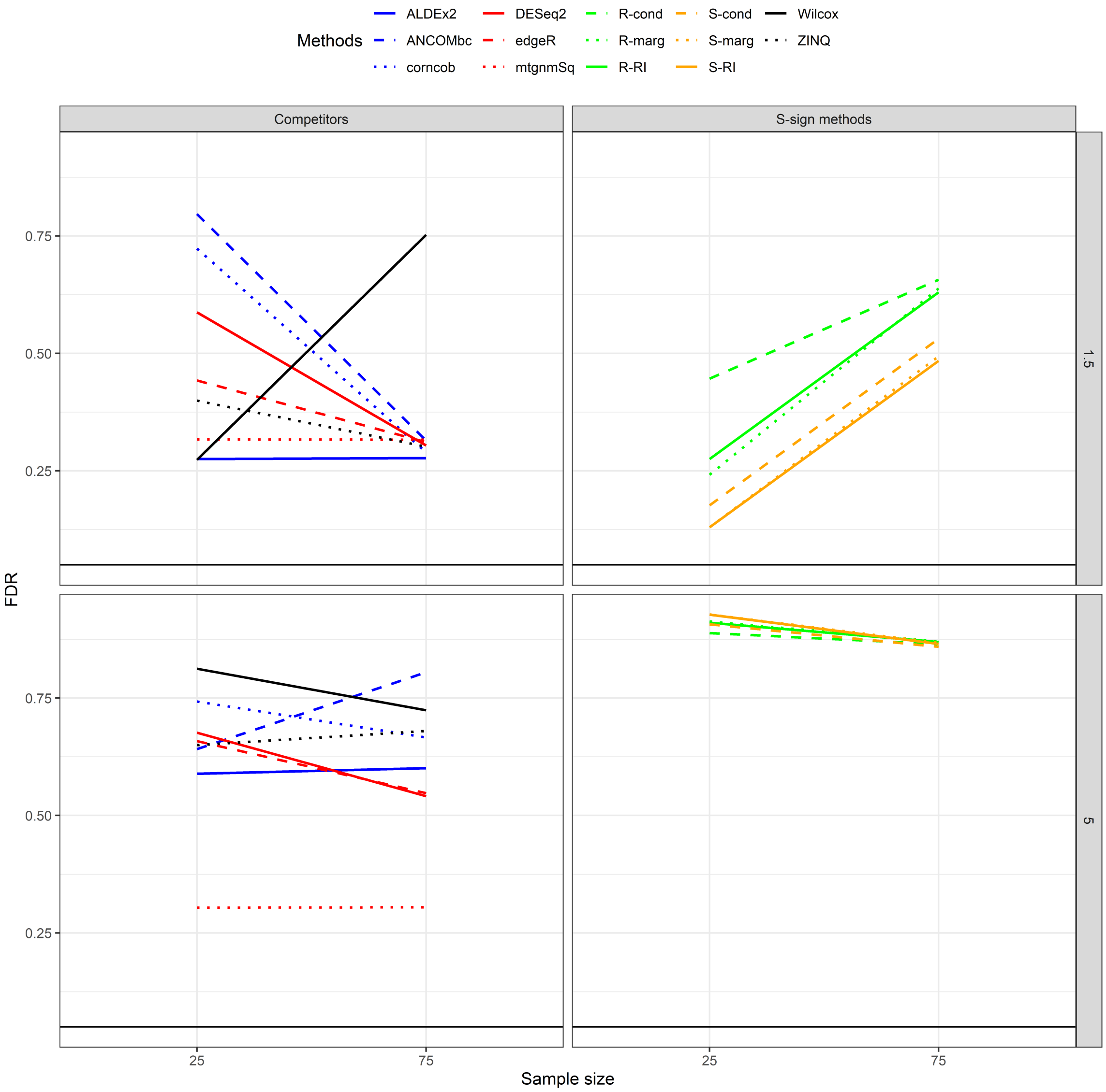

Figure 17: Empirical FDR for the NB simulations for setting B (low sparsity) for the new methods (right) and competitors (left) with increasing sample size (25,75), increasing fold change (1.5, 5) and 70% DA. The nominal FDR was set at 0.05 (solid line).

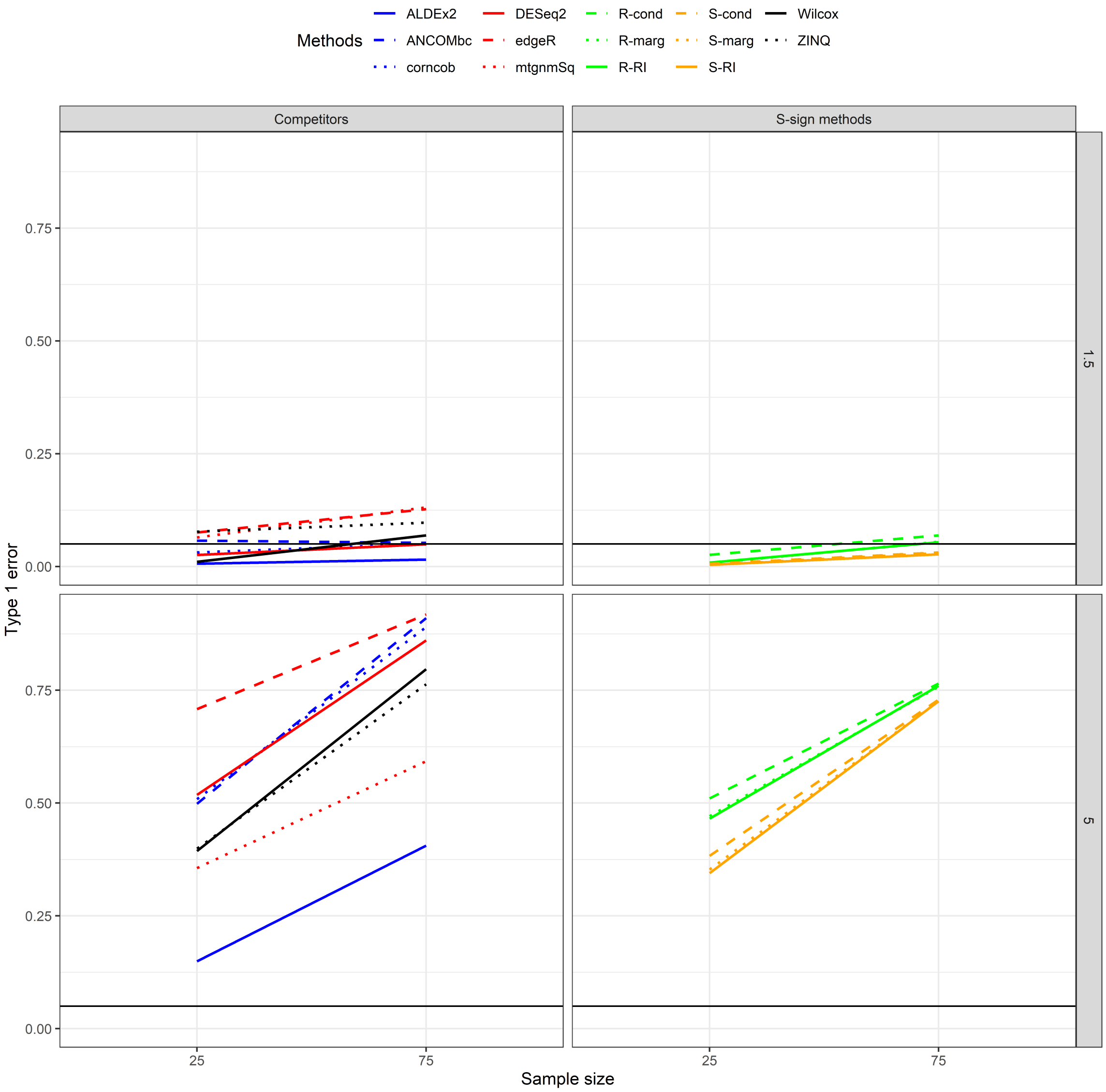

Figure 18: Empirical Type I error rate for the NB simulations for setting B (low sparsity) for the new methods (right) and competitors (left) with increasing sample size (25,75), increasing fold change (1.5, 5) and 70% DA.

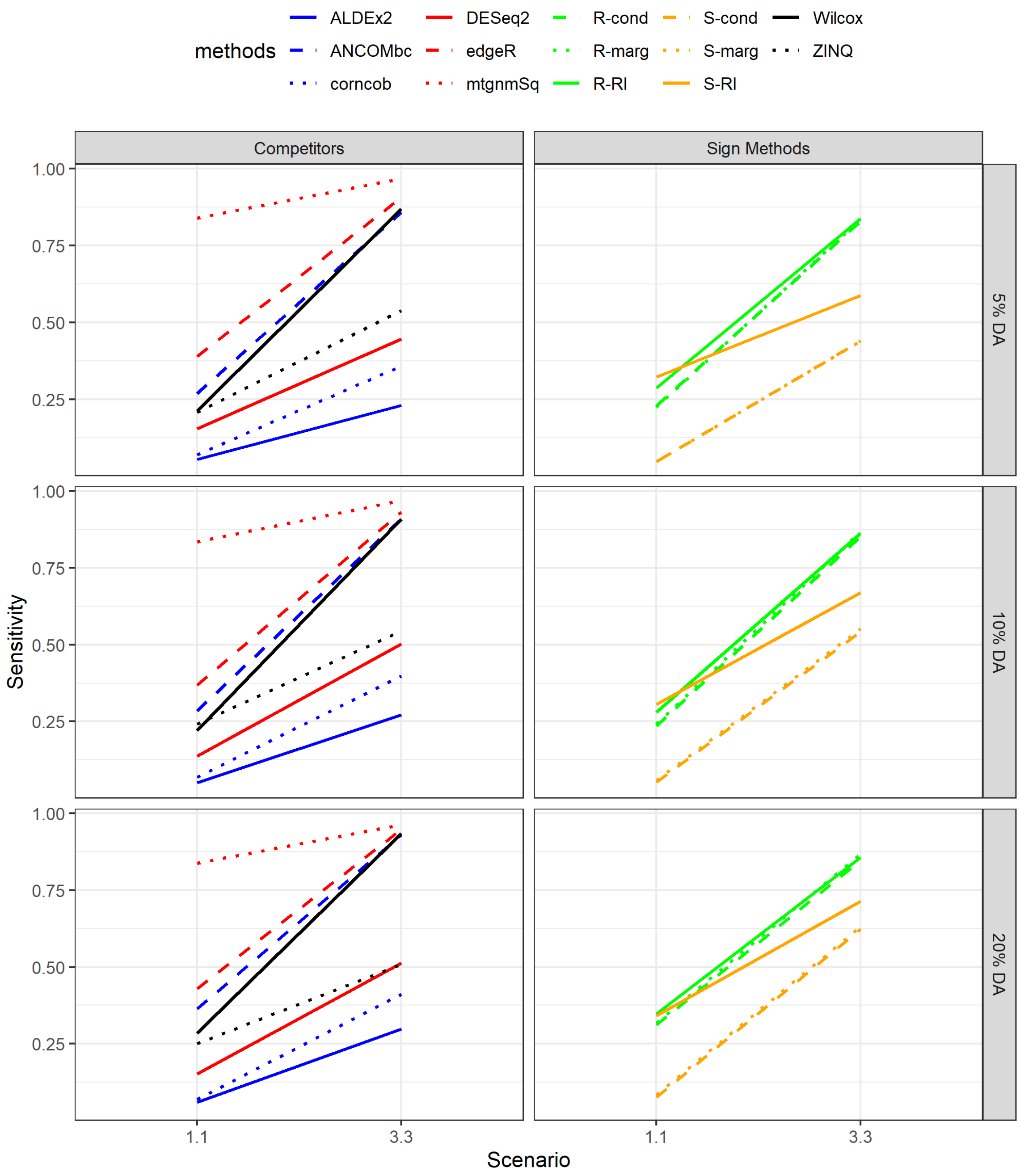

Figure 19: Empirical sensitivities for the SPSimSeq simulations for setting A (high sparsity) scenario 1.1 and 3.3 for the new methods (right) and competitors (left) with increasing differential abundance rate (5%, 10% and 20%).

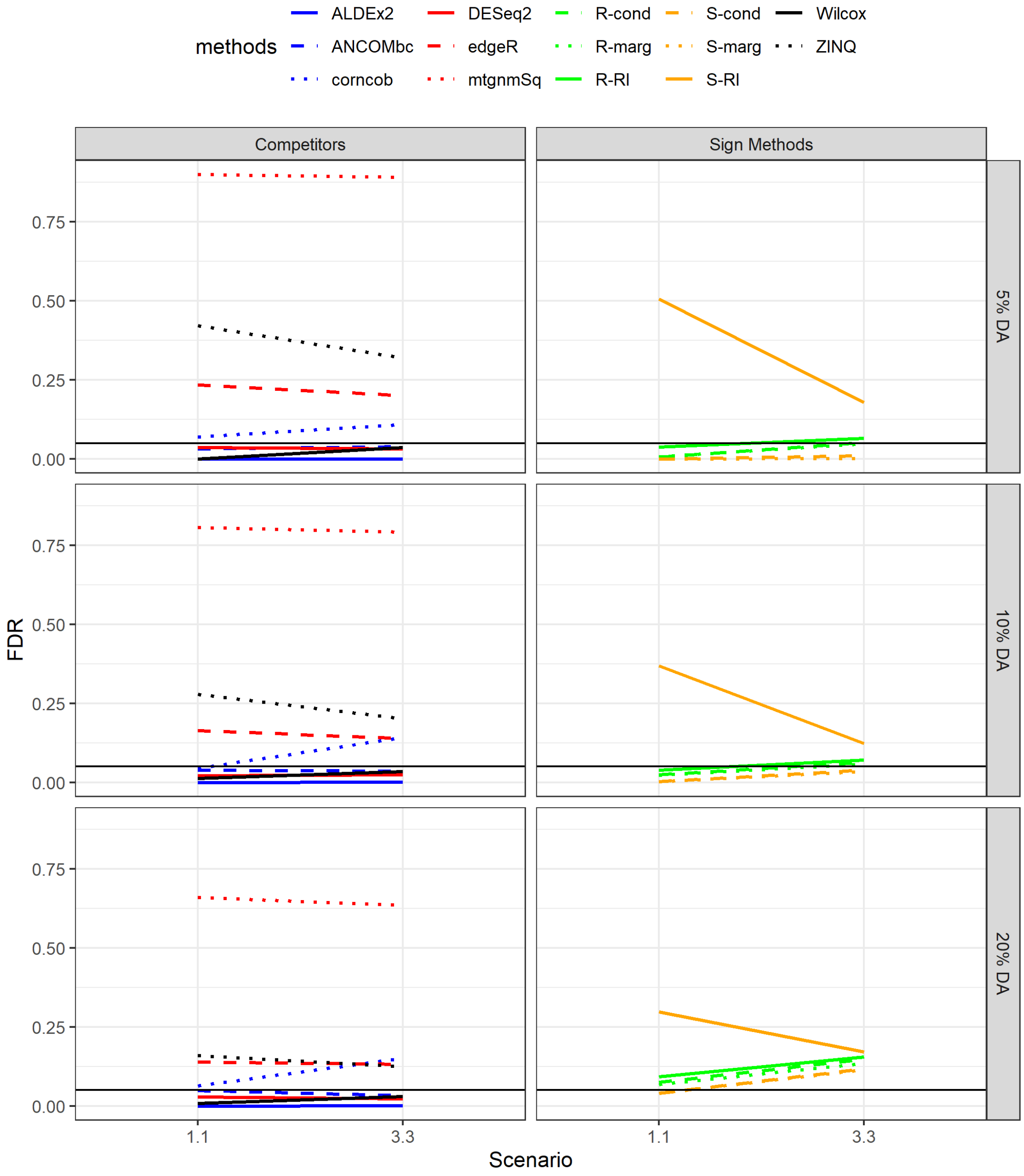

Figure 20: Empirical FDRs for the SPSimSeq simulations for setting A (high sparsity) scenario 1.1 and 3.3 for the new methods (right) and competitors (left) with increasing differential abundance rate (5%, 10% and 20%).

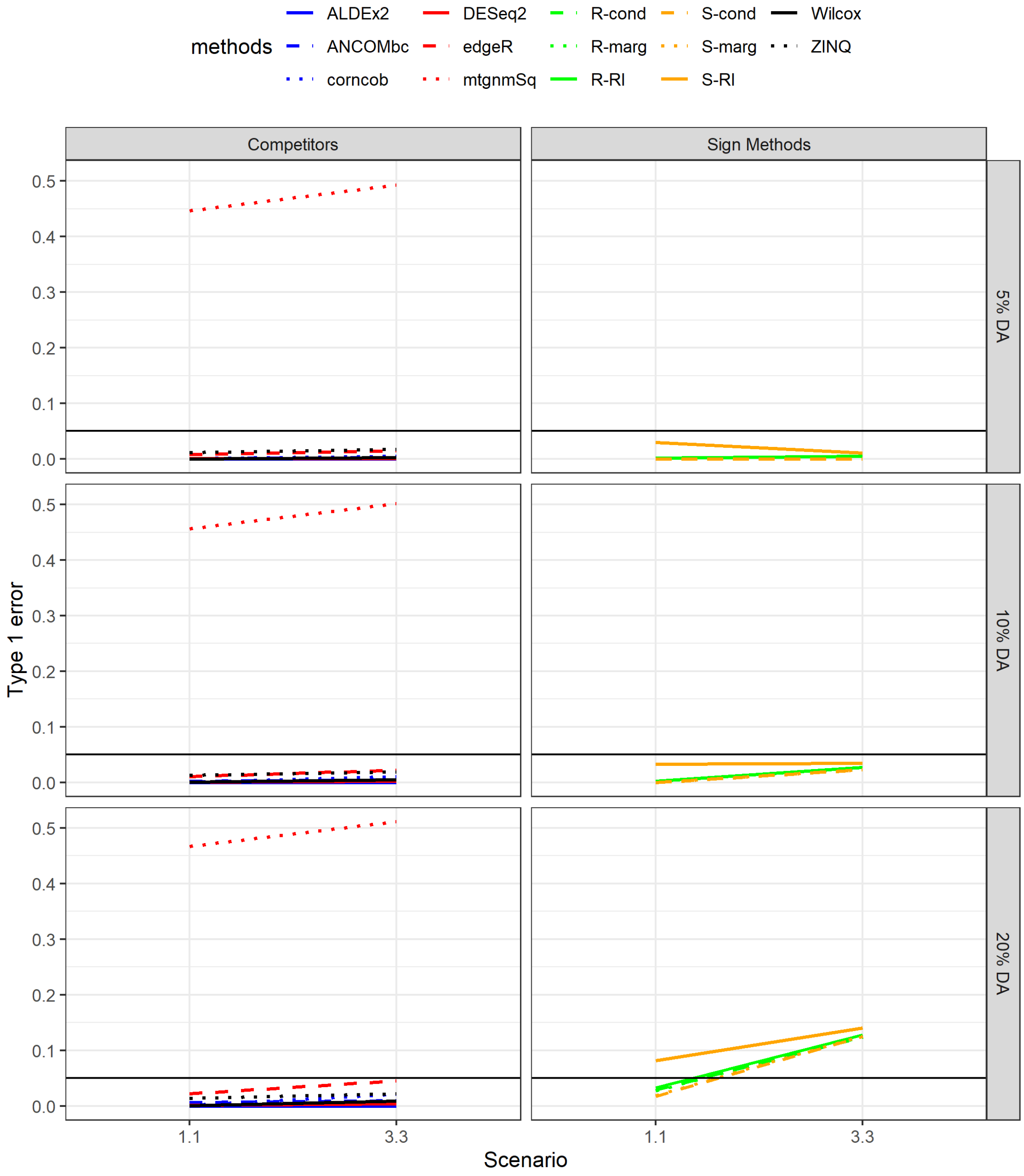

Figure 21: Empirical Type I error rate for the SPSimSeq simulations for setting A (high sparsity) scenario 1.1 and 3.3 for the new methods (right) and competitors (left) with increasing differential abundance rate (5%, 10% and 20%).

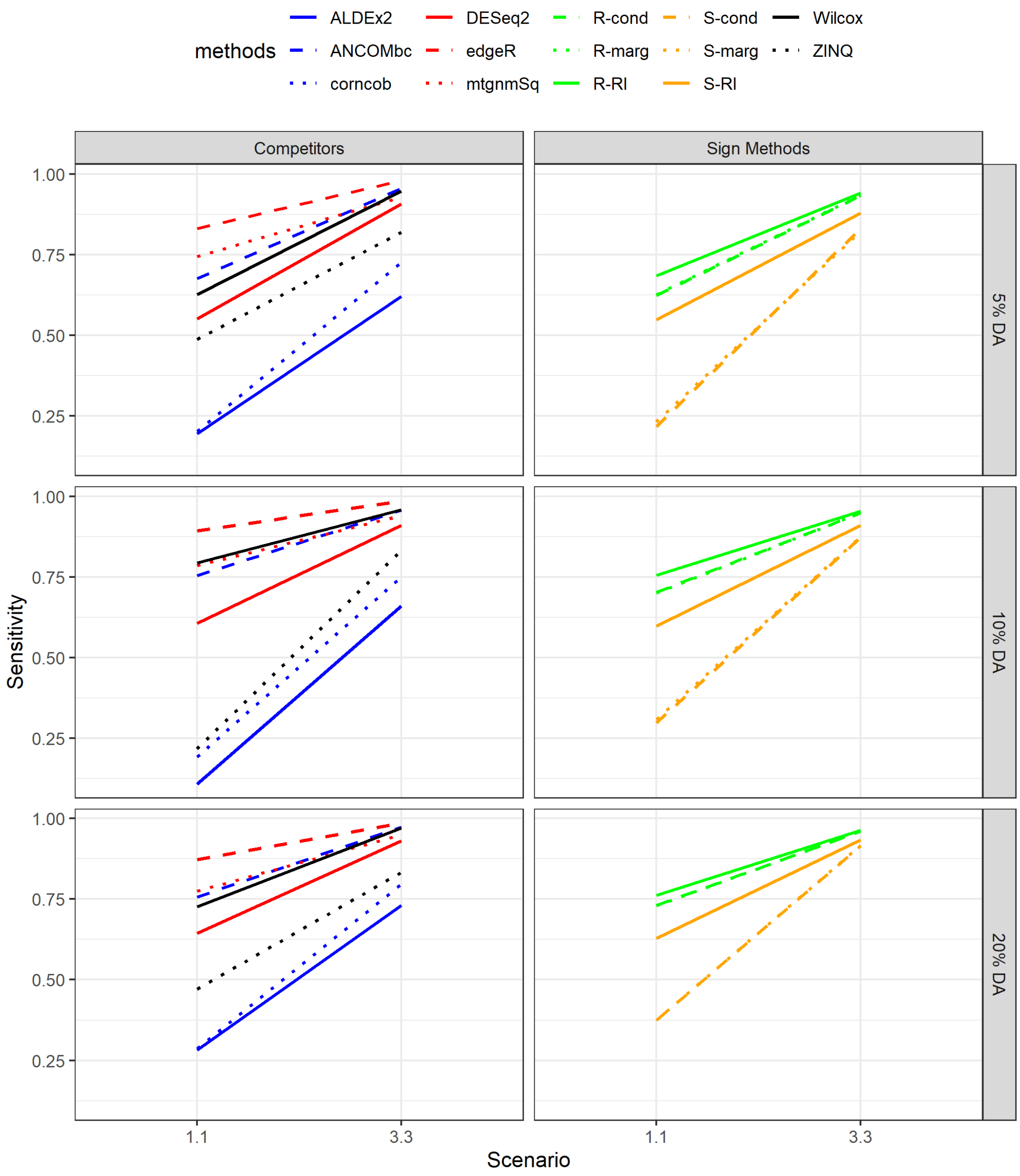

Figure 22: Empirical sensitivities for the SPSimSeq simulations for setting B (low sparsity) scenario 1.1 and 3.3 for the new methods (right) and competitors (left) with increasing differential abundance rate (5%, 10% and 20%).

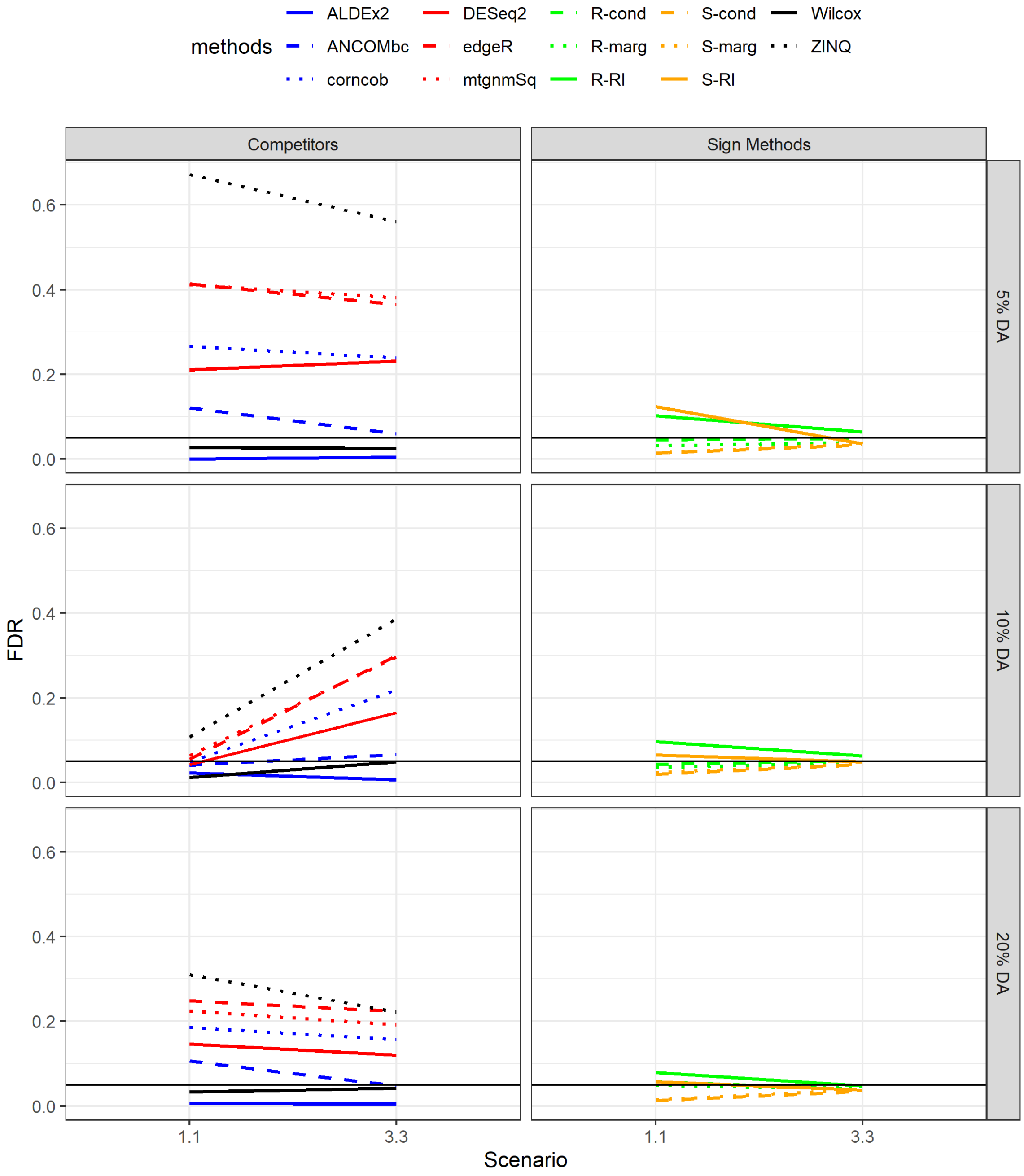

Figure 23: Empirical FDRs for the SPSimSeq simulations for setting B (low sparsity) scenario 1.1 and 3.3 for the new methods (right) and competitors (left) with increasing differential abundance rate (5%, 10% and 20%).

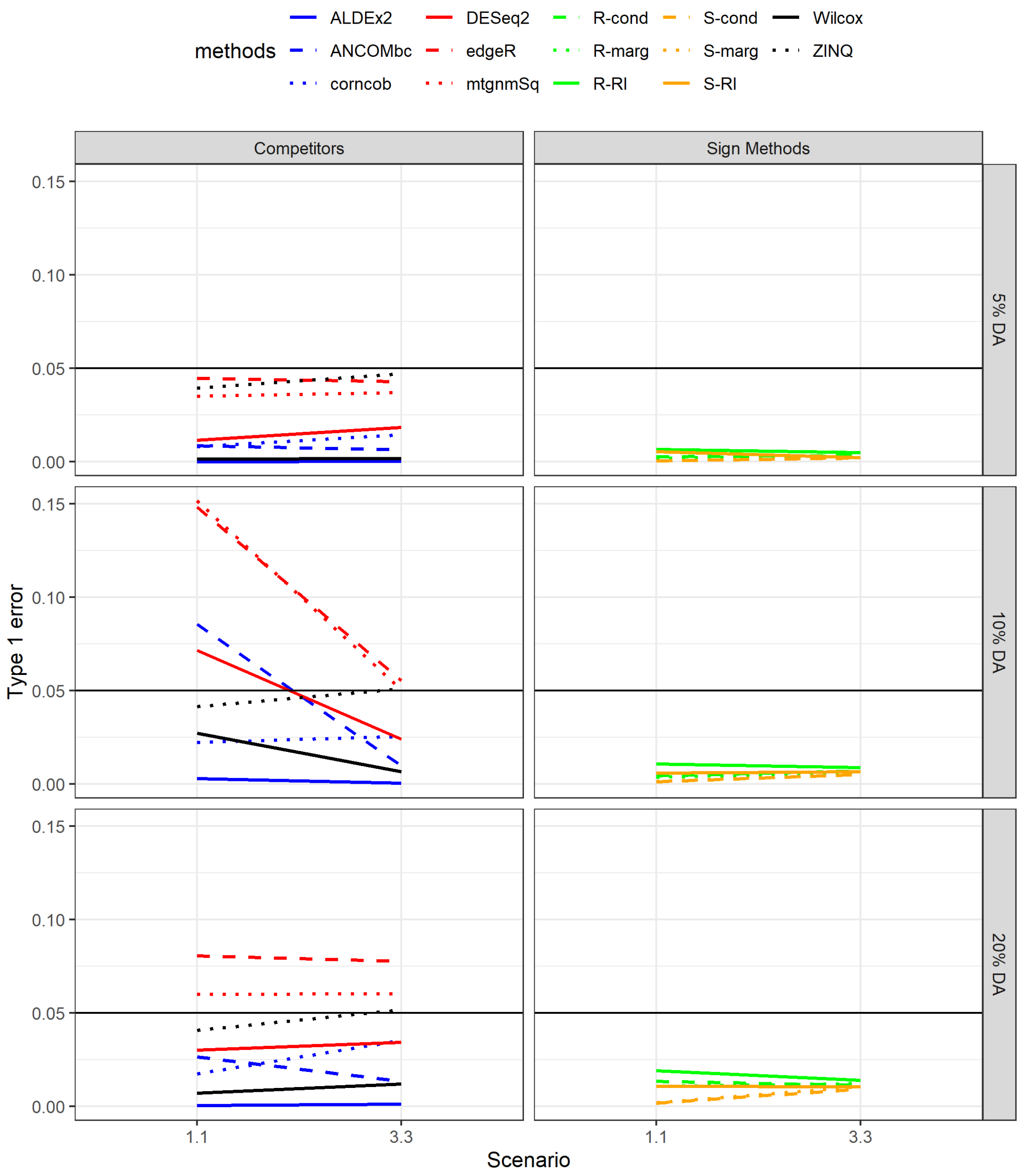

Figure 24: Empirical Type I error rate for the SPSimSeq simulations for setting B (low sparsity) scenario 1.1 and 3.3 for the new methods (right) and competitors (left) with increasing differential abundance rate (5%, 10% and 20%).

Table 1: Empirical FDR for the SPSimSeq simulations in setting A and B, for the three thresholds of the log-fold change (0.5, 1 and 1.5), with a sample size of 25 per group and 10% DA. The nominal FDR was set to 5%. Empirical FDRs larger than 10% are printed in bold face.

Method		Setting A			Setting B		
		0.5	1	1.5	0.5	1	1.5
S-Sign	marginal	0.003	0.009	0.014	0.024	0.011	0.016
	conditional	0.003	0.009	0.009	0.020	0.015	0.012
	RI	**0.370**	**0.360**	**0.344**	0.065	0.086	0.091
R-Sign	marginal	0.024	0.040	0.049	0.035	0.039	0.041
	conditional	0.025	0.037	0.061	0.043	0.049	0.049
	RI	0.040	0.064	0.080	0.097	**0.101**	0.082
Competitors	ALDEx2	0.000	0.000	0.000	0.001	0.001	0.010
	ANCOM-BC	0.039	0.040	0.030	0.094	**0.103**	0.092
	corncob	0.045	0.039	0.050	**0.192**	**0.223**	**0.191**
	DESeq2	0.021	0.029	0.031	**0.159**	**0.173**	**0.153**
	edgeR	**0.164**	**0.158**	**0.155**	**0.320**	**0.323**	**0.320**
	metagenomeSeq	**0.807**	**0.791**	**0.783**	**0.286**	**0.324**	**0.322**
	Wilcoxon	0.013	0.020	0.006	0.036	0.047	0.038
	ZINQ	**0.241**	**0.206**	**0.224**	**0.482**	**0.474**	**0.464**

Table 2: Empirical FDR for the SPSimSeq simulations in setting A and B, for the three thresholds of the log-fold change (0.5, 1 and 1.5), with a sample size of 50 per group and 10% DA. The nominal FDR was set to 5%. Empirical FDRs larger than 10% are printed in bold face.

Method		Setting A			Setting B		
		0.5	1	1.5	0.5	1	1.5
S-Sign	marginal	0.037	0.003	0.044	0.036	0.029	0.036
	conditional	0.033	0.005	0.042	0.031	0.031	0.031
	RI	**0.178**	**0.220**	**0.277**	0.040	0.044	0.036
R-Sign	marginal	0.075	0.038	0.094	0.055	0.041	0.051
	conditional	0.0756	0.045	**0.102**	0.052	0.050	0.050
	RI	0.064	0.055	**0.117**	0.067	0.067	0.067
Competitors	ALDEx2	0.002	0.000	0.000	0.008	0.008	0.006
	ANCOM-BC	0.038	0.034	0.037	0.088	0.072	0.047
	corncob	**0.109**	**0.103**	**0.105**	**0.230**	**0.217**	**0.194**
	DESeq2	0.036	0.040	0.027	**0.207**	**0.178**	**0.165**
	edgeR	**0.210**	**0.142**	**0.148**	**0.338**	**0.311**	**0.300**
	metagenomeSeq	**0.817**	**0.811**	**0.806**	**0.310**	**0.297**	**0.287**
	Wilcoxon	0.027	0.020	0.025	0.044	0.039	0.033
	ZINQ	**0.309**	**0.227**	**0.196**	**0.422**	**0.406**	**0.390**

Table 3: Empirical FDR for the SPSimSeq simulations in setting A and B, for the three thresholds of the log-fold change (0.5, 1 and 1.5), with a sample size of 75 per group and 10% DA. The nominal FDR was set to 5%. Empirical FDRs larger than 10% are printed in bold face.

Method		Setting A			Setting B		
		0.5	1	1.5	0.5	1	1.5
S-Sign	Marginal	0.029	0.025	0.034	0.053	0.031	0.043
	Conditional	0.025	0.025	0.039	0.048	0.028	0.042
	RI	**0.278**	**0.132**	**0.123**	0.049	0.031	0.049
R-Sign	Marginal	0.072	0.063	0.057	0.057	0.034	0.047
	Conditional	0.067	0.072	0.064	0.050	0.037	0.052
	RI	0.081	0.074	0.071	0.061	0.045	0.063
Competitors	ALDEx2	0.003	0.000	0.001	0.008	0.003	0.007
	ANCOM-BC	0.044	0.037	0.037	0.065	0.058	0.066
	corncob	**0.126**	0.098	**0.142**	**0.224**	**0.215**	**0.218**
	DESeq2	0.043	0.022	0.025	**0.189**	**0.171**	**0.165**
	edgeR	**0.201**	**0.180**	**0.140**	**0.313**	**0.307**	**0.300**
	metagenomeSeq	**0.807**	**0.800**	**0.793**	**0.279**	**0.266**	**0.296**
	Wilcoxon	0.042	0.040	0.035	0.045	0.037	0.049
	ZINQ	**0.313**	**0.245**	**0.203**	**0.396**	**0.380**	**0.386**
