# Supplementary material for: Simple and flexible sign and rank-based methods for testing for differential abundance in microbiome studies
Source: PLoS One. 2023 Sep 26;18(9):e0292055. doi: 10.1371/journal.pone.0292055 (PMC10522045; doi:10.1371/journal.pone.0292055)
Supplement: S3 Appendix — (PDF) [file pone.0292055.s009.pdf]

S3 Appendix - Extra simulation results

In these additional simulations, we used 16S microbiome data from a study on the coinfection of Plasmodium vivax and Soil-Transmitted Heminths[1] as source data. Children with a coinfection were compared to children with no infection. The simulation framework of SPsimSeq was used; see details on the simulation framework in the main manuscript and S1 Appendix (Fig. 1). The resulting source datasets have a sparsity of 70% for setting A and 52% for Setting B.

List of Figures

1 Empirical sensitivities for the SPSimSeq simulations for setting A (high sparsity) for the new methods (left) and the competitors (right) with increasing sample size (25,50,75) and increasing log-fold change (0.5,1,1.5) and 10% DA. 4

2 Empirical FDRs for the SPSimSeq simulations for setting A (high sparsity) for the new methods (left) and the competitors (right) with increasing sample size (25,50,75) and increasing log-fold change (0.5,1,1.5) and 10% DA. The nominal FDR was set on 0.05 (solid line). 5

3 Empirical Type 1 error rate for the SPSimSeq simulations for setting A (high sparsity) for the new methods (right) and competitors (left) with increasing sample size (25,50,75),increasing log-fold change (0.5,1,1.5) and 10% DA. 6

4 Empirical sensitivities for the SPSimSeq simulations for setting B (low sparsity) for the new methods (left) and the competitors (right) with increasing sample size (25,50,75) and increasing log-fold change (0.5,1,1.5) and 10% DA. 7

5 Empirical FDRs for the SPSimSeq simulations for setting B (low sparsity) for the new methods (left) and the competitors (right) with increasing sample size (25,50,75) and increasing log-fold change (0.5,1,1.5) and 10% DA. The nominal FDR was set on 0.05 (solid line). 8

6 Empirical Type 1 error rate for the SPSimSeq simulations for setting B (low sparsity) for the new methods (right) and competitors (left) with increasing sample size (25,50,75),increasing log-fold change (0.5,1,1.5) and 10% DA. 9

7 The distributions of the library size, mean and variance of taxa abundance from the pre-processed data (Source_A) and for simulated data of SPsimSeq (scenario 2.2). 12

8 The relationship between the mean and coefficient of variation (CV) of taxa abundance levels (log-CPM) from the pre-processed data (Source_A) and for simulated data of SPsimSeq (scenario 2.2). 13

9 The fraction of zero counts per gene as a function of the mean taxa abundance levels (log-CPM) from the pre-processed data (Source_A) and for simulated data of SPsimSeq (scenario 2.2). 14

10 The distributions of the pairwise Pearson correlation-coefficients between taxa from the pre-processed data (Source_A) and for simulated data of SPsimSeq (scenario 2.2). 15

11 The distributions of the library size, mean and variance of taxa abundance from the pre-processed data (Source_B) and for simulated data of SPsimSeq (scenario 2.2). 16

12 The relationship between the mean and coefficient of variation (CV) of taxa abundance levels (log-CPM) from the pre-processed data (Source_B) and for simulated data of SPsimSeq (scenario 2.2). 17

13 The fraction of zero counts per gene as a function of the mean taxa abundance levels (log-CPM) from the pre-processed data (Source_B) and for simulated data of SPsimSeq (scenario 2.2). 18

14 The distributions of the pairwise Pearson correlation-coefficients between taxa from the pre-processed data (Source_B) and for simulated data of SPsimSeq (scenario 2.2). 19

Results

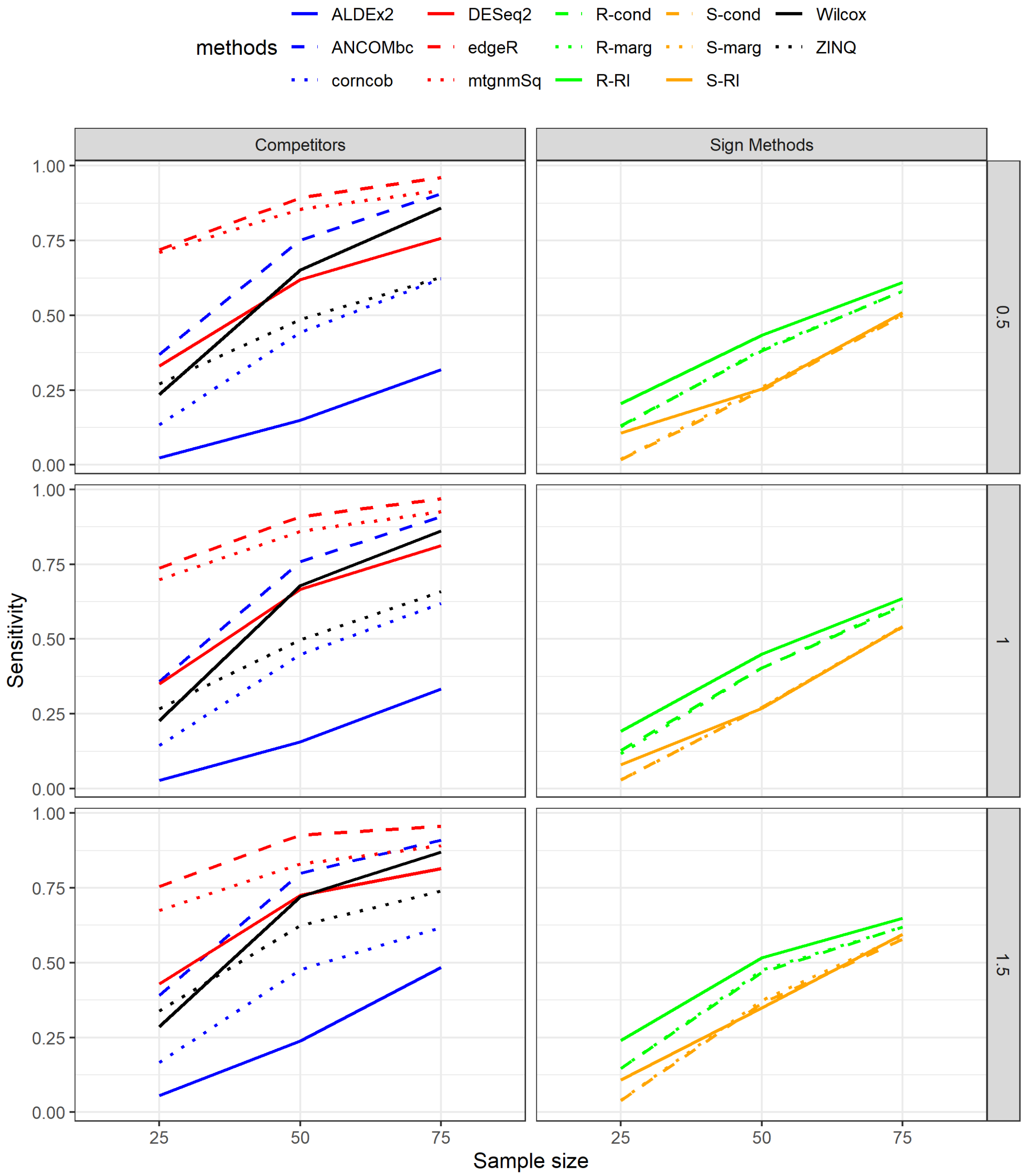

Figure 1: Empirical sensitivities for the SPSimSeq simulations for setting A (high sparsity) for the new methods (left) and the competitors (right) with increasing sample size (25,50,75) and increasing log-fold change (0.5,1,1.5) and 10% DA.

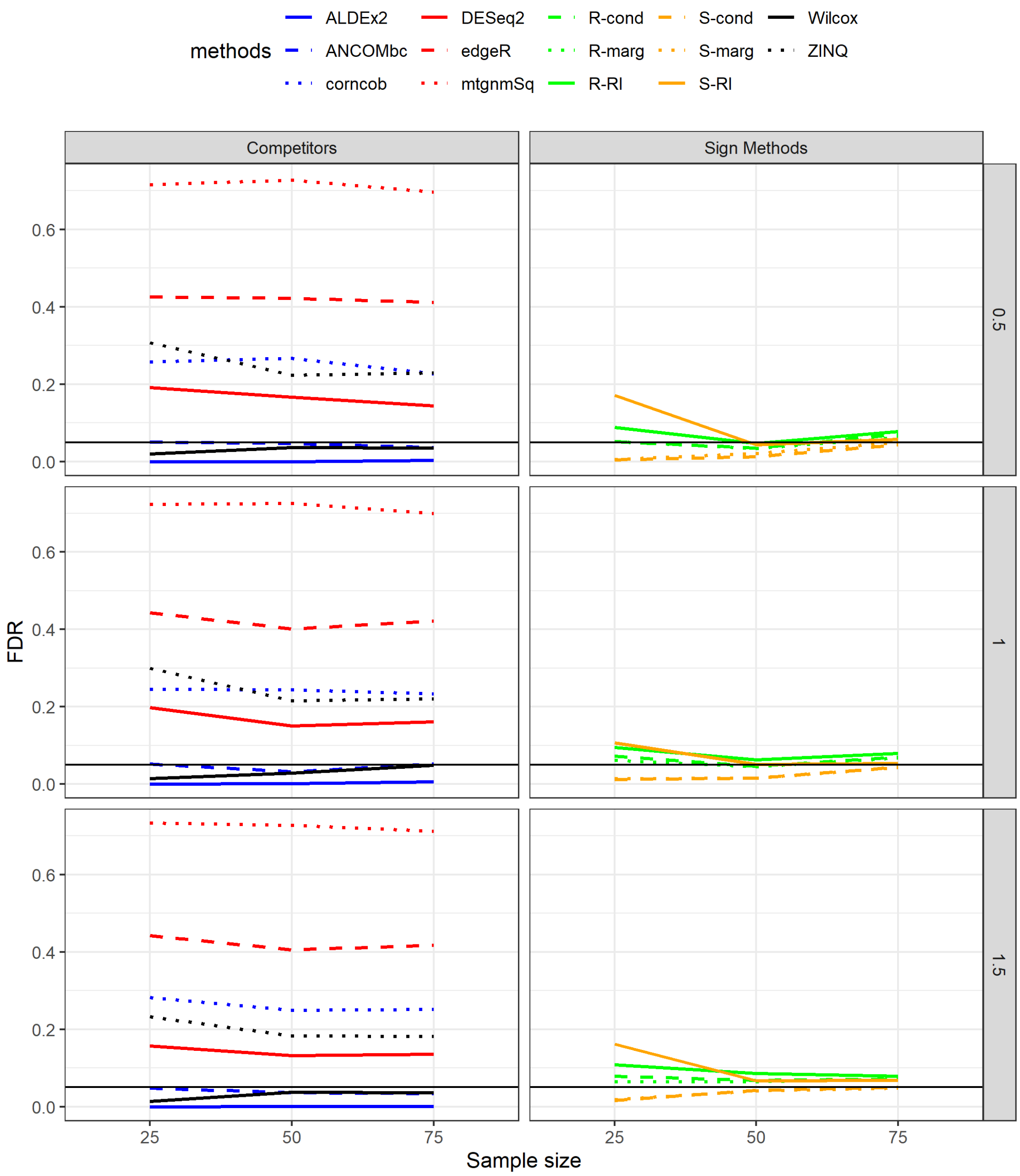

Figure 2: Empirical FDRs for the SPSimSeq simulations for setting A (high sparsity) for the new methods (left) and the competitors (right) with increasing sample size (25,50,75) and increasing log-fold change (0.5,1,1.5) and 10% DA. The nominal FDR was set on 0.05 (solid line).

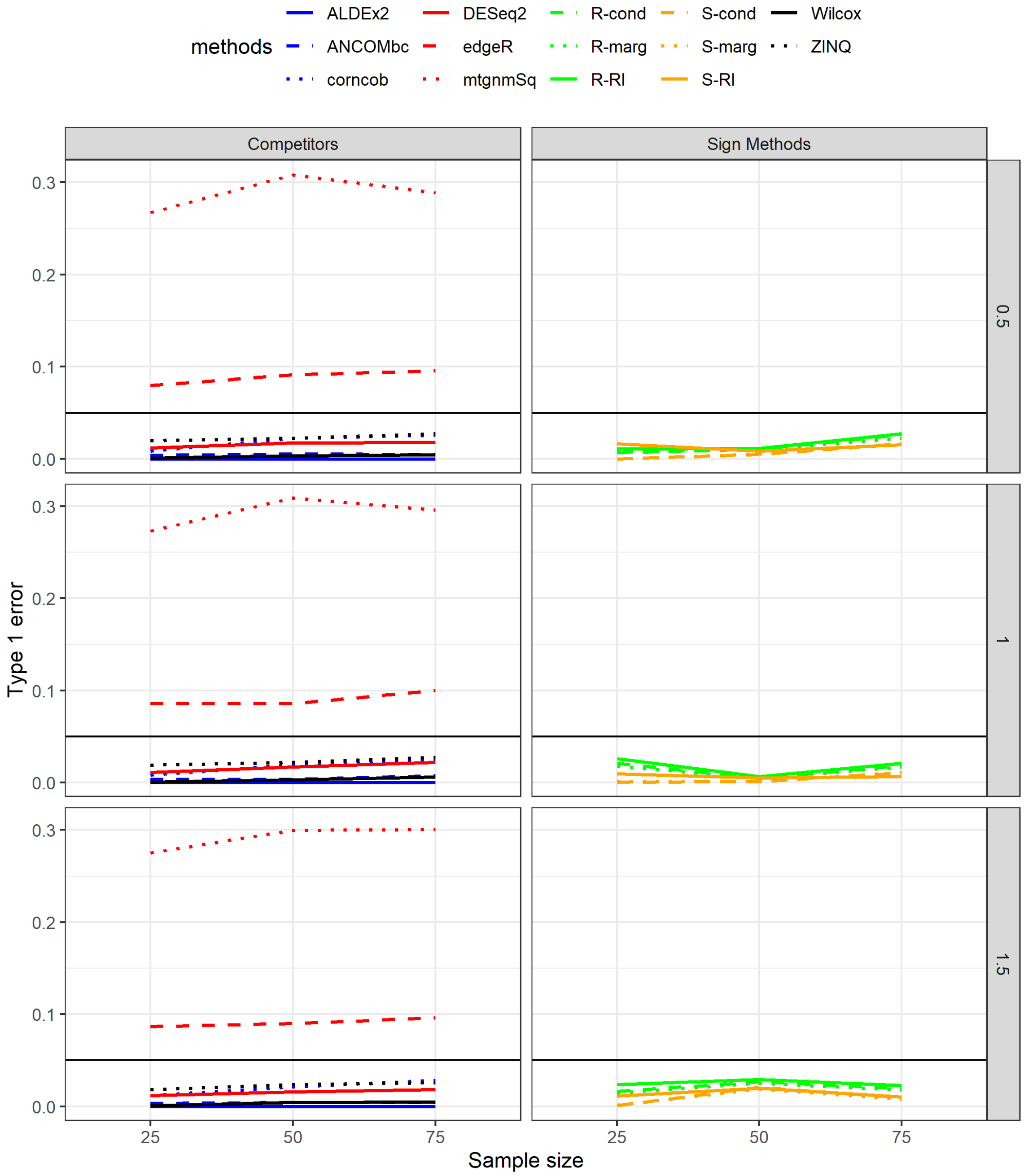

Figure 3: Empirical Type 1 error rate for the SPSimSeq simulations for setting A (high sparsity) for the new methods (right) and competitors (left) with increasing sample size (25,50,75),increasing log-fold change (0.5,1,1.5) and 10% DA.

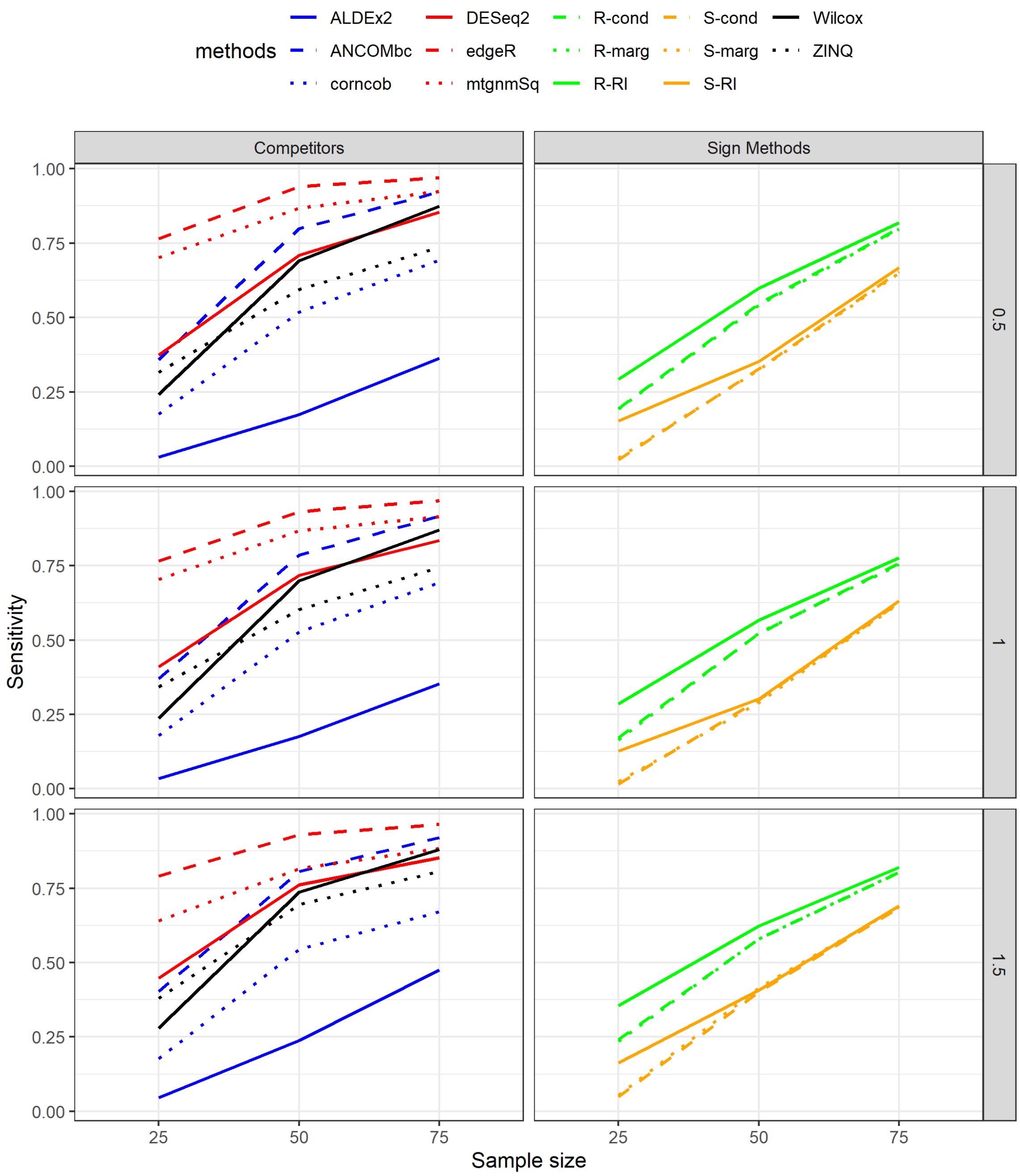

Figure 4: Empirical sensitivities for the SPSimSeq simulations for setting B (low sparsity) for the new methods (left) and the competitors (right) with increasing sample size (25,50,75) and increasing log-fold change (0.5,1,1.5) and 10% DA.

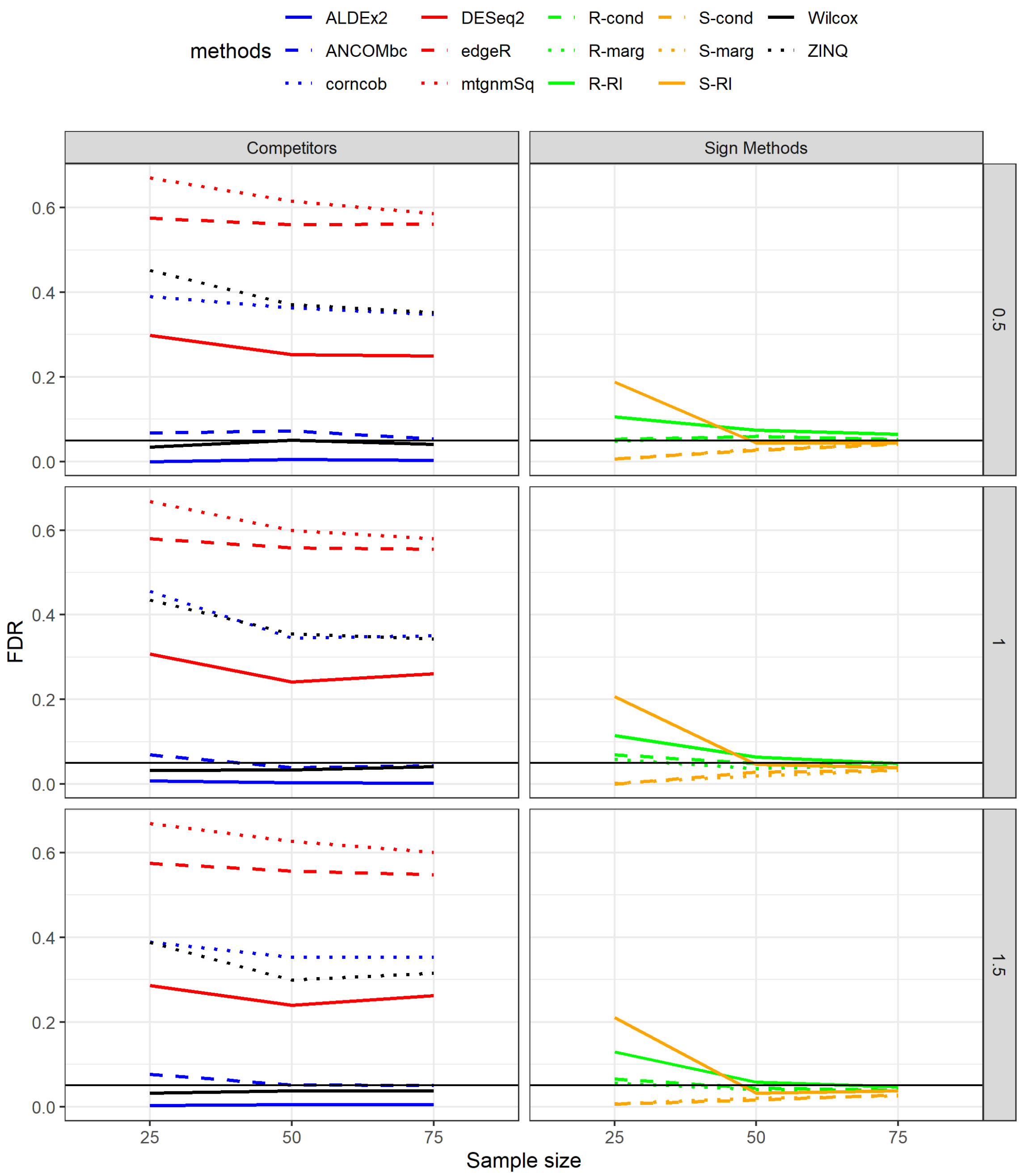

Figure 5: Empirical FDRs for the SPSimSeq simulations for setting B (low sparsity) for the new methods (left) and the competitors (right) with increasing sample size (25,50,75) and increasing log-fold change (0.5,1,1.5) and 10% DA. The nominal FDR was set on 0.05 (solid line).

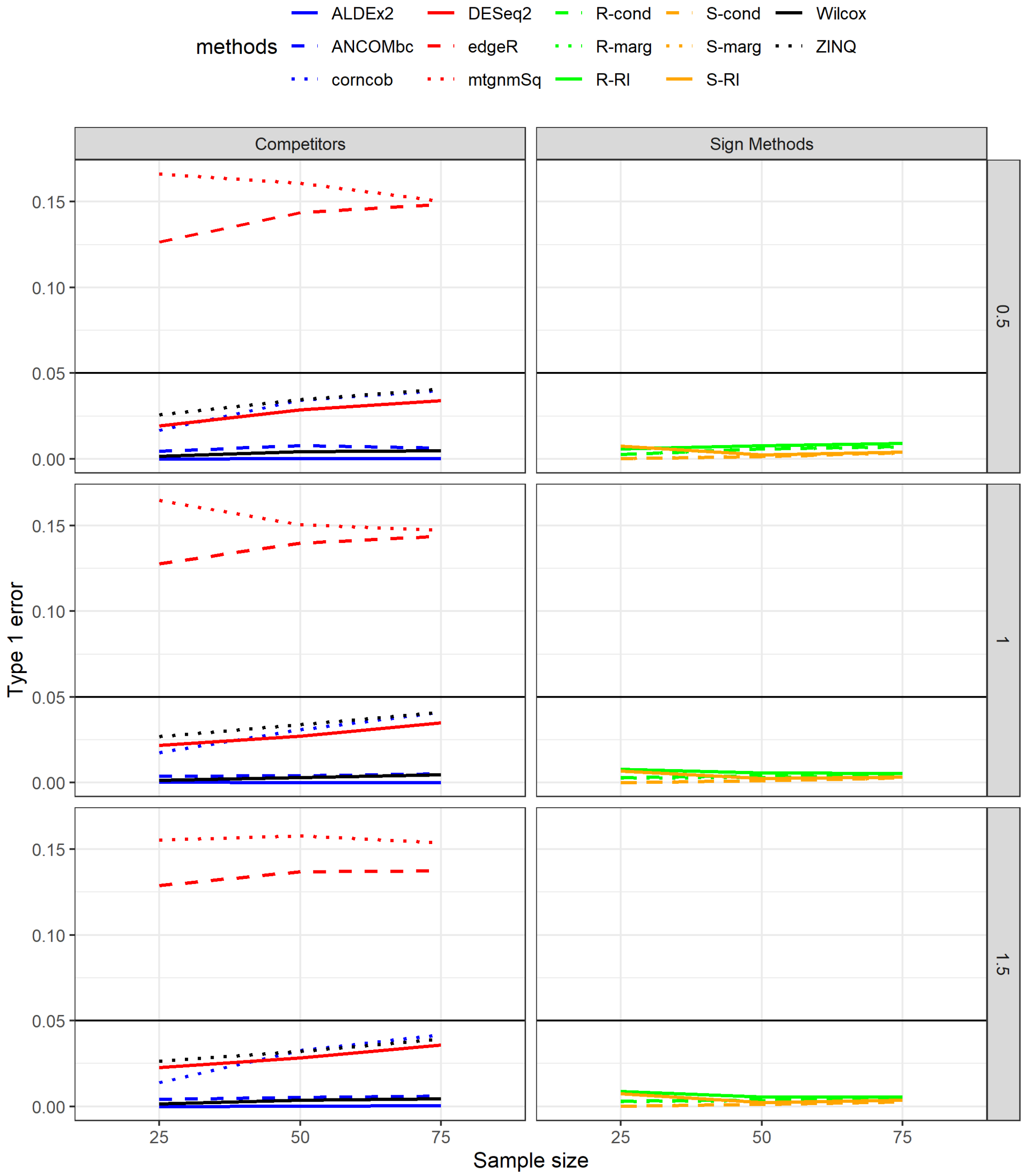

Figure 6: Empirical Type 1 error rate for the SPSimSeq simulations for setting B (low sparsity) for the new methods (right) and competitors (left) with increasing sample size (25,50,75),increasing log-fold change (0.5,1,1.5) and 10% DA.

Table 1: Reference frame (RF) description for all simulation scenarios of setting A. The first column depicts the number of simulated datasets where no RF was found. The second column shows the average length of the RF and the third column shows the number of RFs with at least one DA taxa. The last column shows the number of RFs which contain only DA taxa.

Scenario	No RF	Average RF length	RF with DA taxa	Complete
1.1	0	3.65	20	0
1.2	0	3.54	19	0
1.3	0	3.33	8	0
2.1	0	3.52	22	0
2.2	0	3.48	15	0
2.3	0	3.51	11	0
3.1	0	3.41	39	0
3.2	0	3.56	9	0
3.3	0	3.34	14	0

Table 2: Reference frame (RF) description for all simulation scenarios of setting B. The first column depicts the number of simulated datasets where no RF was found. The second column shows the average length of the RF and the third column shows the number of RFs with at least one DA taxa. The last column shows the number of RFs which contain only DA taxa.

Scenario	No RF	Average RF length	RF with DA taxa	Complete
1.1	0	3.66	1	0
1.2	0	3.53	3	0
1.3	0	3.62	3	0
2.1	0	3.69	3	0
2.2	0	3.42	2	0
2.3	0	3.38	4	0
3.1	0	3.61	0	0
3.2	0	3.58	0	0
3.3	0	3.22	1	0

Diagnostic plots

To compare the simulated data with the source data the following comparison metrics were used:

- the distribution of mean, variance and coefficients of variation (CV) of taxa abundance levels,
- the relationship between the mean and variance and the mean and CV of taxa abundance levels,
- the distribution of the fraction of zero counts per taxon and its relationship with the mean abundance level,
- the distribution of the pairwise correlation coefficients between the taxa and samples.

Setting A

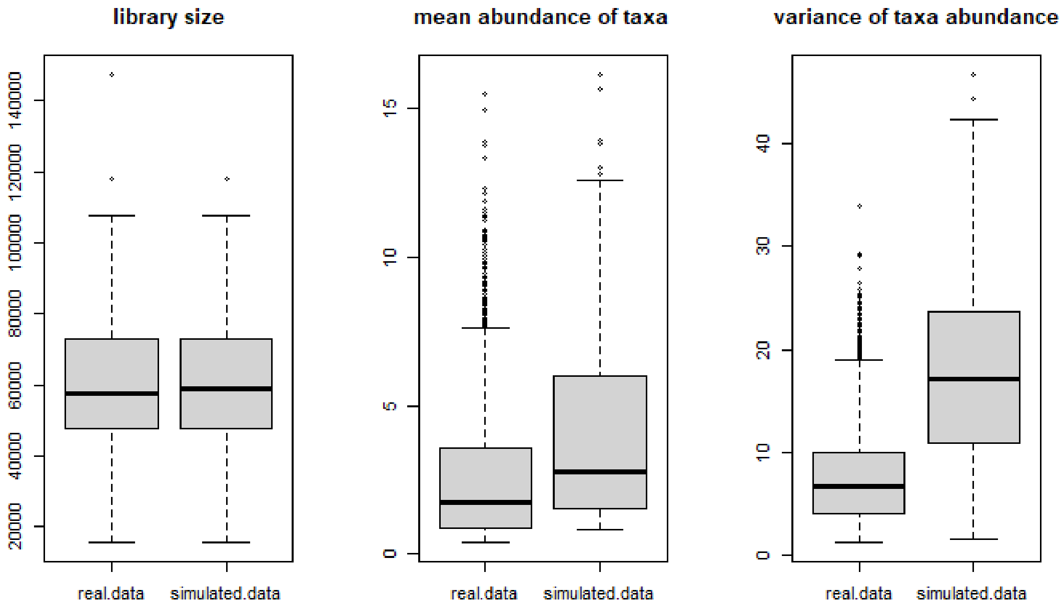

Figure 7: The distributions of the library size, mean and variance of taxa abundance from the pre-processed data (Source_A) and for simulated data of SPsimSeq (scenario 2.2).

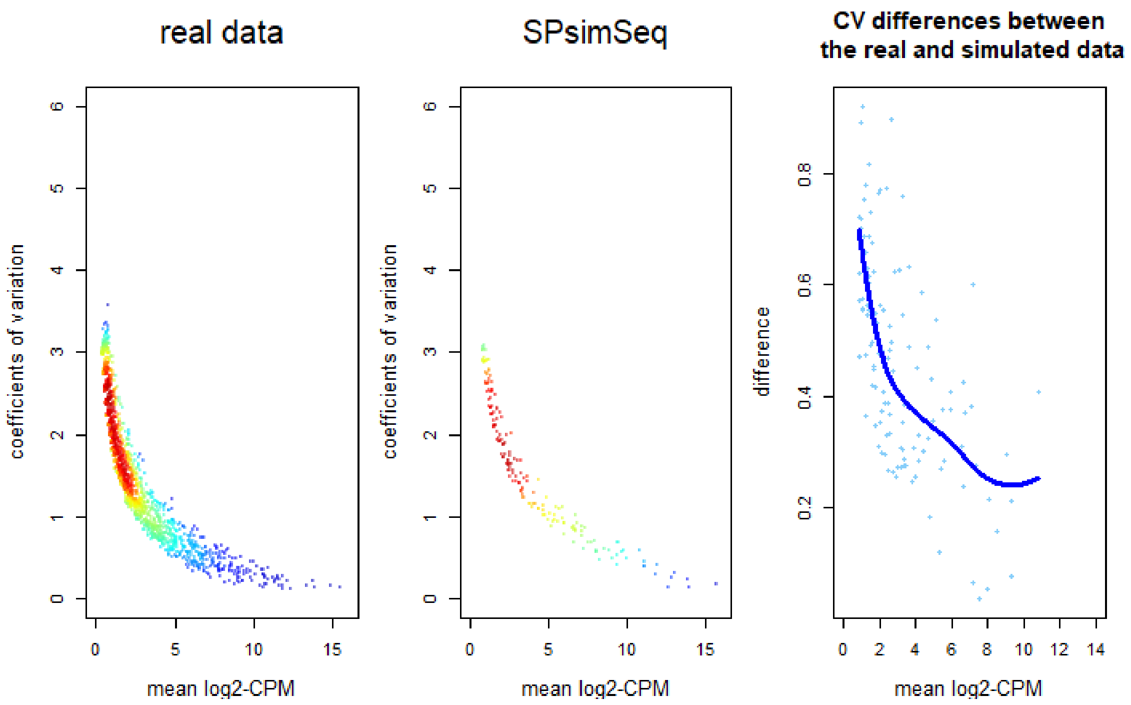

Figure 8: The relationship between the mean and coefficient of variation (CV) of taxa abundance levels (log-CPM) from the pre-processed data (Source_A) and for simulated data of SPsimSeq (scenario 2.2).

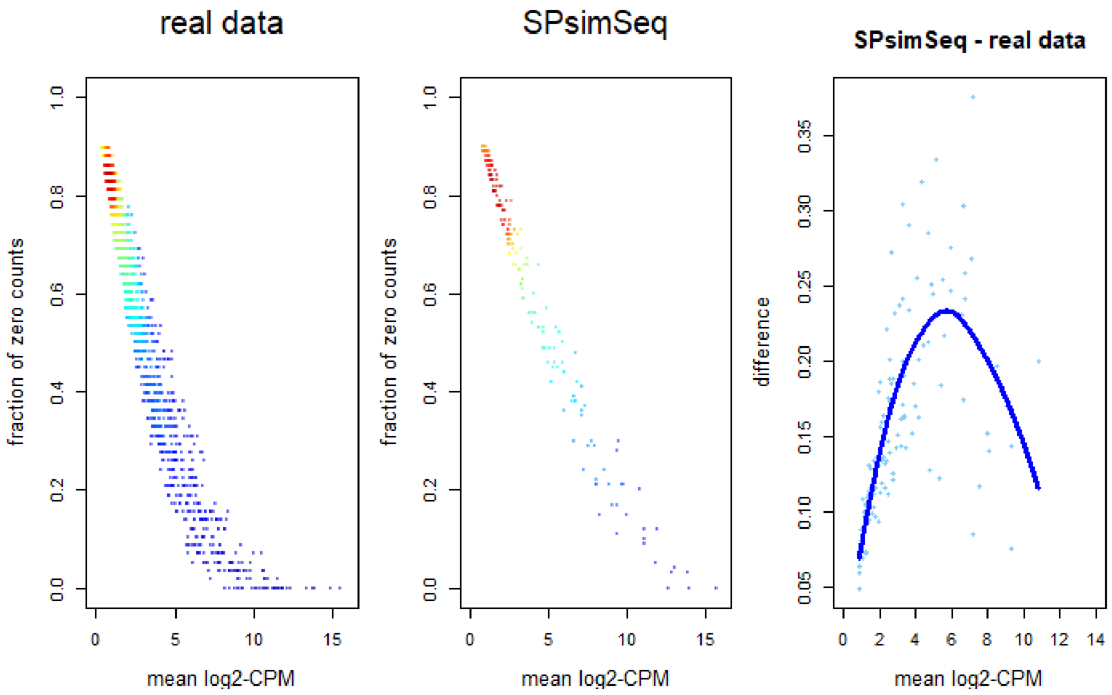

Figure 9: The fraction of zero counts per gene as a function of the mean taxa abundance levels (log-CPM) from the pre-processed data (Source_A) and for simulated data of SPsimSeq (scenario 2.2).

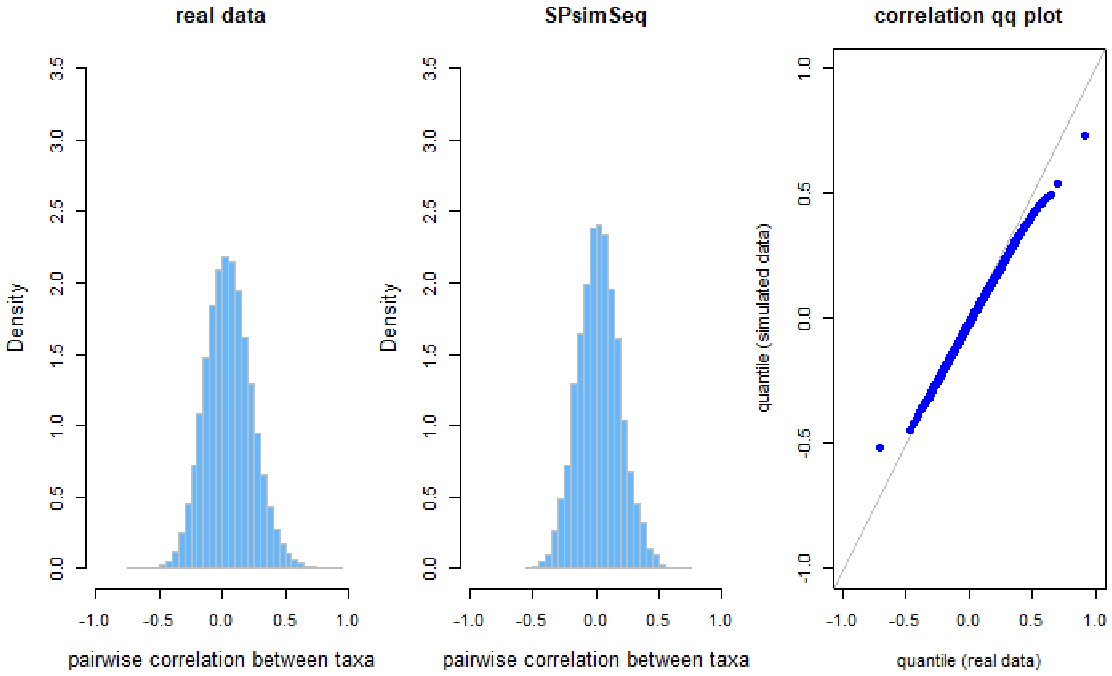

Figure 10: The distributions of the pairwise Pearson correlation-coefficients between taxa from the pre-processed data (Source_A) and for simulated data of SPsimSeq (scenario 2.2).

Setting B

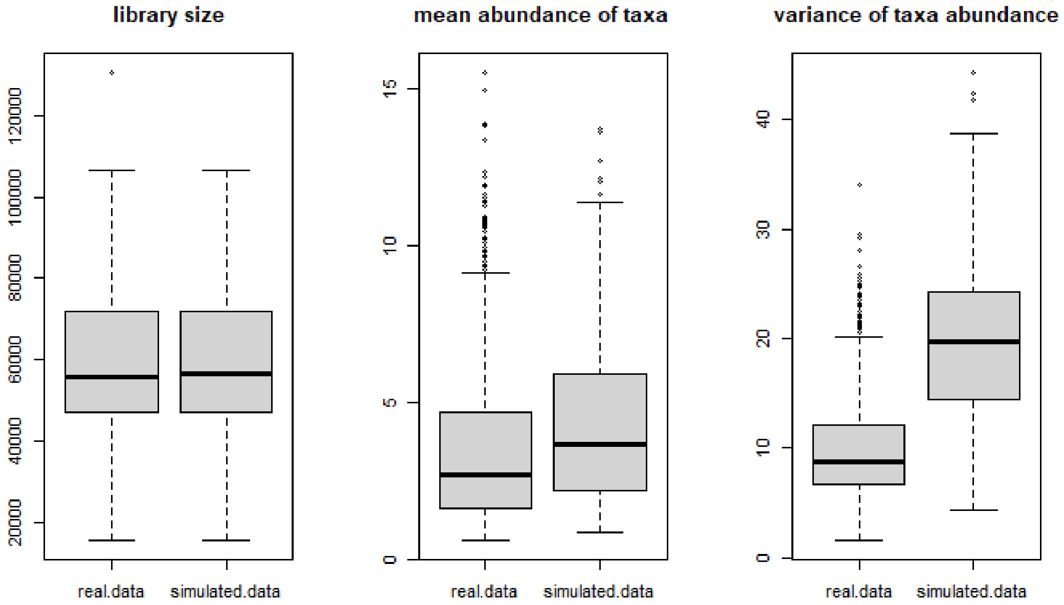

Figure 11: The distributions of the library size, mean and variance of taxa abundance from the pre-processed data (Source_B) and for simulated data of SPsimSeq (scenario 2.2).

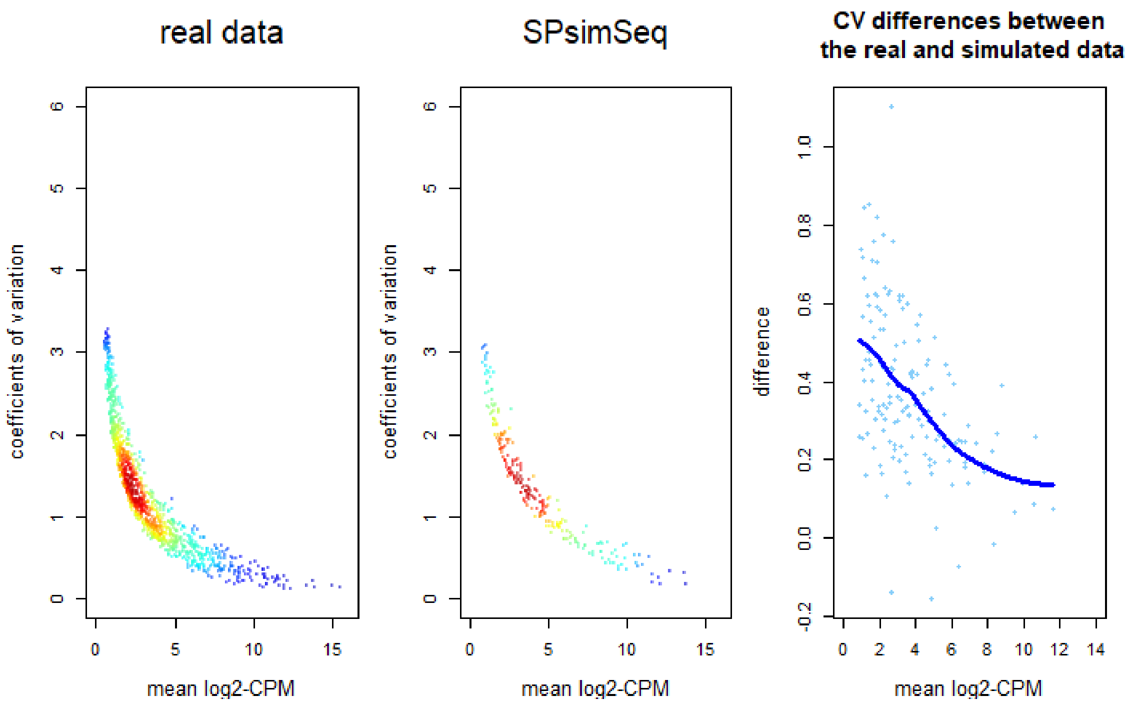

Figure 12: The relationship between the mean and coefficient of variation (CV) of taxa abundance levels (log-CPM) from the pre-processed data (Source_B) and for simulated data of SPsimSeq (scenario 2.2).

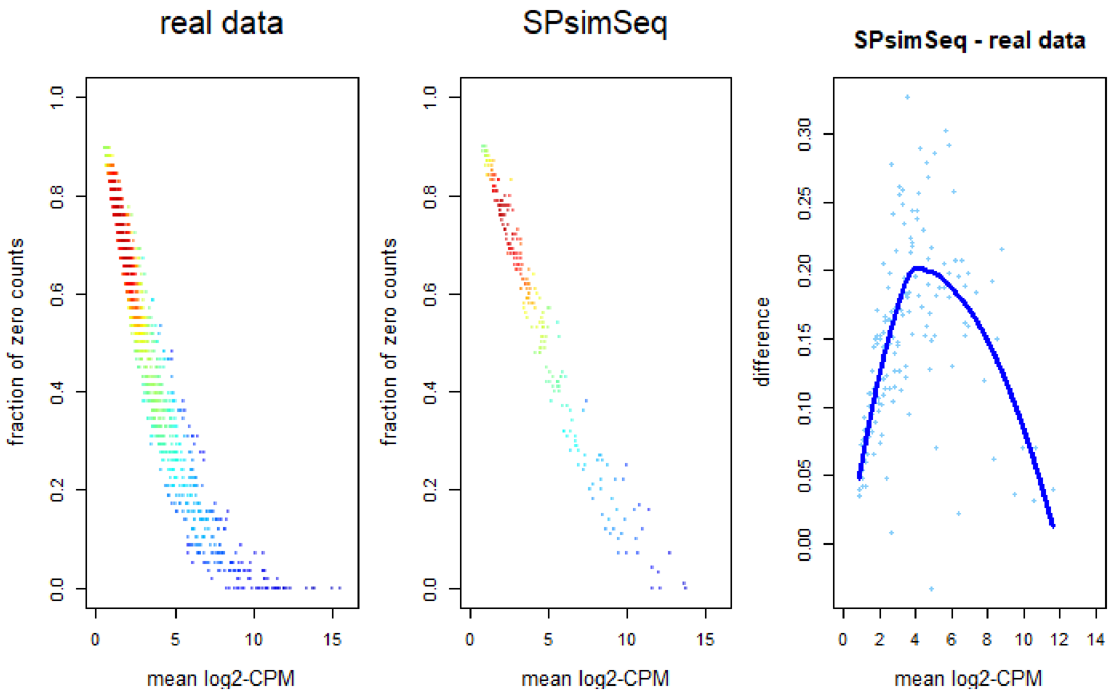

Figure 13: The fraction of zero counts per gene as a function of the mean taxa abundance levels (log-CPM) from the pre-processed data (Source_B) and for simulated data of SPsimSeq (scenario 2.2).

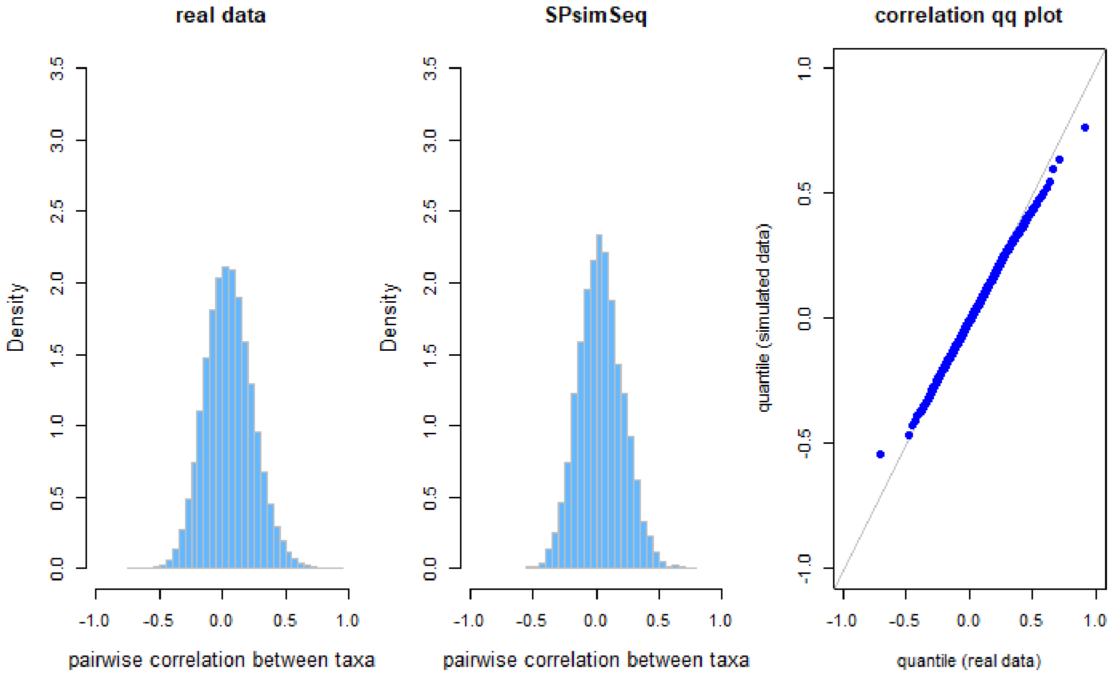

Figure 14: The distributions of the pairwise Pearson correlation-coefficients between taxa from the pre-processed data (Source_B) and for simulated data of SPsimSeq (scenario 2.2).

References

[1] Alice V Easton, Mayra Raciny-Aleman, Victor Liu, Erica Ruan, Christian Marier, Adriana Heguy, Maria Fernanda Yasnot, Ana Rodriguez, and P'ng Loke. Immune response and microbiota profiles during coinfection with plasmodium vivax and soil-transmitted helminths. *MBio*, 11(5):e01705–20, 2020.
